# On the Microstructure and Properties of Nb-18Si-6Mo-5Al-5Cr-2.5W-1Hf Nb-Silicide Based Alloys with Ge, Sn and Ti Additions (at.%)

**DOI:** 10.3390/ma13204548

**Published:** 2020-10-13

**Authors:** Jiang Zhao, Claire Utton, Panos Tsakiropoulos

**Affiliations:** 1 Department of Materials Science and Engineering, Sir Robert Hadfield Building, The University of Sheffield, Mappin Street, Sheffield S1 3JD, UK; zhaojiang6325@hotmail.com (J.Z.); c.utton@sheffield.ac.uk (C.U.); 2 College of Materials and Chemical Engineering, Key Laboratory of Inorganic Nonmetallic Crystalline and Energy Conversion Materials, Three Gorges University, Yichang 443002, China

**Keywords:** Nb-silicide-based alloys, complex concentrated alloys, high entropy alloys, alloy design, microstructures, oxidation, intermetallics, silicides

## Abstract

We studied the microstructures and isothermal oxidation of the Nb-silicide-based alloys Nb-11.5Ti-18Si-5Mo-2W-4.9Sn-4.6Ge-4.5Cr-4.7Al-1Hf (JZ4) and Nb-21Ti-18Si-6.7Mo-1.2W-4.4Sn-4.2Ge-4Cr-3.7Al-0.8Hf (JZ5), calculated their average creep rate for the creep goal conditions of T = 1200 °C and σ = 170 MPa, and compared properties of the two alloys with those of other refractory metal (RM) complex concentrated alloys (RCCAs). Both alloys had a density less than 7.3 g/cm^3^ and lower than the density of multiphase bcc solid solution + M_5_Si_3_ silicide RCCAs. There was macrosegregation of Si in both alloys, which had the same phases in their as-cast microstructures, namely βNb_5_Si_3_, αNb_5_Si_3_, A15-Nb_3_X (X = Al, Ge, Si, Sn), TM_5_Sn_2_X (X = Al, Ge, Si), C14-Cr_2_Nb, but no solid solution. After heat treatment at 1500 °C for 100 h, a low volume fraction of a W-rich (Nb, W)_ss_ solid solution was observed in both alloys together with βNb_5_Si_3_, αNb_5_Si_3_ and A15-Nb_3_X but not the TM_5_Sn_2_X, whereas the Laves phase was observed only in JZ4. At 800 °C, both alloys did not pest, and there was no spallation of their scales at 1200 °C. At both temperatures, both alloys followed parabolic oxidation kinetics and their weight changes were lower than those of Ti-rich Nb-silicide-based alloys. The oxidation of both alloys was superior to that of other RCCAs studied to date. For each alloy the Si macrosegregation, volume fraction of solid solution, chemical composition of solid solution and Nb_5_Si_3_, and weight changes in isothermal oxidation at 800 and 1200 °C that were calculated using the alloy design methodology NICE agreed well with the experimental results.

## 1. Introduction

Ultra-high temperature materials (UHTMs) are currently being developed as potential replacements of Ni-based superalloys to meet performance and environmental targets in future aero engines. Metallic UHTMs are refractory metal (RM) intermetallic composites (RMICs), RM high entropy alloys (RHEAs) and RM complex concentrated alloys (RCCAs) [1,2]. RMICs include Nb- or Mo-silicide-based alloys [3,4,5]. Nb-silicide-based alloys are attracting much attention for blade applications [1,3]. Some Nb-silicide-based alloys are also RCCAs [1].

Metallic UHTMs must meet property goals for creep, toughness and oxidation resistance [3,4,6]. The creep strength should be greater than 170 MPa at a creep rate of 2 × 10^−8^ s^−1^ at 1200 °C (for Nb-silicide-based alloys, this goal assumes density ρ = 7 g/cm^3^). The fracture toughness of critical components should be ≥20 MPa√m. The recession rate due to oxidation should be less than 0.25 μm/h at 1315 °C. The oxidation goal is derived from the requirement of achieving the oxidation life at 1315 °C of the second generation single crystal Ni-based superalloys at 1150 °C. The toughness goal requires metallic behaviour of the metallic UHTMs to distinguish them from engineering ceramics or ceramic UHTMs. Thus, owing to the toughness goal, the new metallic materials should have at least a small volume fraction of a ductile, metallic phase. The latter is the bcc Nb solid solution in the case of Nb-silicide-based alloys [1,4,7].

Research has ascertained the key role of specific alloying additions for meeting property goals in Nb-silicide-based alloys [3,7,8,9,10,11,12,13,14,15,16,17,18,19,20]. RM additions are indispensable for strength and creep, simple metal and metalloid element (SMME) and transition metal (TM) additions are needed for oxidation resistance and TMs and SMMEs are crucial for toughness (RM = Mo, Ta, W, SMME = Al, B, Ge, Si, Sn, TM = Cr, Hf, Ti) [1,20,21,22,23,24,25]. Processing can have a significant effect on toughness [1]. Nb-silicide-based alloys can meet the creep and toughness goals and have closed substantially the gap with the oxidation goal. It is not likely that a metallic UHTM could meet all three property goals [1,4,20,26].

Contamination of RMs and RM alloys by interstitials is well known [1,2,27,28,29,30,31,32,33] as is the pest oxidation phenomenon for RM alloys and compounds [27,28,29,30,31,34,35]. Nb-silicide-based alloys and RCCAs can suffer from pest oxidation and interstitial contamination [1,20]. Nb-silicide-based alloys that meet some property target(s) and/or are close to other(s) targets, i.e., alloys that offer an acceptable balance of properties to be considered candidate materials for use in aero engines, will require environmental coatings (EC) [3]. Most likely an EC will be made of metallic bond coat (BC)/thermally grown oxide (TGO)/ceramic top coat (TC) [36]. Two key prerequisites for the substrate alloy, allied with the oxidation goal, are the prevention of pest oxidation and scale spallation. It is desirable for the BC to form a well-adhering αAl_2_O_3_ scale as the TGO. The BC could consist of layers of different materials because it must also shield the substrate from interstitial contamination [36]. Nb-silicide-based alloys, including those that are also RCCAs, cannot form αAl_2_O_3_ scales [1,20]. The same seems to be the case for the other RCCAs studied to date [1,2].

In Nb-silicide-based alloys, the suppression of pest oxidation that is achieved with the addition of Ge or Sn depends on type and concentration of other alloying elements [37,38,39,40,41,42,43,44]. Individual additions of Ge or Sn in Nb-silicide-based alloys cannot suppress the spallation of their scales at high temperatures [41,42,43], in the case of Sn irrespective of its concertation in an alloy [37,38,39,41,42,44]. However, the oxidation behaviour changed when Ge and Sn were added simultaneously in Nb-silicide-based alloys.

In actual fact, Ge and Sn in synergy had the following effects:

(a) With Al, Cr and Ti, it suppressed pest oxidation at 800 °C and scale spallation at 1200 °C in the alloy Nb-24Ti-18Si-5Al-5Cr-5Ge-5Sn (OHS1) [45]);
(b) With Hf, Ta, Ti and W the synergy (i) did not suppress pest oxidation at 800 °C in the alloys Nb-12Ti-18Si-6Ta-2.5W-1Hf-2Sn-2Ge (JZ1) and Nb-12Ti-18Si-6Ta-2.5W-1Hf-5Sn-5Ge (JZ2) [46], and (ii) improved the adhesion of the scale when the Sn concentration was increased in the alloy JZ2 [46];
(c) With Al, Cr, Hf, Ta, Ti and W it (iii) suppressed pest oxidation at 800 °C and improved scale adhesion at 1200 °C in the alloy Nb-12Ti-18Si-6Ta-2.5W-1Hf-5Sn-5Ge-5Al-5Cr (JZ3) [47], the scale of which did not separate but was cracked along edges, (iv) did not suppress pest oxidation at 800 °C owing to micro-cracking of the alloy that was attributed to the high hardness of alloyed Nb_5_Si_3_ and the very low vol.% Nb_ss_, and (v) suppressed scale spallation at 1200 °C when the Sn concentration was increased in the alloy Nb-12Ti-18Si-6Ta-2.5W-1Hf-7.5Sn-5Ge-5Al-5Cr (JZ3+) [47]; Nb_5_Si_3_ alloyed with Ge is desirable for creep and oxidation resistance [6] (the nominal compositions of alloys are also given in the Abbreviations).

Remarkably, (1) the oxidation of the alloy JZ3+ at 1200 °C was better compared with the alloy OHS1 (parabolic oxidation of JZ3+ with rate constant only one order of magnitude higher than that of the single crystal superalloy CMSX-4) even though its Ti concentration was half that of OHS1 [45,47] and (2) calculated contribution of intrinsic resistances [1,20] to the creep of JZ3+ for the creep goal conditions was close to the creep target [47].

The research presented in this paper was inspired by our eagerness to find out if there are low density Nb-silicide-based alloys with satisfactory creep and oxidation compared with the single crystal Ni superalloy CMSX-4. Key research findings that informed this research were (i) the better creep of Nb-silicide-based alloys with RM additions compared with Ni-based superalloys [1,20,48,49], (ii) the superior solid solution strengthening of Nb by Mo compared with Ta [1,28,31], (iii) the elimination of scale spallation and/or pest oxidation when Sn and Ge were in synergy with Al, Cr and TM, or Al, Cr, TM and Ta,W additions [45,46,47] and (iv) the attainment of oxidation rates in the Ti-poor Nb-silicide-based alloys JZ3 and JZ3+ that are comparable with those of Ti-rich alloys [1,47].

The structure of the paper is as follows. In the next section, the alloy design/selection and its constraints is discussed and the nominal compositions of the selected alloys are given. The experimental details are then followed by the experimental results for the microstructures of the alloys and their isothermal oxidation at 800 and 1200 °C for 100 h. The discussion considers the densities of the alloys, the macrosegregation of Si, the as-cast and heat-treated microstructures and their oxidation. Alike [46,47], the experimental results are compared with the calculations (predictions) of NICE [20] throughout the discussion and similarly to [1] properties of the two alloys of this research, which are also RCCAs [2], are compared with those of other RCCAs studied to date. Furthermore, in the discussion of the results the two alloys of this study are compared with the Ta-containing alloys JZ1, JZ2 [46], JZ3 and JZ3+ [47]. A recommendation for future research is proposed at the end of the paper.

## 2. Alloy Design

The design/selection of the alloys used property targets for creep and oxidation and the alloy design methodology NICE [20]. The creep targets were creep rates (έ_alloy_) at 1200 °C and 170 MPa of (a) 4 × 10^−7^ s^−1^ and (b) 1.96 × 10^−6^ s^−1^ that are the experimental creep rates of the single crystal Ni-based superalloy CMSX-4, respectively, at 1200 °C and 50 MPa, and at 1200 °C and 100 MPa. Note that the creep property target in this research was different from that used in the design of the Ta-containing alloys JZ1, JZ2 [46], JZ3 and JZ3+ [47]. The oxidation targets were weight gains ΔW/A in isothermal oxidation at 800 and 1200 °C, respectively, of (c) 10 mg/cm^2^ and (d) 50 mg/cm^2^, the same as in [46,47].

**Figure 1:**
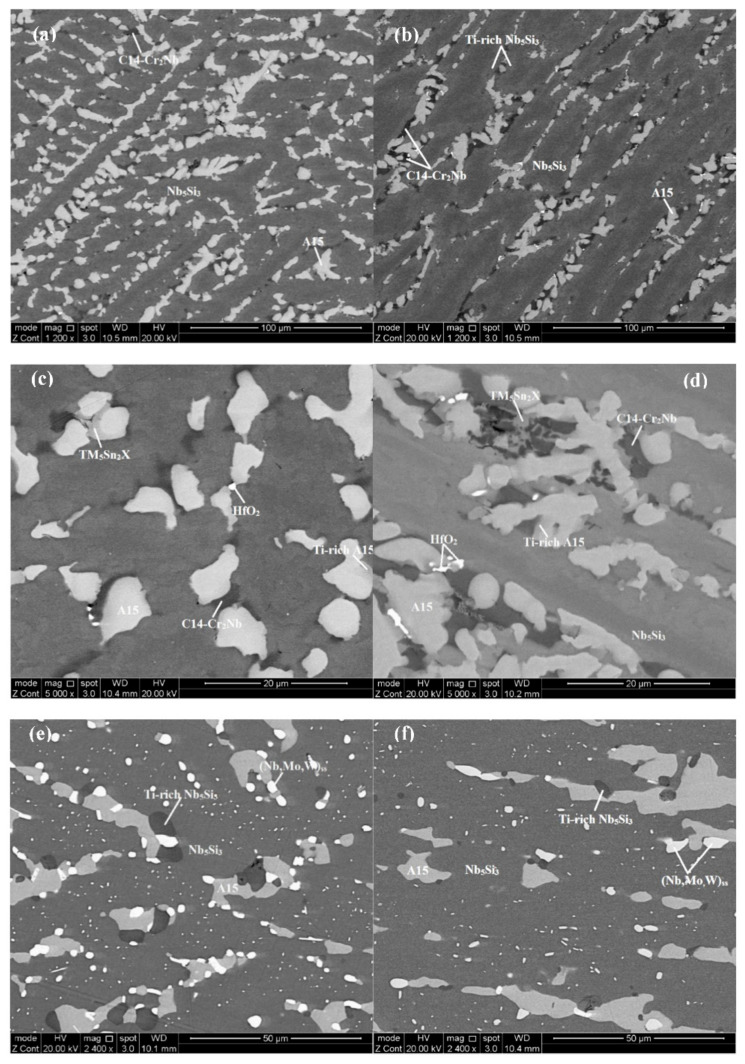
The as-cast (**a**–**d**) and heat-treated (**e**,**f**) microstructures of the alloys JZ4 (**a**,**c**,**e**) and JZ5 (**b**,**d**,**f**).

The constraints of the alloy design were as follows. The alloy(s) should (i) lie in the domain defined by the areas A and B in the Δχ versus δ map of Nb-silicide-based alloys and RCCAs, Figures 1 and 19, respectively, in [50] and [1], (ii) contain Al, Cr, Ge, Hf, Mo, Sn, Ti and W, (iii) have density lower than state-of-the-art Ni-based superalloys (ρ ≈ 9 g/cm^3^ for third generation, ρ ≈ 8.64 to 8.95 g/cm^3^ for second generation [51]), and lower than the density of multiphase bcc solid solution + M_5_Si_3_ silicide(s) RCCAs (7.78 ≤ ρ ≤ 8.46 g/cm^3^ [2]), (iv) contain Mo and W with Mo/W higher than the RM/W (RM = Mo,Ta) in the RCCAs studied to date (RM/W ≤ 1 [2]), (v) have Ti/Hf higher than that in the RCCAs studied to date (Ti/Hf ≤ 3 [2]) and (vi) have Si macrosegregation (MACSi) less than 5 at.%.

**Figure A1:**
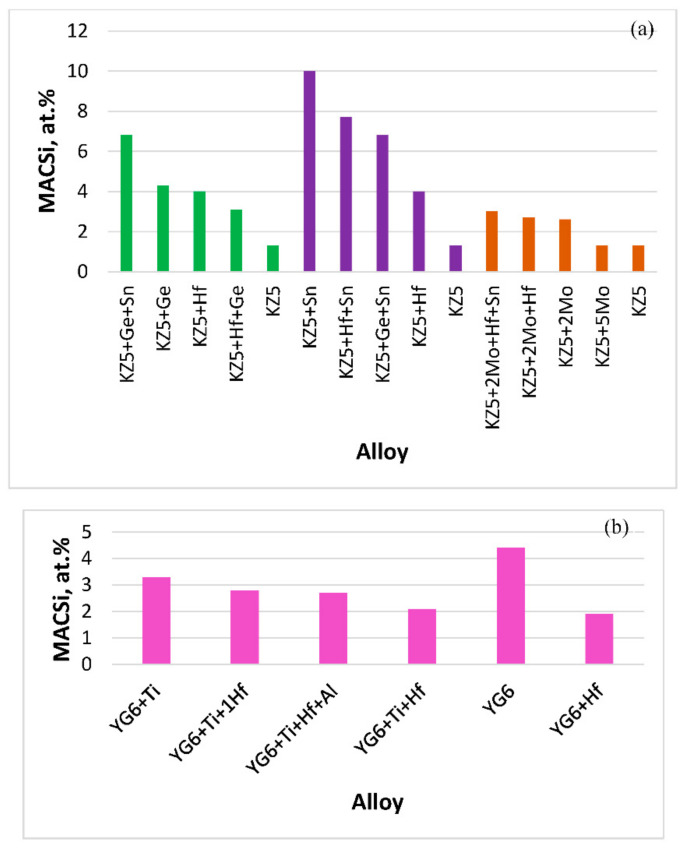
Si macrosegregation (MACSi) in (**a**) Ti-rich (Ti concentration about 24 at.%) and (**b**) Ti-poor (Ti concentration about 11 at.%) Nb-silicide-based alloys. Base alloy in (**a**,**b**), respectively, KZ5 (Nb-24Ti-18Si-5Al-5Cr [1,61]) and YG6 (Nb-20Si-5Mo-3W) [1,53].

With reference to the above constraints, (i), (iv) and (v) were related to creep, (ii), (iv) and (v) were linked with (iii), and (ii) was linked with oxidation resistance. The constraint (vi) was prompted by data about MACSi (a) in the Ta-containing alloys JZ1, JZ2 [46], JZ3 and JZ3+ [47] and (b) in Nb-silicide-based alloys with/without addition of Ge or Sn or Ge + Sn that shows average MACSi of about 4.2 and 2.9 at.% for Ti-rich and Ti-poor Nb-silicide-based alloys, respectively (see Figure A1 in the Appendix A).

For each creep target (έ_alloy_) the value of the alloy parameter Δχ_alloy_ was calculated from έ_alloy_ = g_1_(Δχ_alloy_) [20], and the relationships C_i_^alloy^ = ψ_i_(Δχ_alloy_) [20] that link the concentrations of alloying addition C_i_^alloy^ with Δχ_alloy_ were used to calculate the concentration of each element i. The values of Δχ_alloy_ were 0.1815 or 0.1725, respectively, for creep targets (a) and (b). Similarly, for each oxidation target (ΔW/A) the parameters VEC, δ and Δχ, and the ratio Nb/(Ti + Hf) were calculated from (ΔW/A)_Ti_ = f_j,Ti_(j^cal^_alloy_) [20], where j = VEC, δ, Δχ, Nb/(Ti + Hf)), the subscript T is the temperature and i is 800 or 1200 °C.

For the creep target (a), and the oxidation targets (c) and (d) the alloy design methodology NICE gave the alloy composition (at.%) Nb-11.5Ti-18Si-5Mo-2W-4.9Sn-4.6Ge-4.5Cr-4.7Al-1Hf, with ratios Mo/W = 2.5, Sn/Ge = 1.07, Al/Cr = 1.04 and Ti/Hf = 11.5 that are not significantly different from those in the alloy JZ3+ [47]. We shall call this alloy JZ4. For the property targets (b), (c) and (d), the alloy design methodology NICE gave the alloy Nb-21Ti-18Si-6.7Mo-1.2W-4.4Sn-4.2Ge-4Cr-3.7Al-0.8Hf with ratios Al/Cr = 0.93, Sn/Ge = 1.05, Mo/W = 5.6, Ti/Hf = 26.2, with the former two essentially the same and the latter two more than double the corresponding ratios in the alloys JZ3+ [47] and JZ4. We shall call the second alloy JZ5. This alloy was expected to have lower T_Liquidus_ compared with JZ4, owing to its higher Ti concentration. Note that the Nb/(Ti + Hf) ratios of the alloys JZ4 and JZ5, respectively, of 3.5 and 1.65, are consistent with the empirical creep rule according to which the secondary creep rate of Nb-silicide-based alloys increases as this ratio decreases [6,20,49].

## 3. Experiment

The alloys were made with high purity Al, Cr, Ge, Hf, Mo, Nb, Si, Sn, Ti and W elements (better than 99.99 wt.%) supplied by Goodfellow using arc melting in an argon atmosphere with a non-consumable tungsten electrode and a water-cooled copper crucible. Each alloy was melted five times to achieve chemical homogeneity. Similarly to the alloys JZ1, JZ2 [46], JZ3 and JZ3+ [47], it was very difficult to make the alloys with their compositions close to the nominal ones (given in the previous section), owing to the loss of elements by evaporation during arc melting (note that loss of Ge during melting also has been reported in [6]). The alloy JZ4 was prepared with a composition as close as possible to the nominal one after three attempts, where losses were compensated with an increase in the weight of elemental additions. The alloy JZ5 was prepared with a composition close to the nominal one using elemental TM and RM additions and ingots of the alloys Nb-24Ti-18Si-5Al-5Cr-5Mo (JG2 [52]), Nb-24Ti-18Si-5Al-5Cr-5Sn (ZX8 [42]), Nb-18Si-5Ge (ZF1 [24]), Nb-20Si-5Mo-3W (YG6 [53]) as master alloys.

Specimens from the top, bulk and bottom areas of each alloy button were mounted in Bakelite, ground using 120, 400, 800 and 1200 grit papers, and polished to a 1-μm surface finish using diamond pastes. These specimens were used for the SEM studies and EDS analyses (see below). According to DSC experiments (data not shown) the alloys did not exhibit melting up to 1600 °C. Specimens from the bulk of the buttons were wrapped in Ta foil, placed in an alumina crucible, and heat treated at 1500 °C for 100 h in an alumina tube furnace under a flow of Ti-gettered argon. The specimens were cooled in the furnace.

We used a Siemens D5000 X-ray diffractometer with monochromatic CuKα radiation and Powder Diffraction File data with the ICDD PDF-4+ and Sieve + software to identify the phases in as-cast and heat-treated alloys. The microstructures were studied using back-scattered electron (BSE) imaging in an Inspect F SEM. The chemical composition of large areas from the top, bulk and bottom of each alloy button and of the phases in these areas were analysed using JEOL 6400 SEM and Philips XL 30S FEG SEMs equipped with energy dispersive X-ray spectrometry (EDS) and a voltage of 20 kV. At least five EDS analyses of phases with sizes larger than 5 μm and of large areas were performed. The EDS data are given with the average, minimum and maximum values and standard deviation. EDS standards were specimens of high purity Nb, Ti, Si, Hf, Mo, W, Ge, Sn, Al, Cr and Al_2_O_3_ that were polished to a 1-μm finish. A specimen of pure Co was used to calibrate the EDS detector. Calibration was repeated every hour during analysis. Area fractions of the Nb_ss_ and A15 phases were calculated using the software Image-Pro with BSE images taken in the Inspect F SEM.

The isothermal oxidation of each alloy at 800 °C and 1200 °C was studied for 100 h using thermal gravimetric (TG) analysis (NETZSCH STA 449 F3 thermal analyser with the rate of 3 °C per minute in both heating and cooling) and 3 × 3 × 3 mm^3^ specimens from the as-cast alloys that were ground to 1200 grit. There was no evidence of micro-cracking in the as-cast buttons and in the as-prepared oxidation specimens of both alloys. An AccuPyc II 1340 gas pycnometer was used to measure the density of the alloys.

## 4. Results

### 4.1. Microstructures

The densities of the as-cast alloys and the area % of the Nb_ss_ and A15-Nb_3_X (X = Al, Ge, Si, Sn) in the as-cast and heat-treated alloys are given in the Table 1. The density and the vol.% of the aforementioned phases of the alloy JZ4 were higher than those of the alloy JZ5.

The average chemical compositions of the alloys and of the phases in their microstructures are given in the Table 2 (compare with the microstructures of the alloys JZ3 and JZ3+ in Table 2 in [47]). The chemical analysis data of the as-cast (AC) and heat-treated (HT) alloys is given in the Tables S1 and S2 in the Supplementary Materials. Compared with the nominal alloy compositions, in the cast alloy JZ4 (JZ4-AC) the concentrations of Cr, Ge, Mo, Sn and W were slightly higher, and the ratios Mo/W, Sn/Ge, Al/Cr and Ti/Hf (respectively, 2.6, 1.07, 1.04 and 11.5) essentially the same, whereas in the cast alloy JZ5 (JZ5-AC) the concentrations of Al, Cr, Ge, Si and Sn were slightly higher, and the ratios Mo/W, Sn/Ge, Al/Cr and Ti/Hf (respectively, 5.7, 1.1, 0.96, 25.5) were actually the same. There was macrosegregation of Si (MACSi) in both alloys, 3 and 4.5 at.%, respectively, in JZ4-AC and JZ5-AC.

The same phases were present in the cast microstructures of both alloys, namely Nb_5_Si_3_, A15-Nb_3_X, TM_5_Sn_2_X, C14-Cr_2_Nb and HfO_2_; see Table 2 and Tables S1 and S2 and Figures S1 and S2 in the Supplementary Materials. The Nb_5_Si_3_ silicide existed in both the β and α forms. In both alloys, Ti-rich Nb_5_Si_3_ and Ti-rich A15-Nb_3_X were also observed. The XRD did not confirm the presence of Nb solid solution. The absence of the latter was also confirmed by EDS in both alloys. In the bottom of the button of JZ4-AC the TM_5_Sn_2_X and Ti-rich Nb_5_Si_3_ and Ti-rich A15-Nb_3_X were not observed.

The typical microstructure of JZ4-AC is shown in Figure 1a,c and the EDS data are given in Table S1. The microstructures in the top and bulk of JZ4-AC were similar. The Mo solubility in the silicide was 4.5 at.%. The areas exhibiting lighter contrast at the edges of Nb_5_Si_3_ grains were rich in Ti and had relatively lower concentration of Si (about 20.8 at.%) and higher concentration of Sn (about 5 at.%) than the bulk of the grains. The <Si> (=Si + Sn + Ge + Al) content in the “normal” and Ti-rich Nb_5_Si_3_ was 37 at.% and 36.2 at.%, respectively. The A15 was formed between the Nb_5_Si_3_ grains. The Mo solubility was the same (14.2 at.% and 14.4 at.%) in the “normal” and the Ti-rich A15 and the <Si> content was 19.8 at% and 23.8 at.%, respectively.

In between the primary silicide grains, a phase was observed that exhibited a similar contrast to that of the Ti-rich A15. The composition of this phase corresponded to the Nb_5_Sn_2_Si intermetallic, in agreement with the XRD data (Figure S1). Only two analyses of this phase were possible owing to the size. In this compound the Nb was substituted by Ti and the Si by Al and Ge. Considering its relatively high concentrations of Ti, Ge and Al and the fact that Nb_5_Sn_2_Si, Nb_5_Sn_2_Ge, Nb_5_Sn_2_Al and Ti_5_Sn_2_Si all have the W_5_Si_3_ as prototype [54], this phase is given as TM_5_Sn_2_X in Table S1, where TM = Nb, Ti and X = Si, Ge, Al. The composition of the Laves phase was in agreement with [21].

The microstructure of JZ5-AC is shown in Figure 1b,d. There were no differences in the microstructure in the top, bulk and bottom of the button. The partitioning of elements between the Nb_5_Si_3_ and A15 was the same as in JZ4-AC, with increased concentrations of Mo, W and Cr in the A15. The <Si> content in the “normal” and Ti-rich Nb_5_Si_3_ was essentially the same as in JZ4-AC and the content in the “normal” and Ti-rich A15 was slightly higher than that in JZ4-AC. The C14-Cr_2_Nb Laves phase was slightly richer in Ti and leaner in Nb, Cr and W compared with JZ4-AC. The TM_5_Sn_2_X compound was richer in Ti. Two analyses of this phase were possible (Table S2). The microhardness of Nb_5_Si_3_ was 1445 ± 50 HV compared with 1508 ± 41 HV in JZ4-AC. The hardness of the cast alloys JZ4 and JZ5 was 862 HV and 838 HV, respectively.

After the heat treatment there was no chemical inhomogeneity of Si in both alloys. In the alloy JZ4-HT the ratios Mo/W, Sn/Ge, Al/Cr and Ti/Hf, respectively, were 2.6, 1, 1 and 10.3, and in the alloy JZ5-HT they were 5.2, 1, 0.96 and 20.5, respectively, and were not different compared with the cast alloys with the exception of the Ti/Hf ratio which was slightly reduced. The same phases were observed in the heat-treated microstructures, namely Nb_5_Si_3_, A15-Nb_3_X, (Nb,W)_ss_ and HfO_2_ (Table 2 and Tables S1 and S2 and Figures S1 and S2 in the Supplementary Materials). A very small volume fraction of the C14-Cr_2_Nb Laves phase was observed only in JZ4-HT. The Nb_5_Si_3_ silicide existed in both the β and α forms and Ti-rich Nb_5_Si_3_ was also observed. The vol.% of the A15 compound was reduced notably in JZ4-HT, and solid solution rich in W and Mo formed at a small volume fraction (Table 1).

The microstructure of JZ4-HT is shown in Figure 1e. In the Nb_5_Si_3_, the Si concentration was reduced and the solubility of Ti, Sn, Al and Cr was increased. In the Ti-rich Nb_5_Si_3_ the solubility of Sn was lower and the solubilities of Si and Hf were higher compared with the “normal” silicide. The <Si> content of the “normal” and Ti-rich Nb_5_Si_3_ was 36.6 at.% and 38.1 at.%, respectively. The solid solution was Si-free and very rich in W (29.4 at.%). It was formed around the Nb_5_Si_3_ silicide grains and as dispersed tiny particles in them. The Laves phase exhibited the same contrast as the Ti-rich Nb_5_Si_3_, its Si and Al contents, respectively, were increased and decreased by 2.5 at.% and 5 at.%, compared with JZ4-AC (Table S1). Remarkably, the alloy was not contaminated by oxygen. Only a very small volume fraction of alumina had formed just below the surface of the heat-treated specimen.

The microstructure of JZ5-HT is shown in Figure 1f. The solid solution exhibited white contrast, was Si free and very rich in Mo and W. Tiny particles of the solid solution were dispersed in the Nb_5_Si_3_ grains. The Si solubility in the Nb_5_Si_3_ was reduced to 20.3 at.% and the Sn, Al and Cr concentrations were increased, whereas in the Ti-rich Nb_5_Si_3_ there were higher concentrations of Si and Hf and lower concentration of Sn, compared with JZ5-AC. The <Si> content of the “normal” and Ti-rich Nb_5_Si_3_ was 36.8 at.% and 37.9 at.%, respectively (Table S2). There was a slight increase in the Cr content in the A15. There was no contamination of the JZ5-HT by oxygen.

Only one measurement of the hardness of (Nb,W)_ss_ was possible in JZ4-HT and JZ5-HT using nano-indentation. The hardness of the solid solution was similar (about 850 HV) in both alloys. The hardness of JZ4-HT and JZ5-HT was 834 HV and 806 HV, respectively.

### 4.2. Oxidation

**Figure 2:**
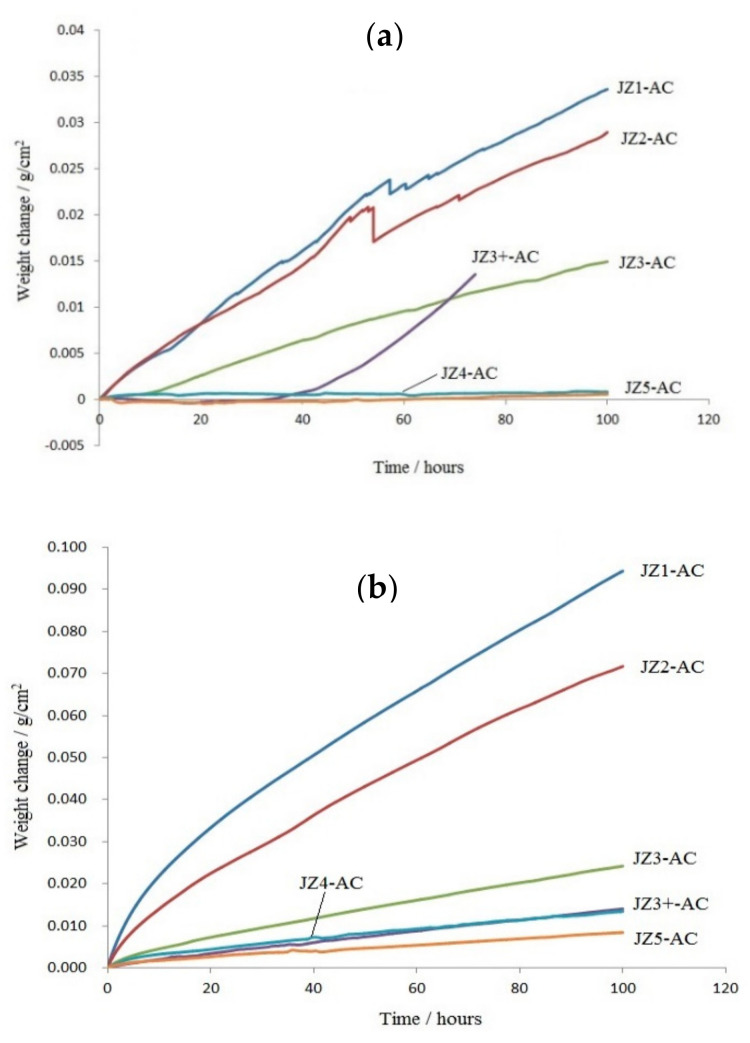
Thermal gravimetric (TG) data for the isothermal oxidation of the alloys JZ4 and JZ5 together with the data for the alloys JZ1, JZ2 [46], JZ3 and JZ3+ [47]. (**a**) 800 °C, (**b**) 1200 °C.

**Figure 3:**
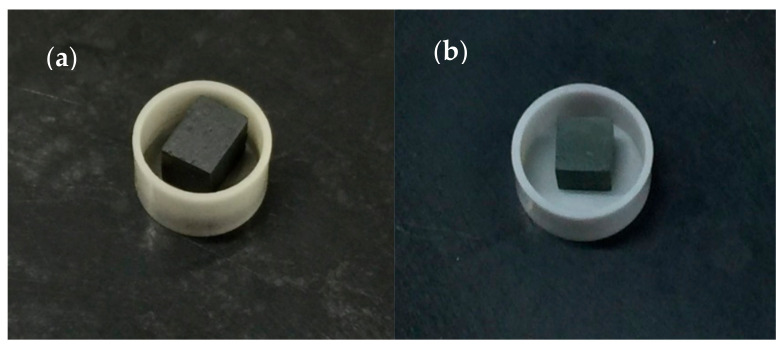

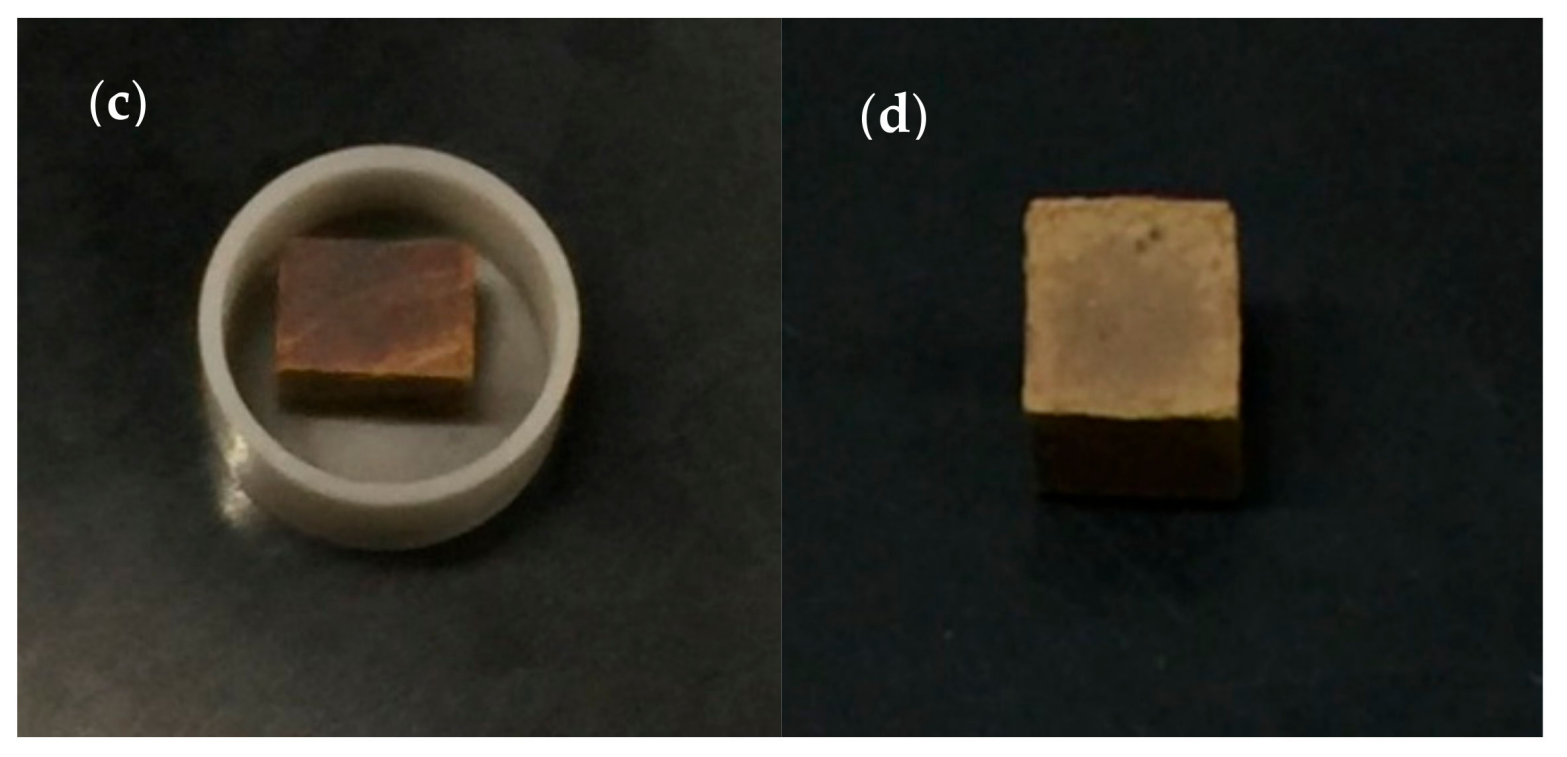
The specimens of the alloys JZ4 and JZ5 after isothermal oxidation at 800 and 1200 °C; (**a**) and (**c**) alloy JZ4, (**b**,**d**) alloy JZ5; (**a**,**b**) 800 °C, (**c**,**d**) 1200 °C.

The TG data are shown in Figure 2, the oxidised specimens in Figure 3 and the rate constants and weight changes of the alloys JZ4 and JZ5 are given in the Table 3. In Figure 2, the data for the Ta-containing alloys JZ1 to JZ3+ [46,47] are included for comparison purposes. The weight gains of the alloys JZ4 and JZ5 after 100 h at 800 °C were 0.82 mg/cm^2^ and 0.57 mg/cm^2^, respectively. Pest oxidation and spallation of oxide scales did not occur. Both alloys followed parabolic oxidation kinetics at 800 °C. The oxidation of the alloy JZ4 at 1200 °C was similar to that of the alloy JZ3+ [47], and the weight gain was 13.43 mg/cm^2^. In the case of the alloy JZ5, the weight gain of 8.43 mg/cm^2^ was the lowest of all the JZ series of alloys. No spallation of the oxide scales was observed in the alloys JZ4 and JZ5.

#### 4.2.1. Oxidation at 800 °C

**Figure 4:**
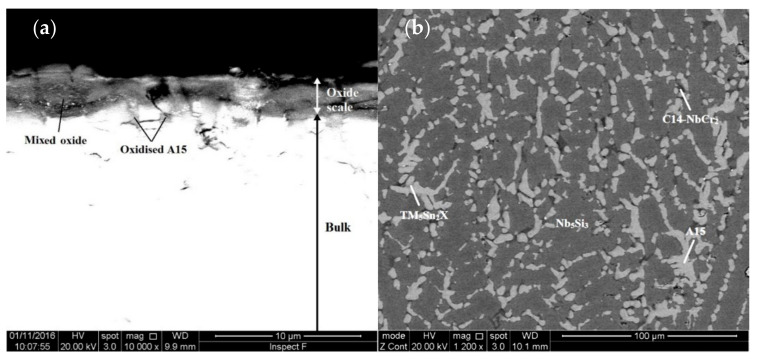

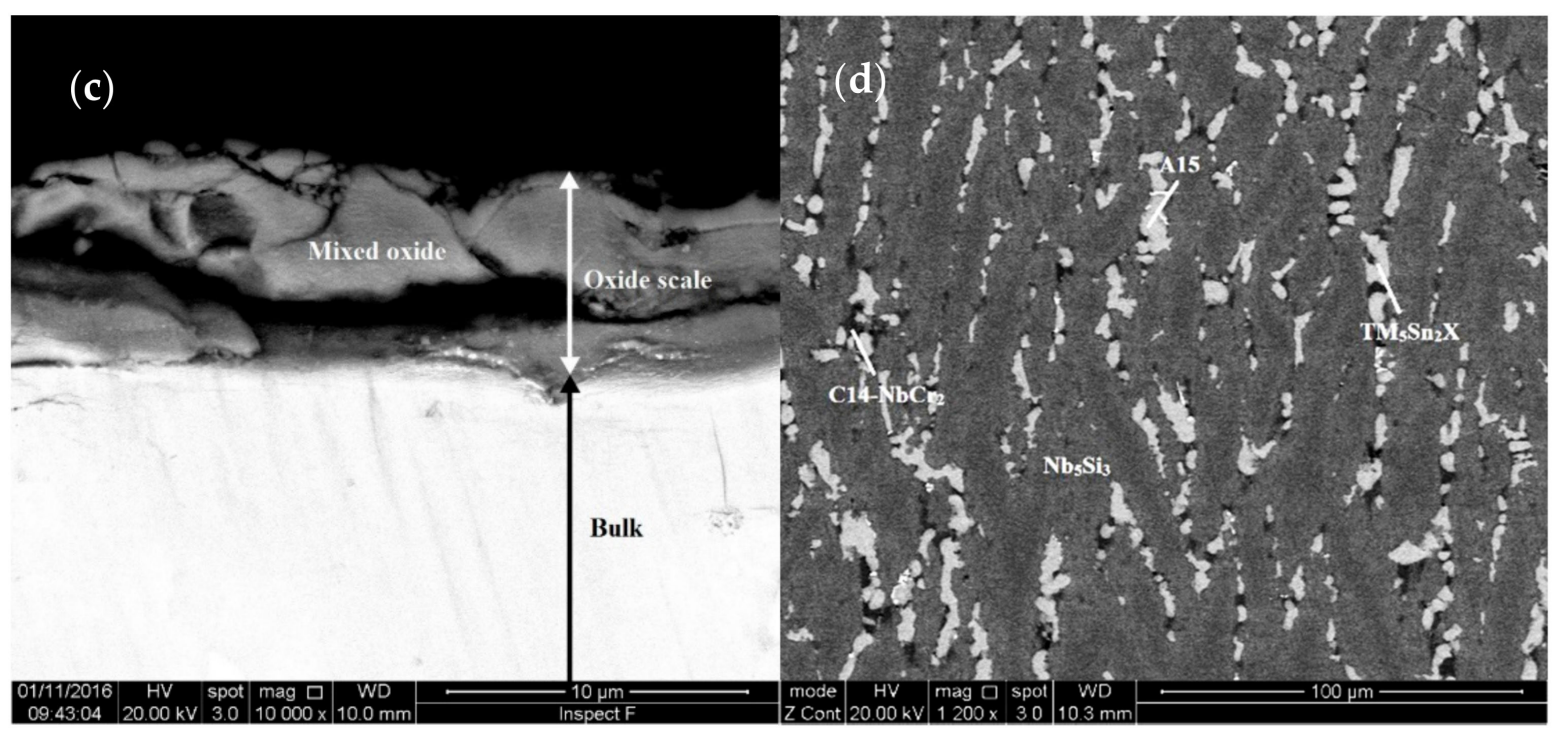
Back-scattered electron (BSE) images of the microstructure of cross sections of the oxidised alloys JZ4 (**a**,**b**) and JZ5 (**c**,**d**) at 800 °C (**a**,**c**) scale, (**b**,**d**) bulk microstructure. Enhanced contrast in (**a**,**c**) to show oxide scale.

Figure 4a,b show the oxide scale and bulk of a cross section of the alloy JZ4 after the TG experiment. The thickness of the scale was about 3 μm. In the scale there were some short cracks parallel to its surface. Table S3 in the Supplementary Materials gives the chemical composition of the scale and of the phases in the bulk. The latter phases were the same as those in JZ4-AC, with the exception of the Ti-rich A15 that was not observed. There was no apparent change of the compositions of the phases compared with the JZ4-AC. The mixed oxide that formed the oxide scale was rich in Nb and Si and very lean in other elements. There was some internal oxidation in a very thin area below the scale where the oxidised A15 phase exhibited a darker contrast, see Figure 4a. Below this area the microstructure was not contaminated by oxygen.

The microstructure of a cross section of the alloy JZ5 is shown in the Figure 4c,d. The thickness of the oxide scale was at about 3 μm, similar to that of the alloy JZ4. The EDS data of the scale and the phases in bulk is given in the Table S4 in the Supplementary Materials. The phases in the bulk were Nb_5_Si_3_, A15, TM_5_Sn_2_X and Cr_2_Nb Laves. The mixed oxide that formed the oxide scale was rich in Nb, Ti and Si. There was no significant change of the compositions of the phases in the bulk compared with JZ5-AC. There was no evidence of internal oxidation and no contamination of the phases by oxygen.

#### 4.2.2. Oxidation at 1200 °C

**Figure 5:**
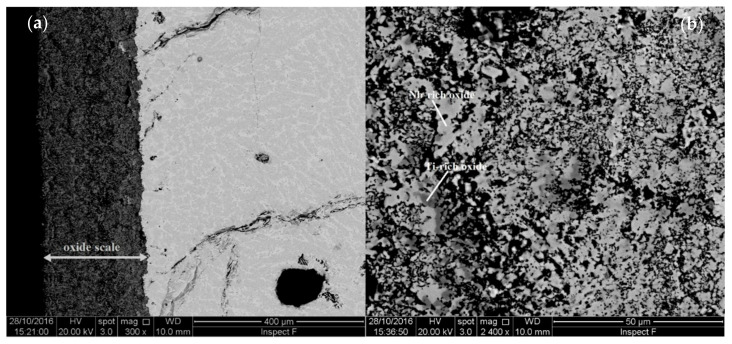

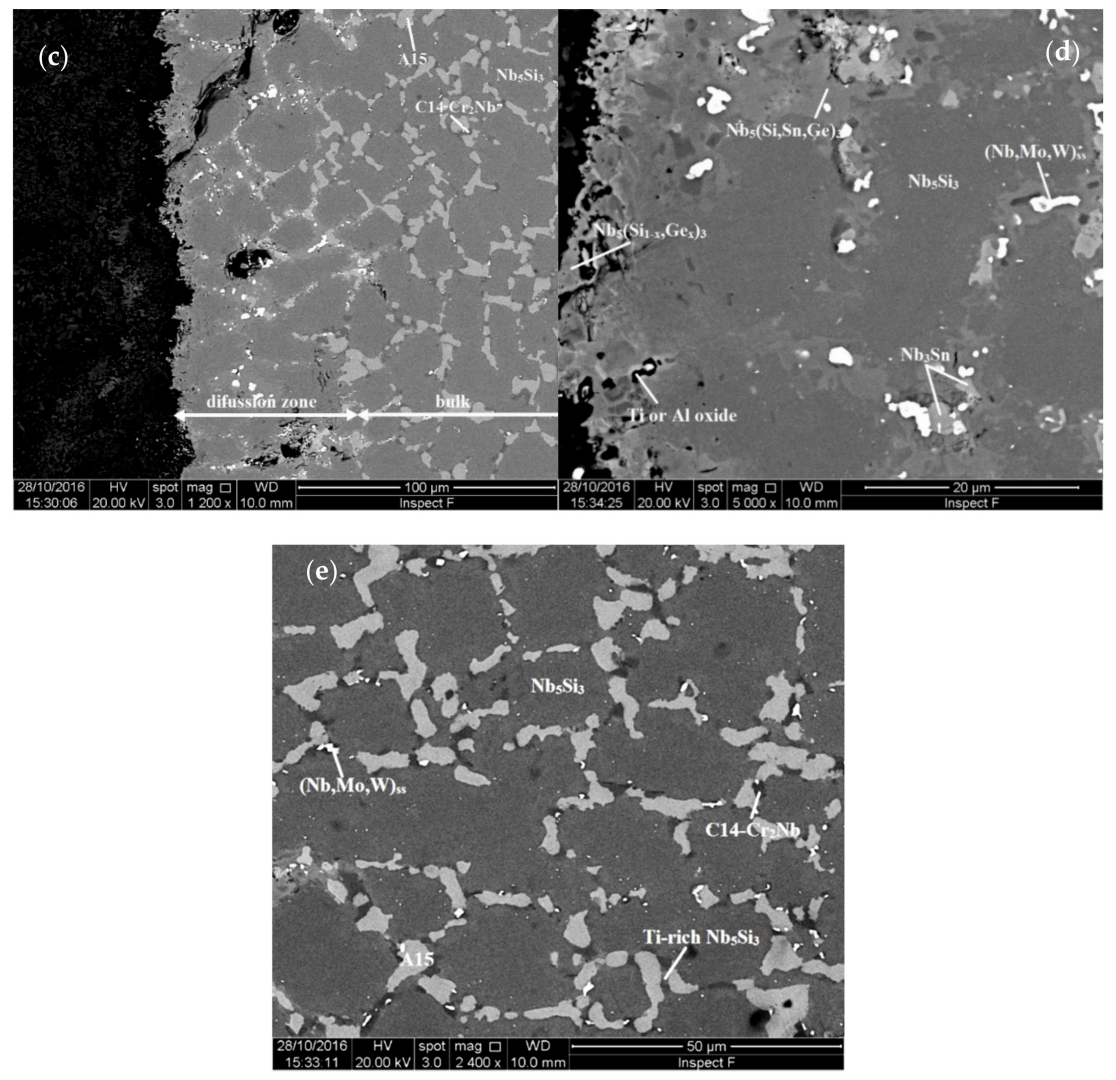
BSE images of the microstructure of a cross section of the oxidised alloy JZ4 at 1200 °C, (**a**) oxide scale and microstructure below it, (**b**) oxide scale, (**c**) diffusion zone and bulk, (**d**) microstructure of the diffusion zone and (**e**) bulk microstructure.

**Figure 6:**
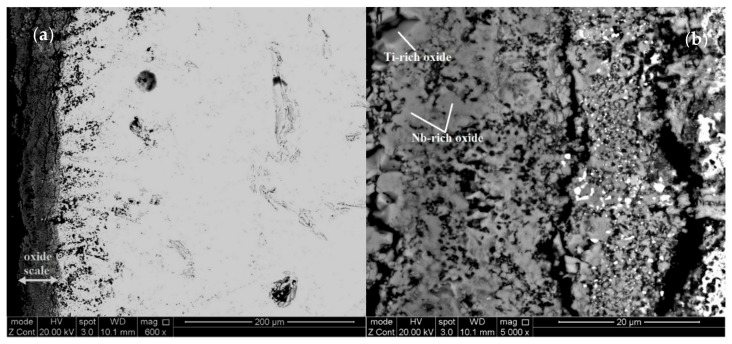

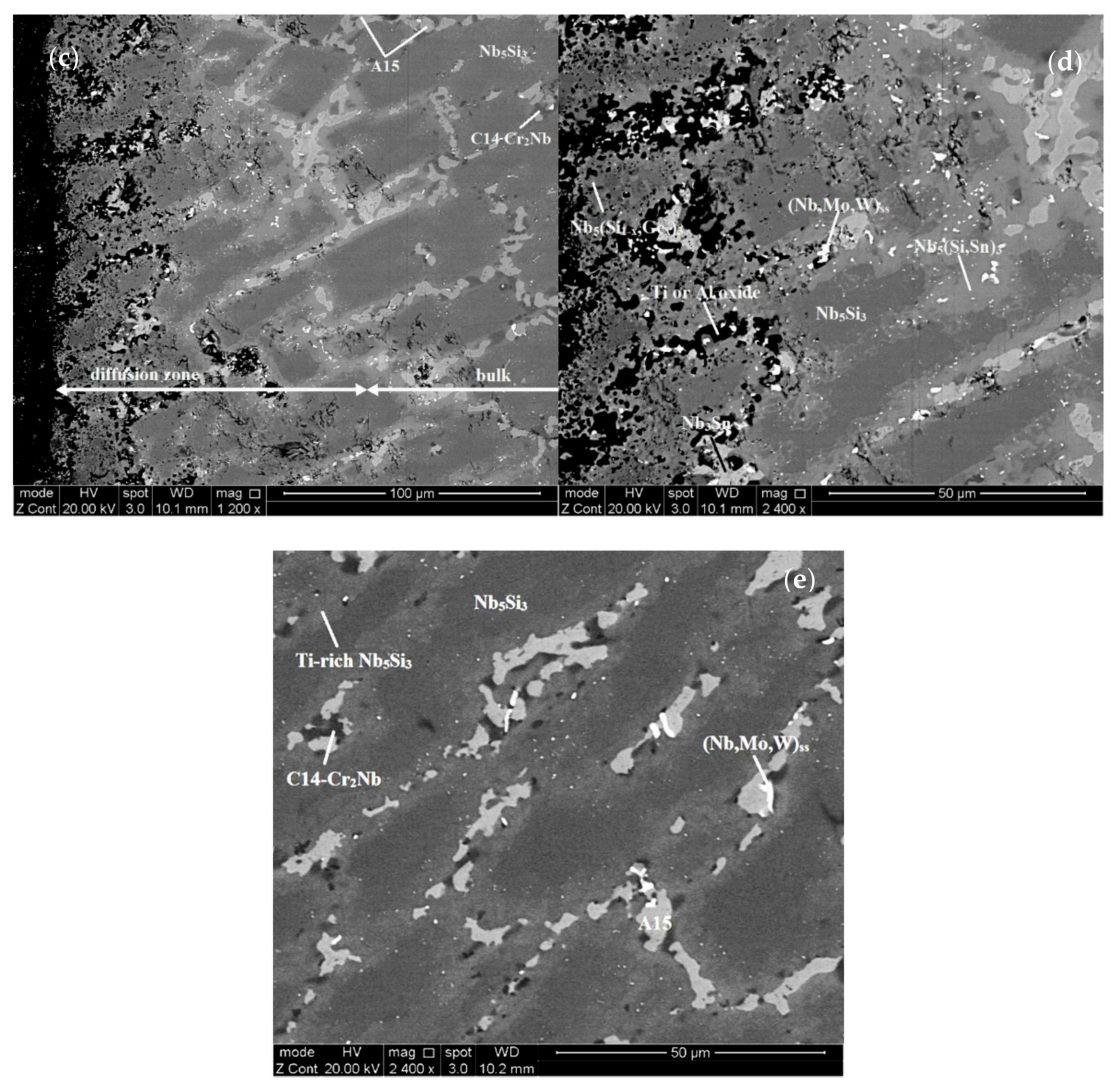
BSE images of the microstructure of a cross section of the oxidised alloy JZ5 at 1200 °C, (**a**) oxide scale, diffusion zone and bulk, (**b**) oxide scale, (**c**) diffusion zone and bulk, (**d**) microstructure of the diffusion zone and (**e**) bulk microstructure.

Figure 5 and Figure 6 show the microstructures of cross sections of the oxidised specimens of the alloys JZ4 and JZ5. For each alloy, three regions can be seen, namely the oxide scale, diffusion zone and bulk. The oxide scale that formed on the alloy JZ5 was significantly thinner compared with the alloy JZ4, about 50 and 200 µm, respectively. Cracks and holes were observed in the scales of both alloys.

The oxide scale of the alloy JZ4 consisted of three types of oxides, namely Nb-rich, Ti-rich (Table S5 in the Supplementary Materials) and Si-rich oxide. The latter exhibited black contrast, the same as the pores and cracks in the scale. Its concentration is not given in the Table S5. The concentrations of Sn and Ge in the scale were effectively zero. The Nb-rich oxide contained no Mo and the Ti-rich oxide contained no W. The latter oxide was richer in Al and Cr compared with the Nb-rich oxide.

**Figure 7:**
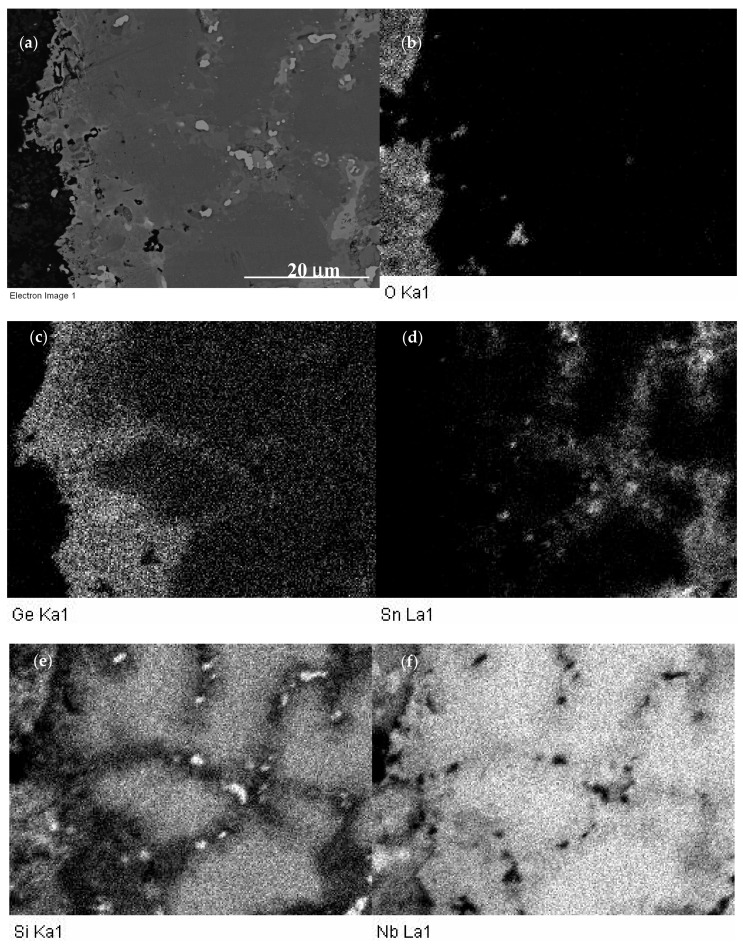

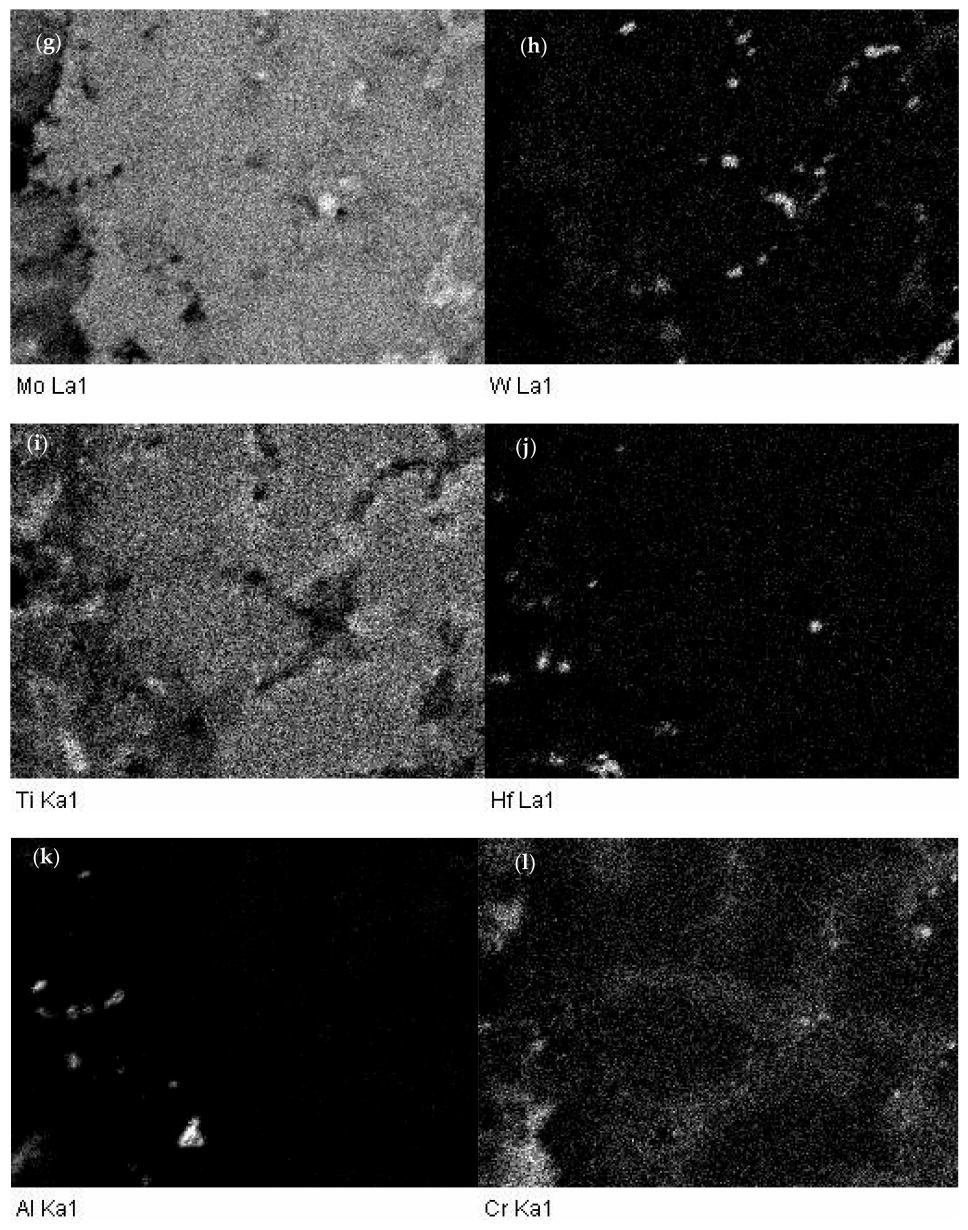
X-ray maps of a cross section of the oxidized specimen of JZ4 at 1200 °C, where: (**b**) Oxygen; (**c**) Ge; (**d**) Sn; (**e**) Si; (**f**) Nb; (**g**) Mo; (**h**) W; (**i**) Ti; (**j**) Hf; (**k**) Al; (**l**) Cr.

The phases in the diffusion zone of JZ4 were the Nb_5_Si_3_, Nb_5_(Si,Ge)_3_, Ge-rich Nb_5_(Si,Ge)_3_, Nb_3_Sn, (Nb,W)_ss_ and HfO_2_ (Table S5). The Ti and Al contents in these phases remained relatively high owing to the absence of Ti and/or Al oxides in the diffusion zone. These oxides were only observed in a few areas just below the scale. Compared with JZ4-HT, the (Nb,W)_ss_ in the diffusion zone was richer in Ge, Mo, W and poorer in Al, Cr, Ti. Compared with the solid solution in the bulk of the oxidised specimen, the (Nb,W)_ss_ in the diffusion zone was poorer in Ti and richer in Cr, Mo, W. The bulk microstructure of the oxidised specimen was similar to that of the cast alloy and included the solid solution, which was richer in Mo, W, Al and poorer in Cr, compared with the solid solution in JZ4-HT. The concentration of Mo in the A15 was high (21.9 at.%). The Nb_5_Si_3_ and Ti-rich Nb_5_Si_3_ were poorer in Si and the latter was also poorer in W and richer in Ti and Sn compared with the cast alloy. The Laves phase was richer in Si and poorer in Al and Ti. The TM_5_Sn_2_X was not observed in the bulk after the oxidation. X-ray maps corresponding to the scale and the diffusion zone below it are shown in the Figure 7. Chromium and Ti-rich areas in the scale, corresponding to the Ti-rich oxide, can be seen in the Cr and Ti maps (Figure 7i,l). Immediately below the scale the substrate was rich in Ge (Figure 7c). Tin-rich areas can be observed below the Ge-rich area (Figure 7c,d). The Sn-rich areas, some of which were also Ge-rich, corresponded with the areas in-between Nb_5_Si_3_ grains where the A15 and the solid solution were formed (Figure 5d).

The oxides in the scale that formed on the alloy JZ5 were similar to those of the alloy JZ4 (see Table S6 in the Supplementary Materials). A solid solution rich in Mo and W was present in the inner regions of the scale, where it exhibited white contrast, see Figure 6b. The phases in the diffusion zone that formed in JZ5 were the same as those in the alloy JZ4 and the vol.% of the Ti and Al oxides was slightly increased compared with the alloy JZ4. Compared with JZ5-HT, the (Nb,W)_ss_ in the diffusion zone was richer in Mo, W and poorer in Ti. The solid solution was also present in the bulk of the oxidised specimen but not the TM_5_Sn_2_X compound. The solid solution in the bulk of the oxidised specimen was richer in Mo, W and poorer in Al, Cr, Ti compared with JZ5-HT. The Ti-rich Nb_5_Si_3_ was richer in Ti and Sn and poorer in Si and W compared with the cast alloy. The A15 phase was richer in Mo and the Laves phase was poorer in Ti.

## 5. Discussion

### 5.1. Density

The densities of both alloys (Table 1) were (a) lower than those of the alloy JZ3+, which had the lowest density of the Ta-containing JZ series of alloys [46,47], (b) lower than the densities of Ti-free creep resistant Nb-silicide-based alloys (see Figures 2 and 4 in [1]), and (c) significantly lower than the target density (ρ = 9 g/cm^3^) of Nb-silicide-based alloys with RM additions and a strength of 450 MPa at 1500 °C [55].

The higher Ti concentration in the alloy JZ5 resulted in its density falling below 7 g/cm^3^ (Table 1). Furthermore, the density of JZ5 was lower than the density of some of the Ti-rich Nb-silicide-based alloys studied to date (Figure 5 in [1], and Table 4). The densities of the alloys JZ4 and JZ5 also satisfied the constraint (iii) in alloy design (Section 2). The data in Table 4 shows that with the exception of the RCCA alloy JZ3, the densities of all the other Nb-silicide-based alloys, which are also RCCAs, were lower than the lower density value (7.78 g/cm^3^) of multiphase RCCAs with bcc solid solution + M_5_Si_3_ silicide(s) in their microstructure, as reported in Table 2 in [2].

### 5.2. Macrosegregation

Macrosegregation of Si (MACSi) existed in both alloys, and was more severe in the alloy JZ5. MACSi has been defined as the difference between the maximum and minimum concentrations of Si in the alloy, i.e., MACSi = C_max_^Si^ − C_min_^Si^ [60]. Tin and Ge have a stronger effect on the macrosegregation of Si compared with Mo and W (Figure A1 in Appendix A), in particular Sn. MACSi increases when the parameters ΔH_m_/T_m_ (“alloy entropy of fusion”), T_m_^sp^ (melting temperature of sp electronic configuration elements) and (ΔH_m_/T_m_)/(ΔH_m_^sd^/ΔH_m_^sp^) increase and the ratios ΔH_m_^sd^/ΔH_m_^sp^ and T_m_^sd^/T_m_^sp^ and the parameters ΔH_m_ (“alloy enthalpy of melting”), T_m_ (alloy melting temperature) and T_m_^sd^ (melting temperature of the sd electronic configuration elements) decrease [60].

Data for MACSi in Ti-rich Nb-silicide-based alloys with/out Ge, Mo or Sn are compared with the alloy JZ5 in Table 5. The aforementioned trends are followed by T_m_ (with the exception of the alloy KZ5), ΔH_m_/T_m_, ΔH_m_^sd^/ΔH_m_^sp^, T_m_^sd^, T_m_^sp^, T_m_^sd^/T_m_^sp^ and (ΔH_m_/T_m_)/(ΔH_m_^sd^/ΔH_m_^sp^). The alloy JZ4 had MACSi = 3 at.%, slightly lower than that of JZ3+ [47]. The MACSi values calculated by NICE were 3.7 and 5 at.%, respectively, for JZ4 and JZ5. The data for the alloys JZ1, JZ2 [46], JZ3, JZ3+ [47], JZ4 and JZ5 show that both experimental and calculated MACSi increased with decreasing Sn/Ge and Al + Cr (figures not shown). Two noteworthy results of this research are (1) that the substitution of Ta by Mo in JZ4 not only improved oxidation resistance and reduced density but also reduced MACSi and (2) that MACSi can be controlled with solute additions that play a key role in the oxidation resistance of these alloys, namely Al, Cr, Ge and Sn. The MACSi increased with the increase in the concentration of Ti in the alloy JZ5, but was lower than that of the oxidation-resistant alloy OHS1 [45].

**Figure 8:**
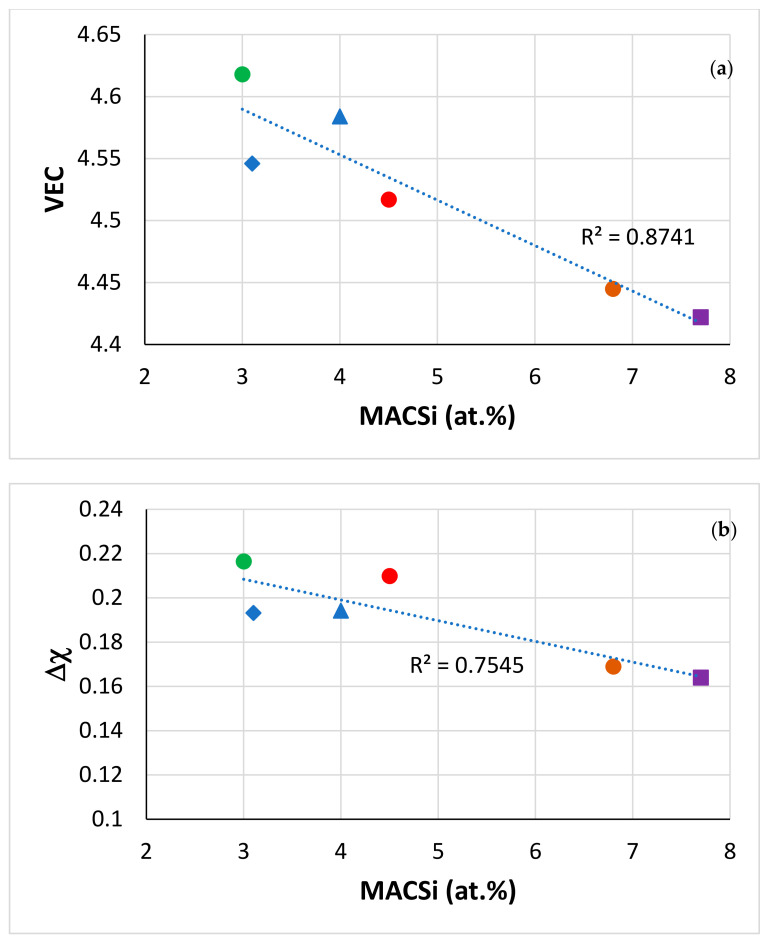
(**a**) VEC versus macrosegregation of Si (MACSi) and (**b**) Δχ versus MACSi of RCCAs studied in our research group. Data are as follows: green alloy JZ4, red JZ5, orange OHS1, square EZ8, triangle JZ3, diamond JZ3+, for nominal compositions of alloys see the Abbreviations. If the data for JZ3+ were to be excluded (for the reasons discussed in [47]) in (**a**) R^2^ = 0.9537 and in (**b**) R^2^ = 0.8901.

There are no data in the literature about macrosegregation of solute elements in RCCAs that were cast using arc melting or other cold hearth processes [1,2], with the exception of the RCCAs studied in our group [45,47,56,58,59]. Figure 8 shows that in RCCAs, the MACSi decreases as the alloy parameters VEC or Δχ increase (the correlation between MACSi and the alloy parameter δ is poor, figure not shown). In the Figure 8 it should be noted (i) that the solid solution was not stable in the alloys OHS1 and EZ8, (ii) that in all the alloys the Nb_5_Si_3_, A15 and C14-Cr_2_Nb were stable and (iii) that the solid solution was stable in the Ta or Mo containing alloys, respectively JZ3, JZ3+ [47] and JZ4, JZ5 (Table 4). It should also be noted that in the microstructures of the other RCCAs studied to date [2], stable phases were (iv) the solid solution with M_5_Si_3_ silicide, or (v) the solid solution with Laves phase, or (vi) the solid solution, with M_5_Si_3_ and Laves and (vii) that all the RCCAs did not contain A15 compound(s) [2]. On first sight it might seem strange that MACSi, which is linked with solidification conditions and solute partitioning [1,41,42,43,60,62], on which depend the chemical compositions of the phases in the as-cast microstructures, correlates with the aforementioned parameters. However, the study of the alloying behaviour of Nb-silicide-based alloys and their phases has shown (a) that the concentrations of solute elements in the Nb_ss_, Nb_5_Si_3_, A15-Nb_3_X and C14-Cr_2_Nb correlate well with the phase parameters VEC and Δχ [21,63,64] and (b) that the phase parameters correlate well with the alloy parameters, for example Δχ_alloy_ = h(Δχ_Nb5Si3_) [20]. It is pointed out that the trends shown in the Figure 8 should be used with caution because the data about MACSi in RCCAs are limited.

### 5.3. Microstructures

The actual compositions of both alloys were close to the selected compositions. Remarkably, in both alloys the Al/Cr and Sn/Ge ratios were the same as in OHS1, JZ3 and JZ3+, and in the alloy JZ4 the Mo/W and Ti/Hf ratios were similar to those in JZ3 and JZ3+ [45,46,47].

In both alloys, the “architecture” of the cast microstructure was the same. Furthermore, the microstructure of the alloy JZ5 was slightly coarser. The solid solution was not observed in the as-cast buttons of both alloys but was stable after the heat treatment. In the interdendritic areas of the primary Nb_5_Si_3_, the A15-Nb_3_X, C14-Cr_2_Nb and TM_5_Sn_2_X were formed. Note that the latter compound was not observed in the Ta-containing JZ series of alloys [46,47]. The primary Nb_5_Si_3_ was not facetted, in agreement with [6,59]. It is concluded that the substitution of Ta by Mo did not destabilise the A15 and Laves phases, decreased the vol.% of the A15, particularly in the alloy JZ4 compared with JZ3, and unlike the Ta-containing alloys JZ3 and JZ3+ [47], suppressed the solid solution in the cast microstructure and promoted the formation of TM_5_Sn_2_X in both JZ4 and JZ5. Moreover, in JZ5-AC, the increase in the Ti concentration suppressed the sensitivity of the formation of TM_5_Sn_2_X on cooling rate and increased the volume fraction of this compound, which would suggest that Ti “boosted” its formation.

The TM_5_Sn_2_X compound in both alloys is based on the Nb_5_Sn_2_Si, Ti_5_Sn_2_Si, Nb_5_Sn_2_Ge, Nb_5_Sn_2_Al compounds that all have the tetragonal W_5_Si_3_ as prototype [54,65]. The Nb_5_Sn_2_Si compound is stable at 900 °C and 1200 °C according to Sun et al. [66]. Bulanova et al. [67] reported the Ti_5_Si_1.2–1.6_Sn_1.8–1.4_ compound in Ti-Si-Sn alloys. With the increase in the Ti concentration in JZ5 the Ti content of TM_5_Sn_2_X also increased, as did the Ti/Nb ratio and the Al + Ge + Si content (1.17 and 12.1 at.%, and 2.96 and 14.4 at.%, respectively, in JZ4 and JZ5). The Sn content was essentially the same in both alloys (about 24.8 at.%). It should be noted (a) that the oxidation resistance of Ti-rich Nb-silicide-based alloys was improved with Ti and Mo addition [68] and was enhanced further with the addition of Hf and Sn [44,56], and (b) that this enhancement was associated with the formation of the TM_5_Sn_2_X compound at 1200 °C in the substrate just below the scale/substrate interface that has been observed in many Sn containing alloys [41,42,45,56].

In both alloys, as the primary Nb_5_Si_3_ formed, the surrounding melt became rich in Ti, Mo, W, Sn, Al and Cr. Considering the cast microstructures, in which the TM_5_Sn_2_X and C14-Cr_2_Nb Laves formed with small volume fractions, and that the phase with the highest melting temperature should form first, in the melt surrounding the Nb_5_Si_3_ the A15-Nb_3_X phase formed first followed by the C14-Cr_2_Nb and then the TM_5_Sn_2_X. It is suggested that in both alloys the solidification path was L → L + βNb_5_Si_3_ → L + βNb_5_Si_3_ + A15-Nb_3_X → L + βNb_5_Si_3_ + A15-Nb_3_X + C14-Cr_2_Nb → βNb_5_Si_3_ + A15-Nb_3_X + C14-Cr_2_Nb + TM_5_Sn_2_X. The presence of αNb_5_Si_3_ in the cast microstructures was attributed to the βNb_5_Si_3_ → αNb_5_Si_3_ transformation during solid-state cooling.

The microstructures of both the heat-treated alloys consisted of the Nb_5_Si_3_, A15-Nb_3_X, and Mo and W-rich solid solution (Nb,W)_ss_, and a very small vol.% of the C14-Cr_2_Nb Laves phase only in JZ4-HT. The TM_5_Sn_2_X was not stable at 1500 °C in both the alloys. The solid solutions were free of Si owing to the addition of Mo and W, which promote the formation of the Si-free Nb_ss_ [53]. It is suggested that the Nb_5_Si_3_ silicide, A15-Nb_3_X and the (Nb,W)_ss_ were the stable phases in the microstructures of JZ4 and JZ5 at 1500 °C, with the C14-Cr_2_Nb Laves phase maybe stable only in the alloy JZ4.

Both the β and αNb_5_Si_3_ were present in the heat-treated alloys JZ4 and JZ5 (Figures S1 and S2). Molybdenum and W form, respectively, the Mo_5_Si_3_ and W_5_Si_3_, which have the D8_m_ structure and are isomorphous with the βNb_5_Si_3_, but, unlike Ta, do not form silicides isomorphous with αNb_5_Si_3_. In the base alloy Nb-24Ti-18Si-5Al-5Cr (alloy KZ5 in [61]), the βNb_5_Si_3_ formed in the cast microstructure and both βNb_5_Si_3_ and αNb_5_Si_3_ were present after the heat treatment. The same was the case when Mo was added in the alloy Nb-18Si-5Al-5Cr-5Mo (alloy JG1 in [52]). The addition of Sn to the base alloy KZ5 promoted the βNb_5_Si_3_ → αNb_5_Si_3_ transformation upon heat treatment (alloys ZX7 and ZX8 in [41,42]) (meaning the βNb_5_Si_3_ that formed in the cast alloy transformed to αNb_5_Si_3_ after the heat treatment) but this transformation became sluggish when the base alloy KZ5 was alloyed with Ge with or without Hf (alloys ZF6 and ZF9 in [59]) (meaning that after the heat treatment both the βNb_5_Si_3_ and αNb_5_Si_3_ were present in the microstructure, as was the case in KZ5), whereas the simultaneous addition of Ge and Sn in the base alloy KZ5 stabilised the βNb_5_Si_3_ in the alloy OHS1 (meaning the βNb_5_Si_3_ → αNb_5_Si_3_ transformation did not occur upon the heat treatment [45]). Furthermore, the βNb_5_Si_3_ → αNb_5_Si_3_ transformation occurred in the case of the Al, Cr, Ge and Sn free and Ti poor alloy Nb-8.3Ti-21.1Si-5.4Mo-4W-0.7Hf buttons and suction cast bars (alloy CM1 in [62]) upon heat treatment. It is therefore likely that, owing to the simultaneous addition of Ge and Sn with Mo, W, Al and Cr, the stability of βNb_5_Si_3_ was increased in JZ4 and JZ5 compared with JZ3 and JZ3+. Could the solubility of Mo in Nb_5_Si_3_ affect the aforementioned transformation?

In as-cast and heat-treated Ti free Nb-xMo-36Si and Nb-xMo-37.5Si (x = 0 to 10 at.%) alloys that were prepared using arc melting, Sekido et al. [69] reported (a) Mo concentrations of 5.2 at.% and 3.6 at.% in βNb_5_Si_3_ and αNb_5_Si_3_, respectively, (b) that Mo stabilised the βNb_5_Si_3_, (c) that the βNb_5_Si_3_ → αNb_5_Si_3_ transformation did not occur when the concentration of Mo in Nb_5_Si_3_ exceeded 6 at.% and (d) that after heat treatment at 1400 °C for 100 h a mixture of both βNb_5_Si_3_ and αNb_5_Si_3_ were formed when the Mo concentration in the Nb_5_Si_3_ was 4 or 5 at.%. Concentrations of 2.5 at.% Mo and 0.6 at.% Mo were reported, respectively, in βNb_5_Si_3_ and αNb_5_Si_3_ for the cast and heat-treated conditions of the alloy Nb-18Si-5Al-5Cr-5Mo and slightly lower Mo concentration (1.9 at.%) in the βNb_5_Si_3_ in the cast alloy Nb-24Ti-18Si-5Al-5Cr-5Mo [52]. The above data suggest that the solubility of Mo (i) is different in βNb_5_Si_3_ and αNb_5_Si_3_, (ii) depends on the presence or not of Ti in the alloy and (iii) depends on the concentration of Mo in the alloy. In the alloys JZ4 and JZ5, where both βNb_5_Si_3_ and αNb_5_Si_3_ were present in the cast and heat-treated conditions, the average concentration of Mo in Nb_5_Si_3_ was similar to (d), namely 4.5 and 4.9 at.%, and 4.3 and 5 at.%, respectively, in the cast and heat-treated alloys JZ4 and JZ5 (Tables S1 and S2).

The solubility of W in Nb_5_Si_3_ was in the range 1 to 1.7 at.% in both the as-cast alloys, and slightly lower in JZ5-AC, and was ≤0.6 at.% after the heat treatment in both alloys. The W solubility in αNb_5_Si_3_ was reported to be about 1 at.% in Nb-W-Si alloys [70] and ≤0.6 at.% in the alloys Nb-20Si-5Hf-5W, Nb-20Si-5Mo-3W and Nb-20Si-5Hf-5Mo-3W (respectively, alloys YG5, YG6 and YG8 in [53]). Thus, the data suggest that the solubility of W in Nb_5_Si_3_ does not depend strongly on the alloying additions of Al, Cr, Ge, Mo, Sn and Ti, when the latter elements are present simultaneously in Nb-silicide-based alloys.

In both alloys there was precipitation of solid solution in the Nb_5_Si_3_ after the heat treatment (Figure 1e,f). Precipitation of Nb_ss_ in αNb_5_Si_3_ grains has been reported after the heat treatment of the alloys Nb-24Ti-18Si-5Al (alloy KZ7 in [61]), KZ5 [61], JG1 [56], Nb-24Ti-18Si-5Ge-5Al (alloy ZF5 in [71]), the arc melted button and suction cast bars of the alloy CM1 [62] after heat treatment, the large arc melted ingots of the alloy CM1 before and after heat treatment [62] and the as-cast and heat-treated OFZ bars of the alloy CM1 grown at three different growth rates [62]. In Nb-24Ti-18Si-8Cr-4Al (alloy KZ2 in [72]) only the βNb_5_Si_3_ was present in the cast microstructure and both the βNb_5_Si_3_ and αNb_5_Si_3_ after the heat treatment with Nb_ss_ precipitates in Nb_5_Si_3_ grains. For the Ti-rich Nb-silicide-based alloys Zelenitsas and Tsakiropoulos attributed this precipitation of the Nb_ss_ to the βNb_5_Si_3_ → αNb_5_Si_3_ + Nb_ss_ transformation [61].

For the Nb-xMo-36Si and Nb-xMo-37.5Si (x = 0 to 10 at.%) alloys (see above), Sekido et al. [69] reported (i) that Nb_ss_ precipitates formed in both βNb_5_Si_3_ and αNb_5_Si_3_ after heat treatment at 1300 °C for 20 h, (ii) that precipitation of Nb_ss_ in βNb_5_Si_3_ was not observed in the cast condition but after heat treatment at 1500 °C for 100 h, and (iii) that in the αNb_5_Si_3_ that formed from the βNb_5_Si_3_ → αNb_5_Si_3_ transformation after heat treatment at 1500 °C for 100 h there was Nb_ss_ precipitation in αNb_5_Si_3_. For the Nb_ss_ precipitates, two orientation relationships were observed by Sekido et al., which were in agreement with the orientation relationships reported for eutectoid Nb_ss_/αNb_5_Si_3_ lamellae by Sekido et al. [73] and Miura et al. [74]. Sekido et al. suggested that the βNb_5_Si_3_ → αNb_5_Si_3_ transformation occurred either before the Nb_ss_ precipitated or simultaneously [69]. The results for the alloys KZ7, KZ5, JG1, ZF5 and CM1 (see the Abbreviations for the nominal compositions) are consistent with the precipitation of Nb_ss_ in αNb_5_Si_3_, whereas according to the results of Sekida et al. precipitation of Nb_ss_ is also possible in βNb_5_Si_3_. It should be noted that precipitation of Nb_ss_ was also observed in Nb_5_Si_3_ grains in JZ3-HT and [JZ3+]−HT [47] as well as in Nb_5_Si_3_ grains in the heat-treated alloys Nb-24Ti-18Si-6Ta-5Al-5Cr and Nb-24Ti-18Si-6Ta-8Cr-4Al (respectively, alloys KZ6 and KZ8 in [72]), which would suggest that Ta, similarly to Mo, promotes the aforementioned phenomenon.

The hardness of the as-cast alloys JZ4 and JZ5 was 862 HV and 838 HV, which gives room temperature strengths of 2816 MPa and 2737 MPa, and specific room temperature strengths of 387 and 396 MPa cm^3^g^−1^, respectively. After the heat treatment the room temperature strength was 2724 MPa and 2633 MPa, respectively for JZ4 and JZ5. Both the room temperature strength and specific strength were higher than other RCCAs [1,2] and comparable with those of boron containing Nb-silicide-based alloys [1].

The (Nb,Ti,Hf)_3_(Si,Sn,Al) and (Nb,Ti,Cr,Hf)_3_(Si,Sn,Al) A15 compounds have more than double the hardness of Nb_3_Sn (450 HV) [21]. The alloys JZ4 and JZ5 had lower vol.% A15 compared with JZ3 and JZ3+ [47], particularly the alloy JZ4, and the alloy JZ5 had lower vol.% Nb_ss_ than JZ3+. The hardness of the solid solution was the same in both alloys (850 HV) but in the alloy JZ5 the contribution to hardness of Al, Cr and Ti was 58% higher compared with JZ4 (372 HV and 236 HV, respectively (calculations used data for binary Nb-X (X = Al, Cr, Ti) from [27,31]), in other words, the smaller contribution of Mo and W to the hardness of the solid solution, owing to the lower Mo + W concentration in the (Nb,W)_ss_ in JZ5-HT, was compensated by the higher concentrations of Al, Cr and Ti. The hardness of the Nb_5_Si_3_ was higher than that of the binary silicide [63]. In spite of the fact that the microstructures of both alloys consisted of phases that had high hardness, microcracking was not observed in the as-cast buttons and after specimen preparation. The βNb_5_Si_3_ has lower hardness and Young’s modulus and higher CTE anisotropy than αNb_5_Si_3_ and alloying with Mo reduces the CTE anisotropy [63]. To the authors’ knowledge there are no data about the effect of Ta on the CTE anisotropy of Nb_5_Si_3_. Suppression/elimination of microcracking after solidification and specimen preparation would benefit from lower CTE anisotropy, and the lower hardness of the Nb_5_Si_3_ in JZ4 and JZ5 compared with JZ3+ [47]. Precipitation of Nb_ss_ in Nb_5_Si_3_ significantly improved the toughness of the silicide in both JZ4-HT and JZ5-HT (results not shown).

**Figure 9:**
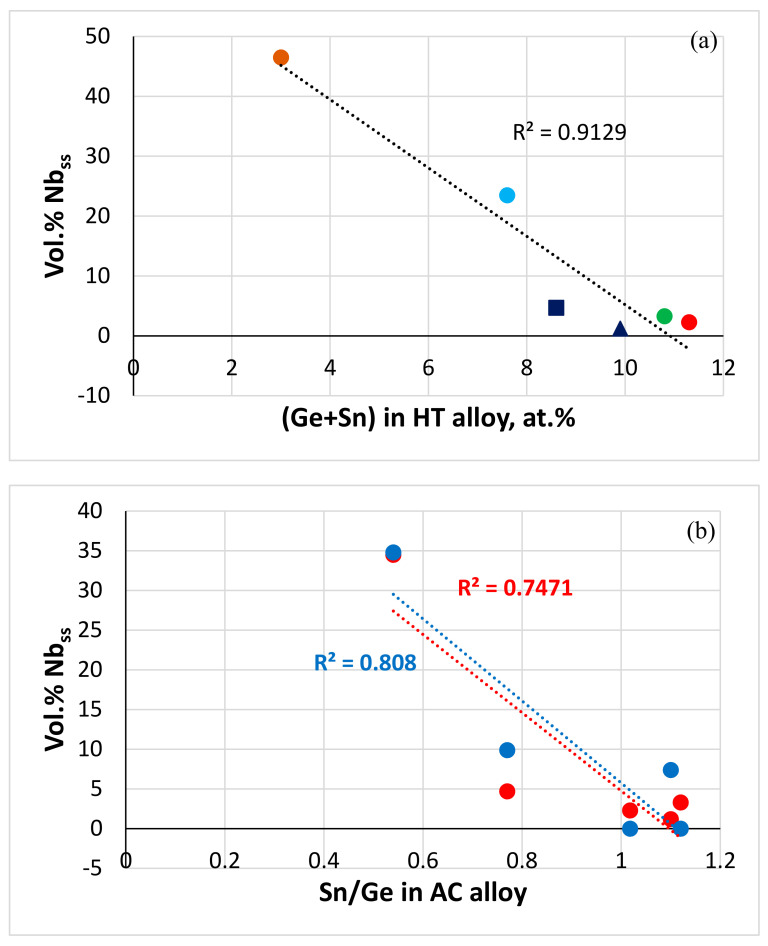
Volume fraction Nb_ss_ as function (**a**) of Ge + Sn in heat-treated alloys and (**b**) of Sn/Ge in as-cast alloys. In (**a**) data for HT alloys, JZ1, JZ2 [46], JZ3, JZ3+ [47], with red for JZ5, green for JZ4, triangle for JZ3+, square for JZ3, blue for JZ2, and brown for JZ1. All data R^2^ = 0.9129, and data for Ta-containing alloys R^2^ = 0.942. In (**b**) data for cast alloys JZ2 to JZ5, red filled circles for experimental data, and blue circles for calculated data using NICE. AC = as cast, HT = heat treated.

NICE [20] correctly predicted the vol.% Nb_ss_ in JZ4-AC and JZ5-AC (zero in both alloys) (Table 1) as well as the dependence of the vol.% Nb_ss_ on the Sn/Ge ratio in the cast alloys JZ2, JZ3, JZ3+, JZ4 and JZ5, although the R^2^ values for linear fit of data are less than 0.9 (Figure 9b). The vol.% Nb_ss_ decreased with increasing Sn/Ge ratio and Sn + Ge sum in the alloys JZ2 to JZ5 (Figure 9).

The compositions of the solid solutions in the alloys JZ4 and JZ5 that were calculated by NICE [20], respectively, were 36Nb-6.4Ti-0Si-20.2Mo-21.2W-0.7Sn-0.4Ge-0.8Hf-3.6Al-10.7Cr and 24.7Nb-10.5Ti-0Si-26.3Mo-18.8W-1.4Sn-0.6Ge-0.8Hf-4Al-12.9Cr. Compared with the experimental data (Tables S1 and S2), NICE underestimated the W and Cr, and overestimated the Nb content in the Nb_ss_ of JZ4 and underestimated the Cr and Ge, and overestimated the Nb and Mo contents in the Nb_ss_ of JZ5. Bearing in mind the accuracy of chemical analysis data and the luck of thermodynamic data for ternary systems that are relevant to the studied alloys [1,20], we consider the agreement between experimental and calculated Nb_ss_ to be good.

The parameters VEC, Δχ and δ of the Nb-silicide-based alloys and their solid solutions [7,50] are key for the calculations in NICE [20]. The experimental and calculated VEC, Δχ and δ parameters of the Nb_ss_ in JZ4, respectively, were 5.499, 0.337, 4.574 and 5.366, 0.33, 4.596, and of the Nb_ss_ in JZ5, respectively, were 5.341, 0.321, 5.463 and 5.367, 0.327, 5.09. Compared with the data for the Nb_ss_ in Nb-silicide-based alloys in [7], the data for the experimental compositions of the solid solutions show that the range of VEC is expanded from 4.4–5.4 to 4.4–5.5. The data for the alloy JZ3+ [47] showed that the range of Δχ also changed from 0.039–0.331 to 0.039–0.369. The Δχ values of the Nb_ss_ in JZ4 and JZ5 are in the latter range. In [1,7,20] it was discussed that Si-free Nb_ss_ in Nb-silicide-based alloys has δ less than approximately 5. This is supported by the results of this study.

The chemical composition of the Nb_5_Si_3_ silicide calculated by NICE [20] was 50.8(Nb, Mo, W)-9.4Ti-27.2Si-1.6Sn-5.9Ge-1.4Hf-1.9Al-1.8Cr and 44.9(Nb, Mo, W)-15.4Ti-26.5Si-1.6Sn-6.1Ge-1.9Hf-2.4Al-1.2Cr, respectively, in the cast alloys JZ4 and JZ5. Owing to the limited data about Mo and W in the NICE database, the total RM concentration was calculated instead of the concentration of each RM. Compared with the experimental data (Tables S1 and S2) NICE underestimated and overestimated, respectively, Si and Hf in the Nb_5_Si_3_ in JZ5. We consider the agreement between experimental and calculated data to be good.

The parameters VEC, Δχ, δ and the ratios sd/sp (sd electronic configuration elements over sp electronic configuration elements) and Nb/(Ti + Hf) of the alloys JZ4 and JZ5, respectively, were 4.618, 0.2165, 9.42, 1.96, 2.87 and 4.517, 0.2099, 9.66, 1.89, 1.5. The parameters VEC, Δχ and δ fall in the ranges of the parameters of Nb-silicide-based alloys [50]. NICE can calculate the creep rate due to intrinsic resistances to dislocation motion [1,20] and, together with calculations linked with oxidation behaviour, can point out whether a designed/selected alloy is worthy of further investigation after considering its creep and oxidation. The calculated creep rates at 1200 °C and 170 MPa using the above parameters and ratios were in the ranges 3 × 10^−6^ s^−1^ to 8.5 × 10^−10^ s^−1^, and 1.7 × 10^−4^ s^−1^ to 2.7 × 10^−9^ s^−1^, with average creep rates 4.5 × 10^−7^ s^−1^ and 2.7 × 10^−6^ s^−1^, respectively, for the alloys JZ4 and JZ5. The average creep rate of JZ4 was lower than that of JZ5, as would be expected when considering the Nb/(Ti + Hf) ratios [6]. The average creep rates were lower than the creep rate of the single crystal Ni-based superalloy CMSX-4 for the same conditions (5.6 × 10^−5^ s^−1^), but higher than the creep rate of 1 × 10^−7^ s^−1^ that is the criterion in NICE to decide whether it is likely for a designed/selected alloy to meet the creep goal [20]. However, it should be noted that the lowest values of the calculated creep rates for both alloys were lower than 1 × 10^−7^ s^−1^.

### 5.4. Oxidation

#### 5.4.1. Oxidation at 800 °C

The Figure 2 and Figure 3 showed (i) that the substitution of Ta by Mo resulted in significant improvement of oxidation at 800 °C in both alloys, as did the increase in the Ti concentration in the alloy JZ5, and (ii) confirmed the beneficial effect of both Mo and Ti on the oxidation resistance of Nb-silicide-based alloys that was reported in [68].

The alloys JZ4 and JZ5 exhibited the best oxidation resistance at 800 °C (Figure 2a), gained the lowest weight compared with the alloys JZ1 to JZ3+ [46,47], the oxidation specimens retained their shapes, did not pest (Figure 3a,b) and formed thin oxide scales. Their oxidation at 800 °C followed parabolic oxidation kinetics similar to the single crystal Ni superalloy CMSX-4 that gained 0.04 mg/cm^2^ after 50 h and had *k*_p_ = 4 × 10^−14^ g^2^ cm^−4^ s^−1^ [1].

Compared with the alloy JZ3 [47], the substitution of Ta by Mo in JZ4 changed the oxidation kinetics at 800 °C from linear to parabolic, decreased the weight change by 94.5% and eradicated cracks in the scale. Compared with the alloy JZ3+ the weight change decrease was similar (96%) at 800 °C and the pest oxidation was suppressed [47].

The effect of Mo with Al and Cr on the oxidation resistance at 800 °C of Nb-24Ti-18Si-based alloys without Sn and Hf additions was reported by Geng et al. [68], who showed that the addition of 2 at.% Mo was more beneficial than 5 at.%. Pest oxidation was eliminated in the Nb-24Ti-18-Si-5Al-5Cr-5Hf-5Sn-2Mo alloy with the addition of Hf and Sn. Comparison of the TG data of the JG series of alloys [56,68] and the alloys JZ1 and JZ2 [46], JZ3 and JZ3+ [47], JZ4 and JZ5 shows (i) that the beneficial effect of the synergy of Mo with Sn, Ge, Al, Cr, Ti and Hf and W was stronger than that of Ta with the same elements and (ii) that the detrimental effect of Mo addition at high concentration on the oxidation of Nb-25Ti-18Si-5Al-5Cr-based alloys was compensated with the addition of Sn and Ge with Hf, Ti and W. This is encouraging news because (a) Mo has a lower density and is a better solid solution strengthener than Ta [1,27,28,31] and (b) points to the synergy of Mo and W in Nb-silicide-based alloys and in RCCAs being more desirable than that of Ta and W for achieving a balance of strength and oxidation, and/or properties closer to the property goals [1].

**Figure 10:**
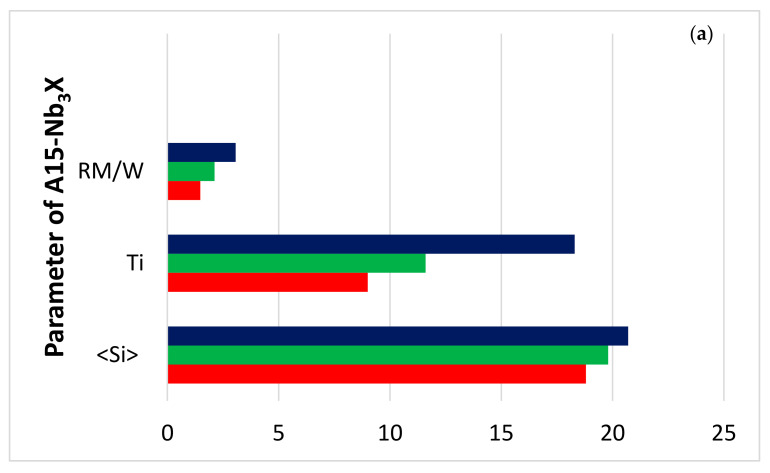

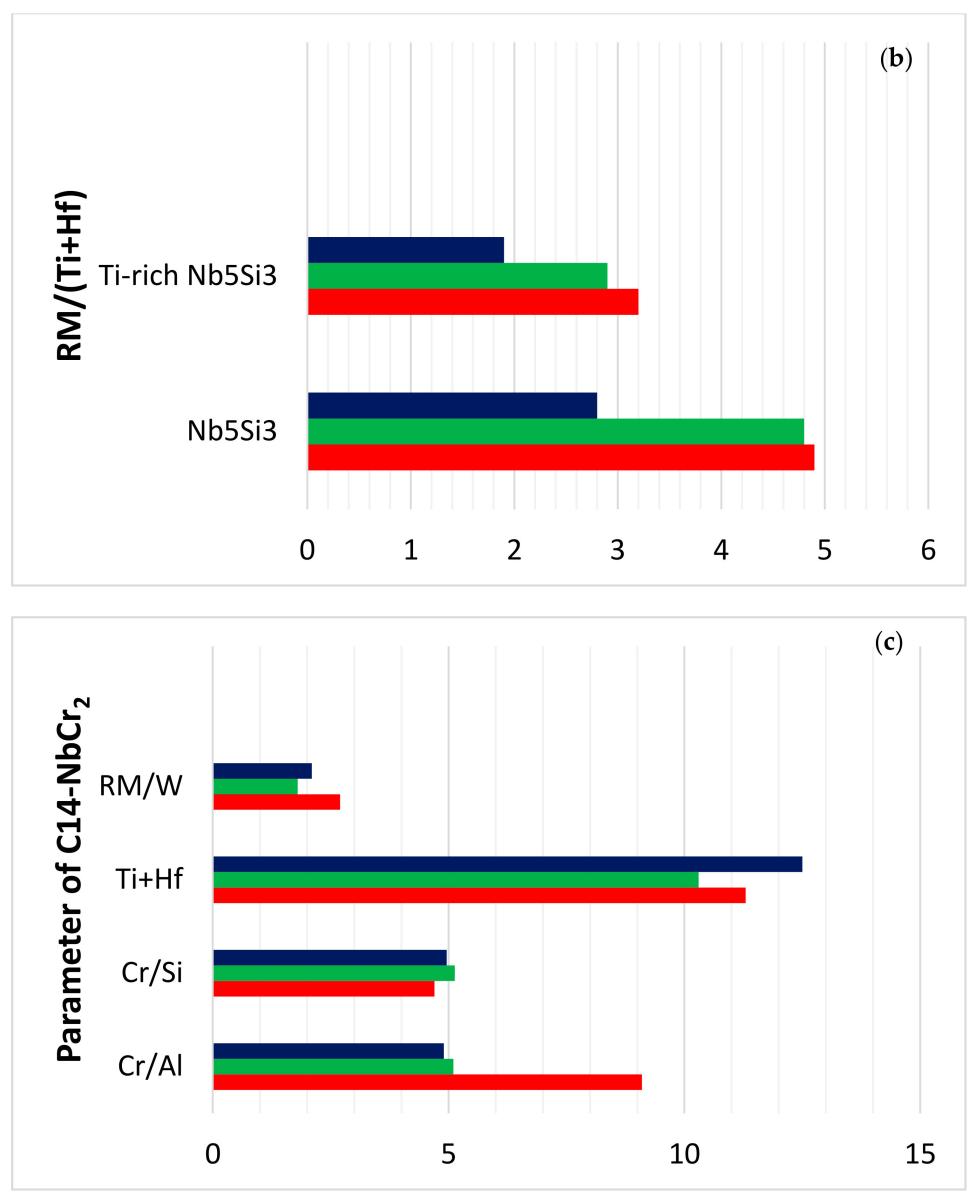
Parameters of different phases for the bulk of oxidised specimens at 800 °C, red JZ3, green JZ4, blue JZ5. (**a**) Data for A15-Nb_3_X, parameters <Si> (at.%), RM/W, concentration of Ti (at.%), (**b**) data for Nb_5_Si_3_, parameter RM/(Ti + Hf), (**c**) data for C14-NbCr_2_, parameters Cr/Al, Cr/Si, Ti + Hf, RM/W. RM = Mo or Ta, <Si> = Al + Ge + Si + Sn.

The starting microstructures of the oxidation specimens of the alloys JZ4 and JZ5 were slightly different compared with the alloys JZ3 and JZ3+, in that the TM_5_Sn_2_X compound was present and the volume fraction of the Laves phase was higher than those in the alloys JZ3 and JZ3+ [47]. The microcracking that was observed in JZ3+ was not noted in JZ4 and JZ5. There was no apparent change in the compositions of the phases in the alloys JZ4 and JZ5 after the oxidation, compared with the cast alloys. Considering the data for the composition of phases in the bulk of the alloys JZ3 [47], JZ4 and JZ5 after oxidation at 800 °C, (i) the parameters <Si>, RM/W and the Ti content of the A15 in JZ5 were the highest (Figure 10a), (ii) the RM/(Ti + Hf) ratio of the Nb_5_Si_3_ and Ti-rich Nb_5_Si_3_ were the lowest for JZ5 (Figure 10b) and (iii) the Laves phase was the poorest in Al and richest in Si in JZ3, and had the highest RM/W and Ti + Hf content, respectively, in JZ3 and JZ5 (Figure 10c). Considering that the Laves phase plays a minor role in the oxidation of Nb-silicide-based alloys at 800 °C [75] and that the TM_5_Sn_2_X compound was present only with a small volume fraction in JZ4 and JZ5, it is suggested that the good oxidation resistance of the alloys JZ4 and JZ5 at 800 °C is attributed to the low volume fraction of the A15, the absence of Nb_ss_ in the starting microstructure and the aforementioned parameters (Figure 10).

It should be noted that some of the A15 phase immediately below the oxide scale was oxidised in the alloy JZ4 (Figure 4a). This was not observed in the alloy JZ5. The better oxidation resistance of the A15 phase in the alloy JZ5 at 800 °C was attributed to the increase in its <Si> and Ti contents and the decrease in the concentration of W (Figure 10a).

#### 5.4.2. Oxidation at 1200 °C

Compared with the alloy JZ3 [47], the substitution of Ta by Mo in JZ4 (i) changed the oxidation kinetics from linear for the majority of the duration of the oxidation experiment in JZ3 to parabolic for the whole experiment in JZ4, (ii) reduced the weight change by 44.5%, (iii) eliminated edge cracking of the scale and (iv) supressed scale spallation at 1200 °C. Compared with the alloy JZ3+ [47], (v) the weight gain of JZ4 was reduced only slightly (4%) and (vi) there was no scale spallation in JZ4 and JZ5. The reduction by 37% in the weight gain of the alloy JZ5 compared with the alloy JZ4 was attributed to the beneficial effect of the increased concentration of Ti on the oxidation resistance, in agreement with [68]. Furthermore, the increase in Ti content in JZ5 did not affect the adherence of the scale. In other words, the substitution of Ta by Mo notably improved the oxidation at both temperatures. The parabolic rate constants of the polycrystalline alloys JZ3+ [47], JZ4 and JZ5, all of which did not suffer from scale spallation, were the closest to that of the single crystal Ni superalloy CMSX-4, which gained 0.4 mg/cm^2^ after 50 h and had k_p_ ≈ 4 × 10^−12^ g^2^ cm^−4^ s^−1^ at 1200 °C [1].

When Sn was added (2–8 at.%) to the MASC alloy Nb-25Ti-16Si-8Hf-2Al-2Cr, all alloys gained more than 50 mg/cm^2^ after 50 h exposure at 1200 °C [44]. The addition of 5 at.% Sn to Nb-24Ti-18Si-2Mo-5Al-5Cr-5Hf reduced the rate constant at 1200 °C but the weight gain was high, about 90 mg/cm^2^ after 100 h exposure. A mass gain of about 40 mg/cm^2^ was observed in the alloy Nb-24Ti-18Si-5Ge-5Al-5Cr after 100 h at 1200 °C [43]. Comparison of the data of the alloys mentioned above with that for JZ3+ [47], JZ4 and JZ5 shows (1) that the oxidation resistance of Nb-silicide-based alloys at 1200 °C was enhanced dramatically when Sn and Ge were added together with Al, Ce, W and Ta or Mo, and with Hf at low (≤1 at.%) concentration and (2) that the oxidation improved with the increase in the Sn and/or Ti concentrations in the alloys.

**Figure 11:**
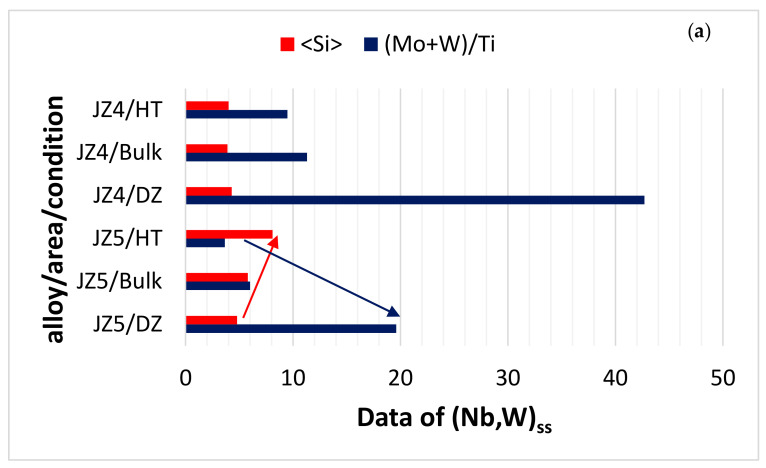

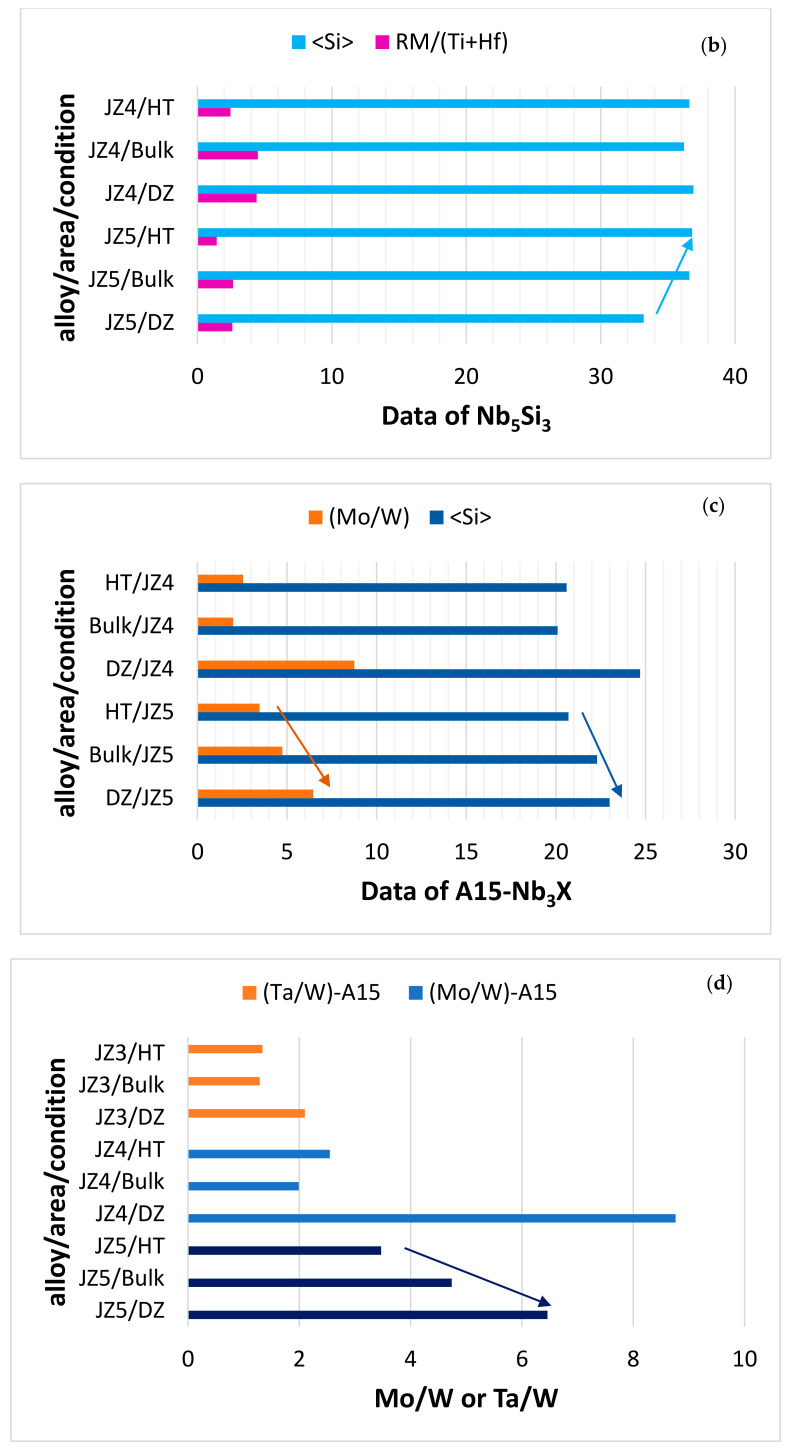

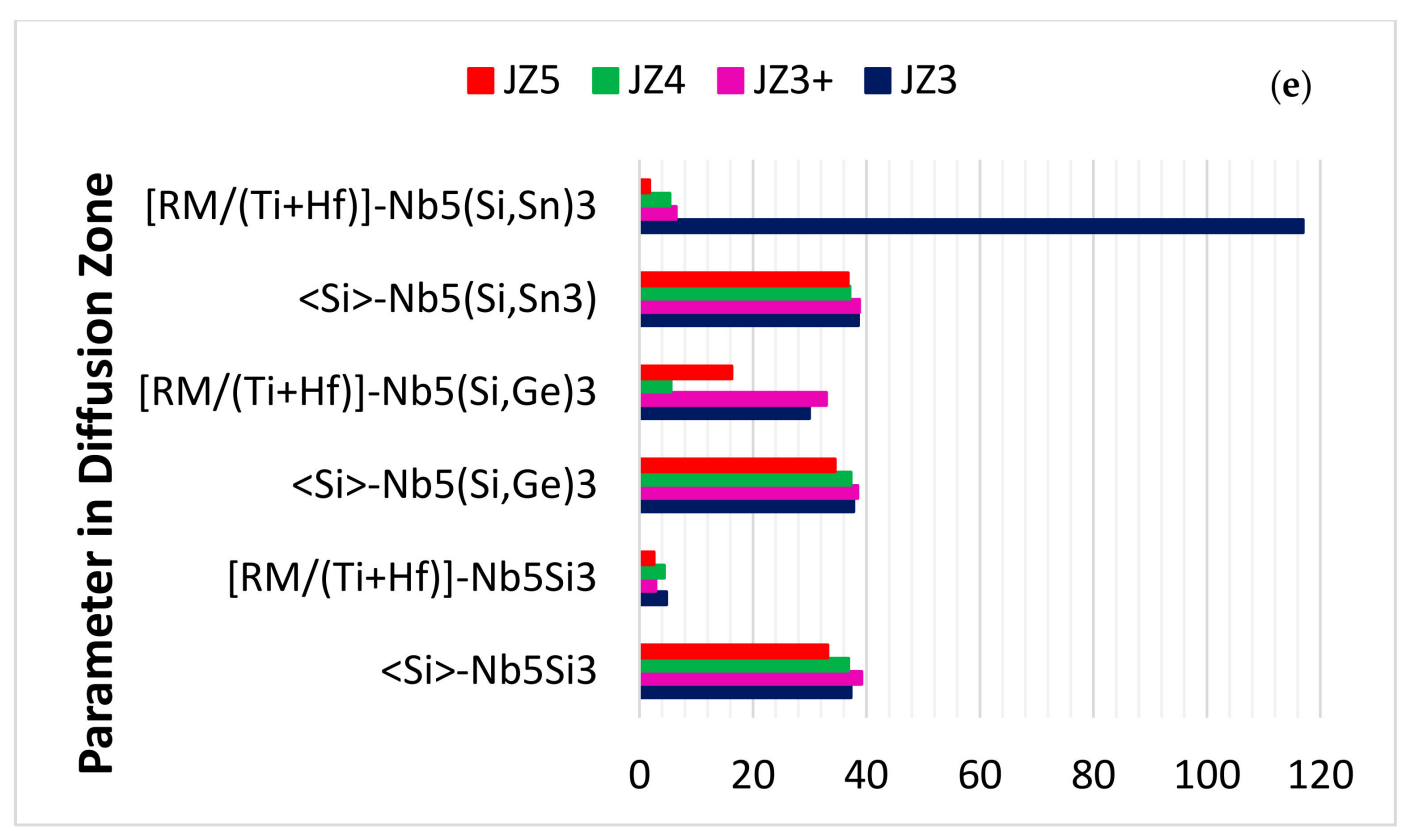
Data for 1200 °C for phases in different alloys. (**a**) Data for (Nb, W)_ss_, parameters <Si>, (Mo + W)/Ti, (**b**) data for Nb_5_Si_3_, parameters <Si>, RM/(Ti + Hf), (**c**) data for A15-Nb_3_X, parameters <Si>, Mo/W, (**d**) data for A15-Nb_3_X, parameters Ta/W or Mo/W, (**e**) data for phases in the diffusion zone of the alloys JZ3, JZ3+ [27], JZ4 and JZ5. RM = Mo or Ta, <Si> = Al + Ge + Si + Sn. Note that <Si> = Al + Ge + Si + Sn when used for the A15 phase.

Changes in the chemical composition of the (Nb,W)_ss_, Nb_5_Si_3_ and A15-Nb_3_X phases in the diffusion zone and bulk of the oxidised specimens of the alloys JZ4 and JZ5 are compared with the bulk of the heat-treated alloys in Figure 11a–c. In both alloys, the (Mo + W)/Ti ratio of the solid solution was highest in the diffusion zone, owing to the depletion of its Ti for the formation of the Ti-rich oxide in the scale and the increase in its Mo and W contents (Tables S5 and S6 and Table 6), and the <Si> content was lowest in the diffusion zone of JZ5, which indicates a stronger contribution of Al,Ge,Si,Sn in the formation of the scale compared with JZ4, where <Si> did not change significantly (Figure 11a). Note that in Table 6, two sets of data for given for the bulk, namely data for the bulk of the oxidised specimen and below this are the data in italics for the heat-treated alloy. In both the alloys of this study, the Nb_5_Si_3_ contributed Ti and Al,Ge,Si,Sn towards the formation of the scale, the latter contribution was stronger in JZ5 compared with JZ4 (Figure 11b, Table 6). Owing to segregation of solutes in the diffusion zone, the <Si> content and the Mo/W ratio of the A15-Nb_3_X increased in the diffusion zone of both alloys, particularly in the alloy JZ5 that exhibited a stronger trend compared with the Ta/W ratio of the alloy JZ3 (Figure 11c,d, Table 6). Considering the alloys JZ3, JZ3+ [47], JZ4 and JZ5, the trend in the <Si> content in the diffusion zone was essentially the same, with the lowest <Si> for JZ5 (Figure 11e) and the RM/(Ti + Hf) ratio of the Nb_5_(Si, Sn)_3_ in JZ5 was the lowest owing to its high Ti and Sn concentrations (Figure 11e and Table 6). It should be noted that both Sn and Ti reduce the hardness of Nb_5_Si_3_ [63]. It is suggested that the formation of thin oxide scales with low stresses due to the growth of the oxides in the scales, the presence of Nb_5_(Si, Sn)_3_ in the diffusion zone and the low vol.% of the (Nb, W)_ss_ contributed to increase the deformation capability of the area around the scale/substrate interface and thus improved the adhesion of the scales in the alloys JZ3, JZ3+ [47], JZ4 and JZ5, particularly in the latter three alloys.

Geng et al. [56], Vellios [76], Knittel et al. [44] and Xu et al. [41,42] reported that in their Sn-containing alloys the Sn segregated at the oxide scale/diffusion zone interface at 1200 °C where the Nb_3_Sn and Nb_5_Sn_2_Si compounds were formed. The latter compound is not stable at 1200 °C according to [66]. The stability of the Nb_5_Sn_2_Si compound at 1200 °C has also been confirmed in oxidised Nb-24Ti-18Si-5Al-5Cr-5Sn [42]. In other words, prior to this work, the formation of Nb_5_Sn_2_Si at 1200 °C had been confirmed only in oxidised Nb-silicide-based alloys where Al, Cr and Sn were present simultaneously, which would suggest that contamination by oxygen could stabilise this compound at the said temperature. The TM_5_Sn_2_X compound that formed in JZ4-AC and JZ5-AC was not stable in the heat-treated alloys and in the oxidised alloys at 1200 °C. This would suggest that the simultaneous addition of Ge and Sn with Mo and W was also key to TM_5_Sn_2_X not being stable at 1200 °C.

A common observation in the alloys JZ3, JZ3+ [47], JZ4 and JZ5 was the segregation of Ge and Sn below the scale/substrate interface (Figure 7) and formation of Sn and Ge containing intermetallic phases in the diffusion zone (Table 6). Segregation of solutes in the substrate just below the scale/substrate interface has been studied in [41,42,45,56,76]. Table 7 shows the solutes that are predicted to segregate to the surfaces of binary Nb-X alloys. Note that Table 7 is an expansion of the Table 7 in [41] to include data for Ge, Mo and Hf.

According to theory A, the solute of the alloy with lower heat of sublimation should segregate to the surface; according to theory B, the larger the solute atom relative to the solvent, the stronger the surface segregation; according to theory C, surface segregation should occur when, owing to solute distribution (partitioning), the melt is richer in solute than the solid, and according to theory D, the element with the lower surface energy segregates.

Ge- and Sn-containing intermetallics and Nb_ss_ rich in refractory metals formed in the diffusion zones of the alloys JZ3, JZ3+ [47], JZ4 and JZ5 at 1200 °C. The solid solution in the alloys JZ3 and JZ3+ was very rich in W and extremely poor in Ti (see Table 6 and [47]). Segregation of W or Ta to the surface is not expected in binary Nb-X (X = Ta, W) alloys.

**Figure 12:**
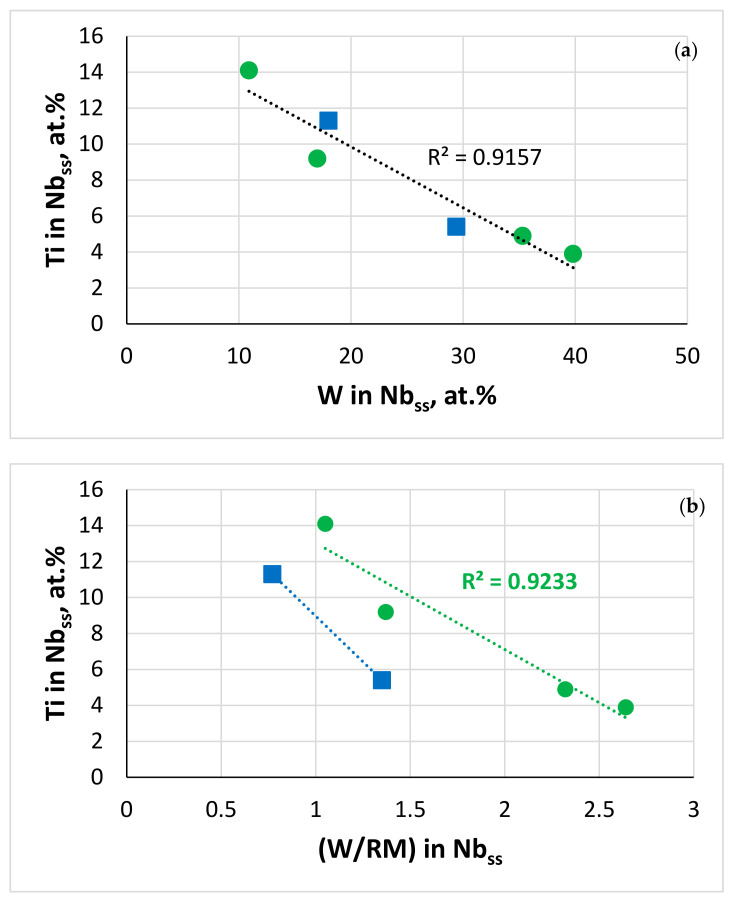

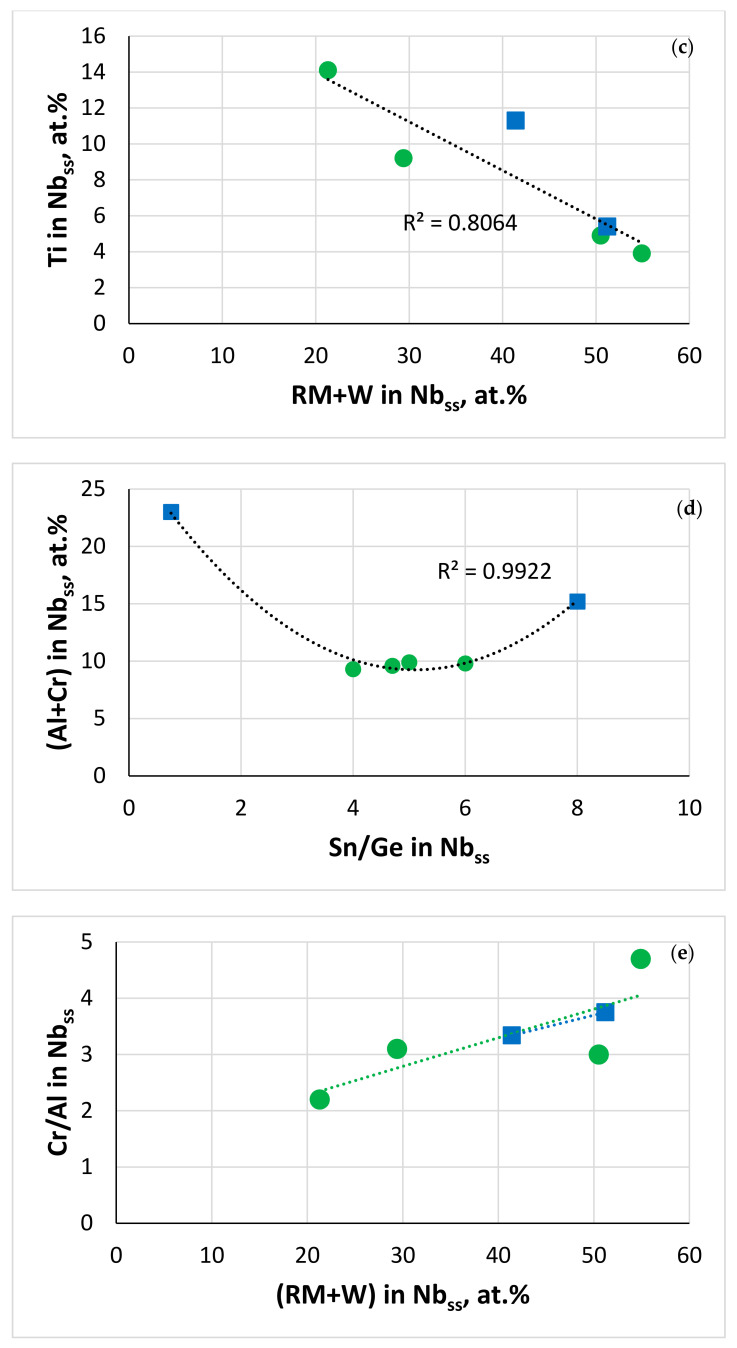
Data for the chemical composition of the solid solution. Squares for the alloys JZ4, JZ5, circles for Ta-containing alloys JZ1, JZ2 [46], JZ3, JZ3+ [47]. (**a**) Ti versus W in Nb_ss_, (**b**) Ti versus W/RM in Nb_ss_, (**c**) Ti versus RM + W in Nb_ss_, (**d**) Al + Cr in Nb_ss_ versus Sn/Ge in Nb_ss_, (**e**) Cr/Al in Nb_ss_ versus RM + W in Nb_ss_. RM = Mo or Ta, in (**e**) R^2^ = 0.6404 for linear fit of all data.

**Figure 13:**
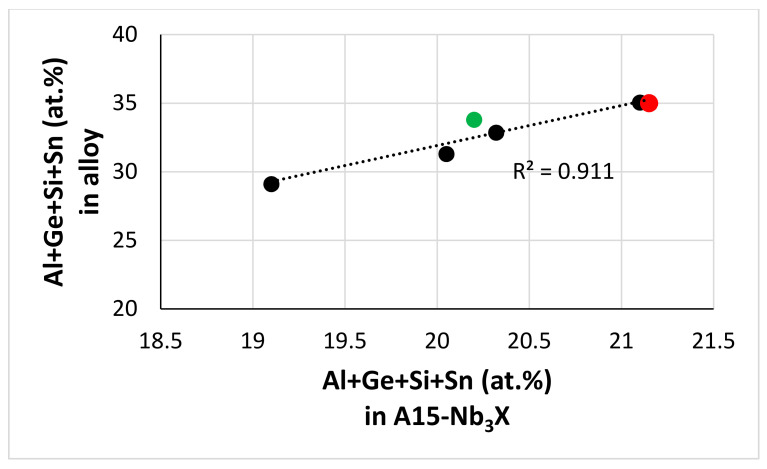
Relationship between the Al + Ge + Si + Sn content in alloys and A15-Nb_3_X. Data for the alloys JZ2 [46], JZ3, JZ3+ [47], JZ4 and JZ5. Red for JZ5, green for JZ4.

The Ti concentration in the solid solution decreased with increasing (i) W concentration [62], (ii) W/RM ratio and (iii) RM + W content (RM = Mo,Ta) (Figure 12a–c). The Al + Cr concentration of the solid solution did not depend on its Sn/Ge ratio in the Ta-containing alloys and was highest in JZ5 (Figure 12d), and the Cr/Al ratio increased with its RM + W content (RM = Mo, Ta) (Figure 12e). In the diffusion zone, as the concentration of Ti decreased in the phases owing to its consumption for the formation of the scale, in the poorer Ti solid solution the W and Mo + W contents and the W/Mo ratio increased. The Al and Cr concentrations in the solid solution were also affected, particularly in JZ4 and JZ5, where the dependence of the Al + Cr content on the Sn/Ge ratio was stronger than in the Ta-containing alloys. The Cr/Al ratio in the solid solution increased because of the increased Mo + W content, and the decrease in the Al content was more severe compared with that of Cr (Tables S5 and S6) because decrease in the Ti concentration in Nb_ss_ results to lower Al and Cr concentrations [77]. The Al + Ge + Si + Sn content of the A15 increased with that of the alloy (Figure 13). Compared with JZ5-HT, the Mo/W and Al + Ge + Si + Sn content of the A15 increased in the bulk of the oxidised alloy and its diffusion zone (Table 6) and the Mo/W ratio was higher than the Ta/W ratio of JZ3 [47] owing to the lower solubility of Ta in the A15 phase than that of Mo.

The solid solution is the Achilles heel of Nb-silicide-based alloys regarding their oxidation [68,75]. The exceptional oxidation resistance of the alloys JZ4 and JZ5 at both temperatures benefited from the very low vol.% of (Nb,W)_ss_ that resulted from the increased Ge + Sn content in each alloy (Figure 9). The oxidation of the Nb_5_Si_3_ silicide is superior to that of Nb. One would expect the precipitation of Nb_ss_ in Nb_5_Si_3_ to decrease the oxidation resistance of the latter if it were to be contaminated by oxygen. This was not observed, as both alloys were not contaminated by oxygen after the heat treatment and after isothermal oxidation at 800 and 1200 °C. The inferior oxidation of the Ta-containing alloys JZ3 and JZ3+ [47], which also had low vol.% (Nb,W)_ss_, the same phases in the diffusion zone and precipitates of Nb_ss_ in Nb_5_Si_3_, would suggest that the chemical composition of the phases (Tables S3–S6 and Table 6, Figure 10, Figure 11, Figure 12 and Figure 13) played a key role regarding the control of the contamination by oxygen and the reduction of the diffusivity of oxygen in the alloys.

### 5.5. Further Comments on Experimental Data and NICE Calculations

The alloys JZ4 and JZ5, and the other alloys in Table 4, with the exception of JZ3+, did not pest at 800 °C. The alloys JZ4 and JZ5 followed parabolic kinetics with rate constants that were of the same order as the best Ti-rich Nb-silicide-based alloys based on KZ5 (Nb-24Ti-18Si-5Al-5Cr) (Table 3 in [1]) and two orders higher than the k_p_ of CMSX-4 at 815 °C [1]. It is remarkable that their oxidation in the pest regime is better than that of boron containing Ti-rich Nb-silicide-based alloys that follow linear kinetics at 800 °C (Table 3 in [1]). Furthermore, it is noteworthy that the oxidation behaviour in the pest regime of all the alloys in Table 4, which are also RCCAs, is much better than that of the RCCAs that were recently reviewed in [2].

At 1200 °C, the oxidation resistance of the alloys JZ4 and JZ5 (and JZ3+) was also better than that of the other alloys in the Table 4. Both alloys (and JZ3+) followed parabolic kinetics with rate constants better than or of the same order compared with Boron containing Ti-rich Nb-silicide-based alloys (Table 3 in [1]). The alloy JZ5 exhibited the lowest k_p_ that was two orders of magnitude higher than that of CMSX-4 at at 1200 °C.

In our opinion, it is remarkable that the two alloys of this study with (i) RM additions, (ii) low densities (albeit higher than the densities of Boron containing Ti-rich Nb-silicide-based alloys based on KZ5 (ρ ≤ 6.8 g/cm^3^ [1]) and (iii) probably good creep at the creep goal conditions (Section 5.3), surpass or match up with the oxidation properties of Ti-rich Nb-silicide-based alloys with or without Boron addition.

**Figure 14:**
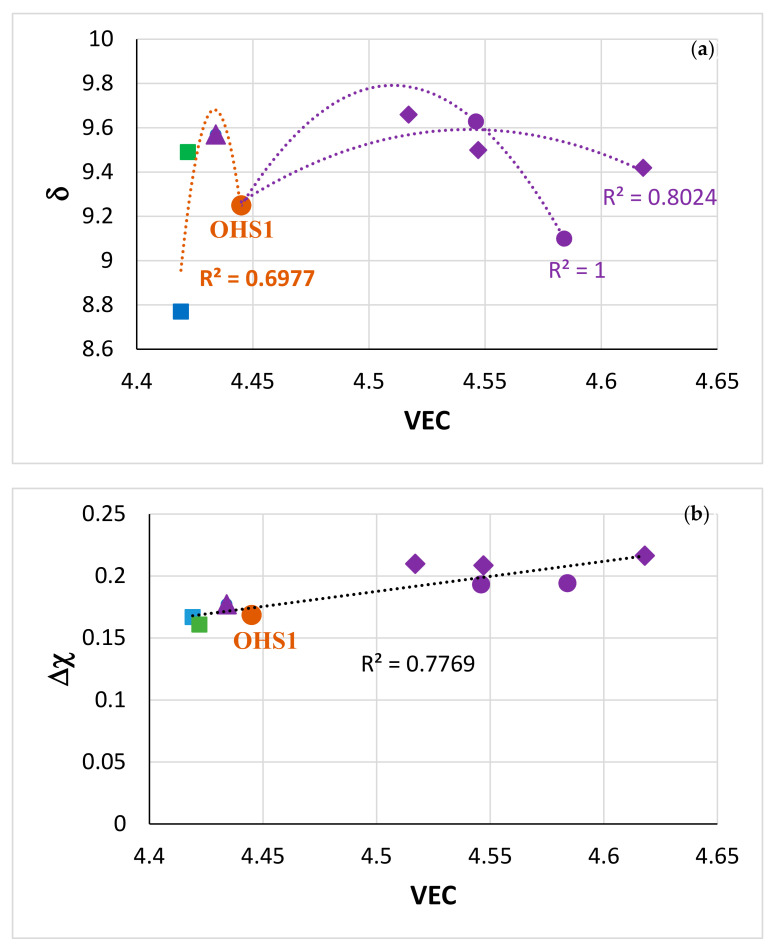
(**a**) δ versus VEC map and (**b**) Δχ versus VEC map of Nb-silicide/RCCA alloys, data for the alloys OHS1 [45], JZ3, JZ3+ [47], JG6 [56], EZ8 [58], ZF9 [59], for nominal compositions see Abbreviations. Diamonds for alloys JZ4, JZ5, NT1 [78], purple circles for alloys JZ3, JZ3+, triangle JG6, green square EZ8, blue square ZF9, brown circle OHS1.

The oxidation of the alloy OHS1 [45] was the stimulus for the study of the alloys discussed in this paper (Section 1 and Section 2) and in [47]. The oxidation of JZ4 and JZ5 at 800 and 1200 °C was better than that of the alloy OHS1 (Table 4). The parameters VEC, Δχ, δ are key in the alloy design methodology NICE [20]. Maps based on these parameters describe alloying behaviour of alloys and their phases [1,7,21,47,50,63,64]. Figure 14 shows the δ versus VEC and Δχ versus VEC maps of Nb-silicide-based alloys with Ge or Sn or Ge + Sn additions and with or without RM (=Mo, Ta, W) addition. Note that the alloys included in the Figure 14 are also RCCAs. The maps in the Figure 14 clearly separate the alloys without W addition (on the left hand side of OHS1) from those with Mo and W or Ta and W addition (on the right hand side of OHS1), and show (i) that the addition of Mo, W in JZ4 and JZ5 (and of Ta, W in JZ3+) increase both VEC and δ compared with OHS1 and (ii) that all the alloys fall in the same line in the Δχ versus VEC map (Figure 14b). The latter figure also shows that the addition of Mo, W in JZ4 and JZ5 and Ta, W in JZ3 and JZ3+ increased VEC and Δχ.

**Figure 15:**
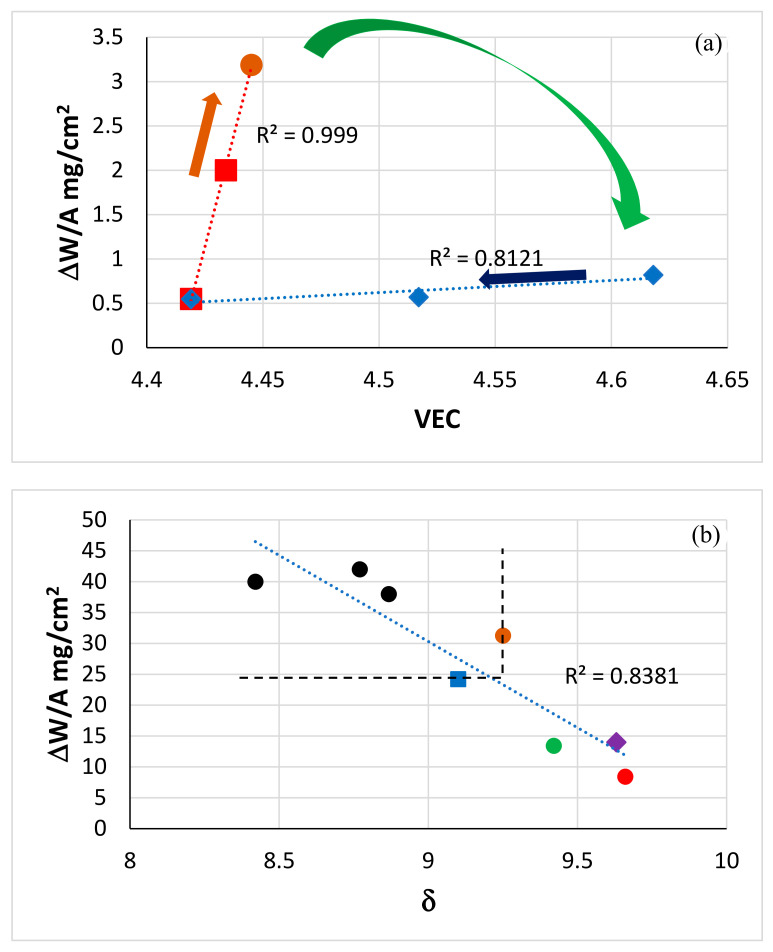
Weight change ΔW/A versus (**a**) VEC and (**b**) δ at (a) 800 °C and (**b**) 1200 °C. In (**a**) the two lines cross at the data point of the alloy ZF9 [59]. Red ZF9, JG6 [56], orange OHS1 [45], blue ZF9, JZ4 and JZ5. In (**b**) the data are for the alloys ZF6 [59], ZX8 [42], ZF9, OHS1, JZ3, JZ3+ [47], JZ4, JZ5. Red JZ5, green JZ4, square JZ3, diamond JZ3+, orange OHS1. For all data R^2^ = 0.8381, for the alloys OHS1, JZ4 and JZ5 R^2^ = 0.8395. For nominal compositions of alloys see Abbreviations. For dashed lines see text.

Trends of the aforementioned parameters are linked with the improvement of specific properties [20], for example NICE predicts that oxidation resistance increases with decreasing VEC and increasing δ. The data in the Figure 15a are for oxidation at 800 °C and show the decrease in ΔW/A with decreasing VEC, in agreement with NICE. All the alloys in this figure are also RCCAs. The two lines, which are consistent with the linear fit of data, cross at the data point of the alloy ZF9 (Nb-24Ti-18Si-5Al-5Cr-5Ge-5Hf [43,59]). The red line is for Ti-rich Nb-silicide-based alloys. Note (i) that the simultaneous addition of Ge and Sn in OHS1 increased the weight gain at 800 °C [45] and VEC (indicated by the orange arrow) (ii) that the addition of Mo and W in JZ4 and the decrease in the Ti content, compared with OHS1, caused the clockwise shift in the map from OHS1 to JZ4, which is indicated by the green arrow, (iii) that the increase in the Ti content in JZ5 reduced both the ΔW/A and VEC compared with JZ4, which is indicated by the blue arrow and (iv) that the slope of the blue line is significantly lower than that of the red line. The data in the Figure 15b are for oxidation at 1200 °C and show the decrease in ΔW/A with increasing δ, in agreement with NICE. In this map, all the alloys fall in the same line with R^2^ = 0.8381. The dashed vertical and horizontal lines delineate the area of Nb-silicide-based alloys with scale spallation. Compared with OHS1, the addition of RMs in JZ3+, JZ4 and JZ5 reduced both weight gain and δ.

**Figure 16:**
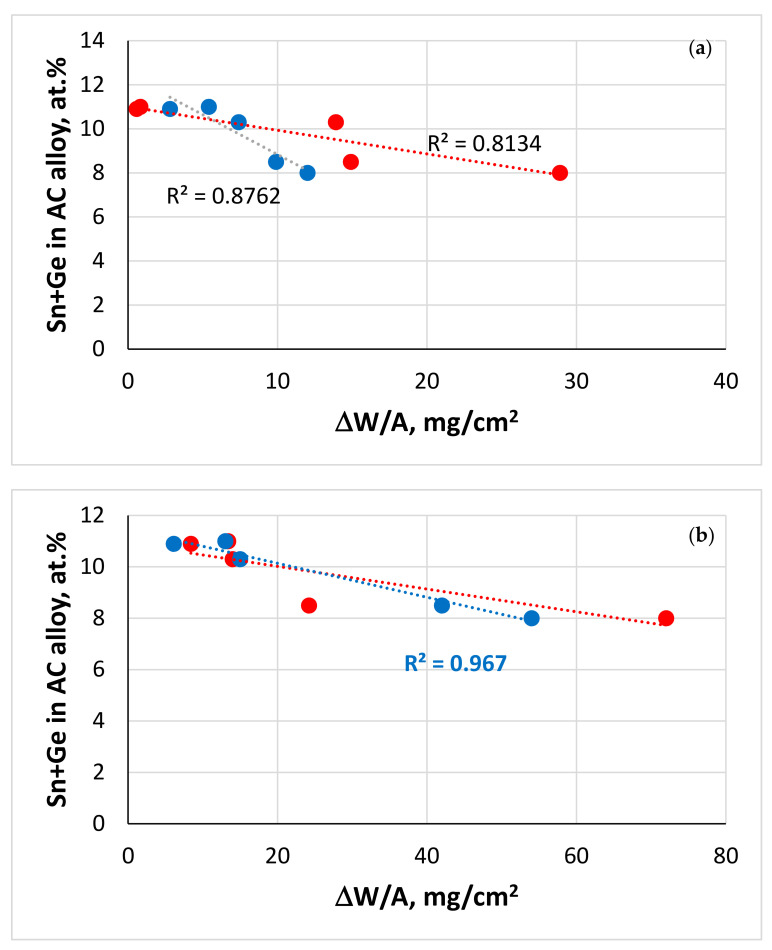

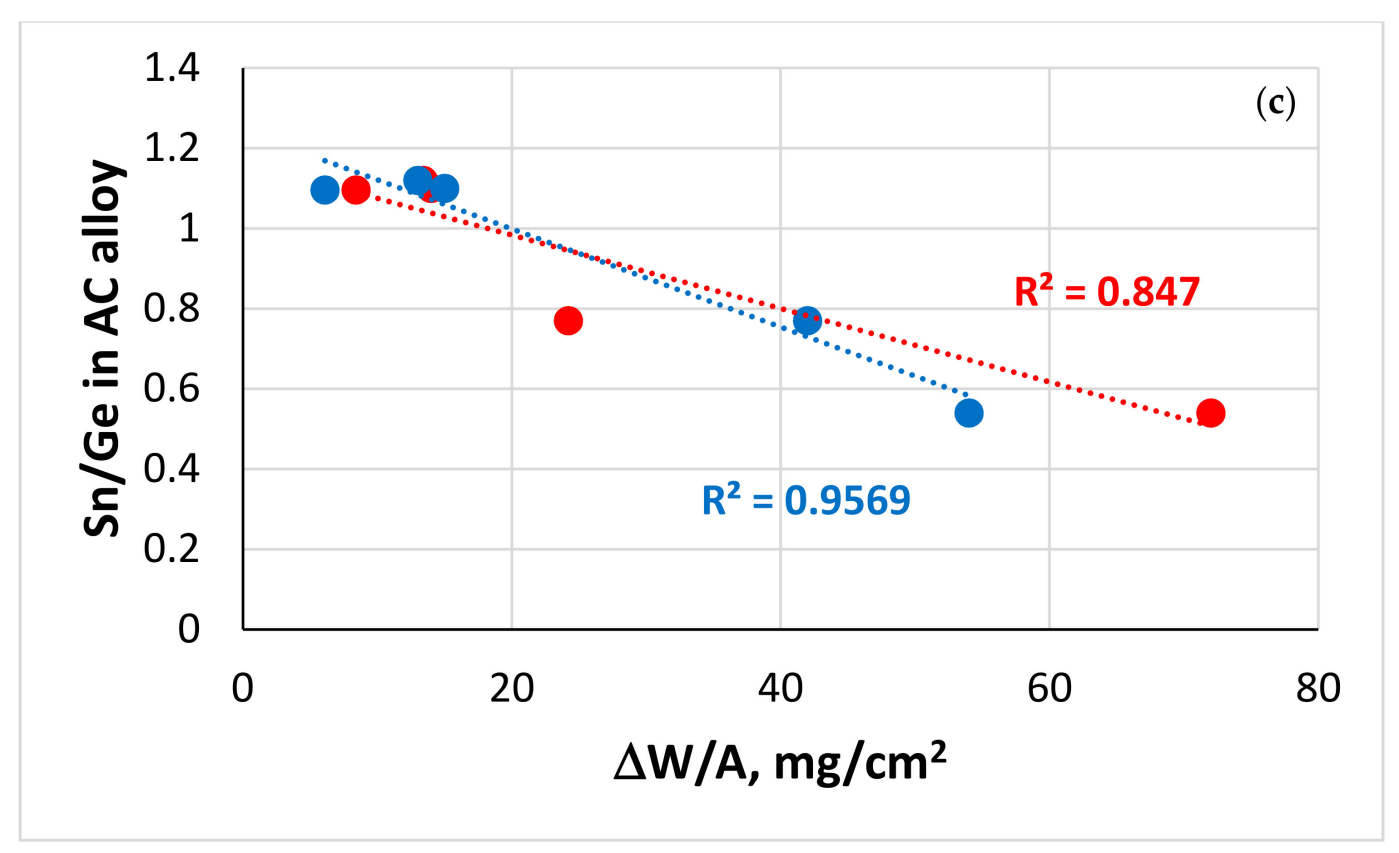
Weight change ΔW/A versus (**a**,**b**) Sn + Ge and (**c**) Sn/Ge in as-cast alloys JZ2 [46], JZ3, JZ3+ [47], JZ4 and JZ5 at (**a**) 800 °C and (**b**,**c**) 1200 °C. Experimental data shown with red colour, calculated data by NICE [20] shown with blue colour. AC = as cast.

Figure 15 showed that the experimental data corroborated the trends of the parameters VEC and δ predicted by NICE for oxidation resistance in Nb-silicide-based alloys. Note that these trends were followed for alloys that are also RCCAs. Figure 16 compares the experimental data about weight change at 800 and 1200 °C with the calculated ΔW/A using NICE. The weight change decreases with the Sn + Ge content (Figure 16a,b) and the Sn/Ge ratio (Figure 16c) in the alloys. The agreement between calculations and experiment is considered good for both temperatures. Note the link between Sn + Ge or Sn/Ge with the vol.% Nb_ss_ in the alloys, and the link between the Al + Cr content in Nb_ss_ with the Sn/Ge ratio, which were discussed earlier (see also Figure 9 and Figure 12).

## 6. Summary, and Suggestion for Future Work

We studied the microstructures, MACSi and isothermal oxidation of the alloys JZ4 and JZ5, calculated their average creep rate due to intrinsic resistances for the creep goal conditions, and compared experimental data with the calculations of the alloy design methodology NICE. We also compared properties of the alloys with those of other RCCAs studied to date and reviewed in [2]. The densities of both alloys were less than 7.3 g/cm^3^ and lower than the density of multiphase RCCAs with bcc solid solution + M_5_Si_3_ silicide(s) in their microstructure. There was macrosegregation of Si in both alloys. The latter had the same phases in their as-cast microstructures, namely βNb_5_Si_3_, αNb_5_Si_3_, A15-Nb_3_X (X = Al, Ge, Si, Sn), TM_5_Sn_2_X (X = Al, Ge, Si), C14-Cr_2_Nb, and no solid solution. After the heat treatment at 1500 °C for 100 h a low volume fraction of an Mo- and W-rich solid solution was observed in both alloys together with βNb_5_Si_3_, αNb_5_Si_3_ and A15-Nb_3_X but not the TM_5_Sn_2_X, whereas the Laves phase was stable only in JZ4. At 800 °C both alloys did not pest, and there was no spallation of their scales at 1200 °C. At both temperatures, both alloys followed parabolic oxidation kinetics and their weight changes were lower than those of Ti-rich Nb-silicide-based alloys. The oxidation of both alloys was superior to that of other RCCAs studied to date [2]. Calculated Si macrosegregation, solid solution volume fractions, chemical compositions of solid solution and Nb_5_Si_3_, and weight changes in isothermal oxidation at 800 and 1200 °C using the alloy design methodology NICE agreed well with the experimental results.

Considering the experimental data for oxidation, MACSi and room temperature strength and the calculated data for oxidation, MACSi and creep rates of the alloys JZ4 and JZ5, it is suggested that both alloys are worthy further investigations that could consider (i) evaluation of their cyclic oxidation, (ii) assessment of their creep properties, and (iii) the scale up of alloy making to large buttons and ingots [1,62] to confirm reproducibility of properties. In our opinion, ultra-high temperature Nb-Mo-W-Ti-Cr-Hf-Al-Ge-Si-Sn RMICs, RCCAs and RHEAs are worthy of development owing to their promise to meet property goals and/or offer a balance of properties. The alloys JZ4 and JZ5 are a good basis for the design of new alloys.

In the introduction we discussed that RMICs, RCCAs and RHEAs intended for application in aero engines would require environmental coatings (ECs) of the BC/TGO/TC type [36,79]. BC Nb-Ti-Si-Al-Hf HEAs forming αAl_2_O_3_ TGO [80,81] could be suitable and compatible with Nb-Mo-W-Ti-Cr-Hf-Al-Ge-Si-Sn UHTMs. A good starting point for the design of HEA and/or “conventional” alloys for BC application in ECs could be the (Al/Si)_alloy_ versus [Nb/(Ti + Hf)]_alloy_ maps in Figure 13 in [81], and the Δχ versus VEC, δ versus VEC and Δχ versus δ maps in Figure 13 in [82].

## Figures and Tables

**Table 1:** Density of the as-cast (AC) alloys and area % of selected phases in the bulk of the as-cast and heat-treated (HT) alloys JZ4 and JZ5.

Alloy	Density (g/cm^3^)	Solid Solution (%)	A15 (%)
JZ4-AC	7.28 ± 0.01	-	19.8 ± 3.2
	7.27–7.29		17.3–23.4
JZ4-HT ^a^	-	3.3 ± 0.5	12.2 ± 2.0
		2.7–3.6	10.9–14.5
JZ5-AC	6.91 ± 0.06	-	12.7 ± 1.1
	6.87–7.01		11.4–13.4
JZ5-HT ^a^	-	2.3 ± 0.3	10.6 ± 0.3
		2.1–2.6	10.4–10.9

^a^ Some hafnia may be included in the measurement because the oxide exhibited the same contrast as the solid solution.

**Table 2:** Average chemical compositions (at.%) and summary of phases in the as-cast and heat-treated alloys JZ4 and JZ5.

Alloy	
JZ4	JZ5
**As Cast**	
Nb-12.5Ti-17.8Si-6.2Mo-2.3W-5.8Sn-5.2Ge-1.1Hf-5Al-5.2Cr	Nb-20.4Ti-19.2Si-6.3Mo-1.1W-5.7Sn-5.2Ge-0.9Hf-4.5Al-4.7Ge
Nb_5_Si_3_, Ti-rich Nb_5_Si_3_	Nb_5_Si_3_, Ti-rich Nb_5_Si_3_
A15-Nb_3_X, Ti-rich A15	A15-Nb_3_X, Ti-rich A15
TM_5_Sn_2_X	TM_5_Sn_2_X
C14-NbCr_2_ Laves	C14-NbCr_2_ Laves
HfO_2_	HfO_2_
**Heat Treated**	
Nb-12.3Ti-18.2Si-5.8Mo-2.2W-5.4Sn-5.4Ge-1.2Hf-4.8Al-4.8Cr	Nb-20.5Ti-18.8Si-6.2Mo-1.2W-5.7Sn-5.6Ge-1Hf-4.7Al-4.9Cr
(Nb,W)_ss_	(Nb,W)_ss_
Nb_5_Si_3_, Ti-rich Nb_5_Si_3_	Nb_5_Si_3_, Ti-rich Nb_5_Si_3_
A15-Nb_3_X	A15-Nb_3_X
C14-NbCr_2_ Laves	
HfO_2_	HfO_2_

**Table 3:** Weight gains and parabolic oxidation rate constants of the alloys JZ4 and JZ5 after isothermal oxidation at 800 and 1200 °C for 100 h.

Alloy	Weight Gain (mg/cm^2^)	Rate Constant *k*_p_ (g^2^ cm^−4^ s^−1^)	Weight Gain (mg/cm^2^)	Rate Constant *k*_p_ (g^2^ cm^−4^ s^−1^)
	800 °C		1200 °C	
JZ4	0.82 (100 h)	1.02 × 10^−12^ (0–100 h)	13.43 (100 h)	5.04 × 10^−10^ (0–100 h)
		9.9 × 10^−12^ (0–9 h)		2.92 × 10^−10^ (0–14 h)
		9.4 × 10^−13^ (9–100 h)		5.44 × 10^−10^ (14–100 h)
JZ5	0.57 (100 h)	7.33 × 10^−12^ (0–100 h) 1.2 × 10^−11^ (45–100 h)	8.43 (100 h)	1.92 × 10^−10^ (0–100 h) 9.46 × 10^−11^ (0–9 h) 2.04 × 10^−10^ (9–100 h)

**Table 4:** Density (ρ, g/cm^3^) and oxidation properties (weight change, ΔW/A (mg/cm^2^), rate constants linear (K_l_, g·cm^−2^s^−1^), parabolic (K_p_, g^2^cm^−4^s^−1^), pest oxidation, scale spallation—SP) of Nb-silicide-based alloys/refractory metal complex concentrated alloys (RCCAs) **^,++^ with Al, Ge, Si, Sn, TM, RM additions after isothermal oxidation at 800 or 1200 °C for 100 h. TM = Cr, Hf, Ti, RM = Mo, Nb, Ta, W. Oxidation data from specimens were cut from cast alloy buttons which were prepared using arc melting.

Alloy/RCCA	Stable Phases ^#^	ρ	800 °C			1200 °C		
			ΔW/A	Pest	Rate Constant	ΔW/A	SP ^+^	Rate Constant
JG6	5-3, A15, C14	6.96	1.8	No	K_p_ = 1 × 10^−11^	90	Yes *	K_l_ = 2.8 × 10^−7^
EZ8	5-3, A15, C14	6.89	1	No	K_l_ = 2 × 10^−9^ (>20 h)	14	Yes	K_p_ = 6 × 10^−10^
					K_p_ = 5.3 × 10^−9^ (≤20 h)			
ZF9	ss,	6.96	0.55	No	K_p_ = 8.4 × 10^−13^	42	Yes	K_p_ = 1.8 × 10^−9^ (≤20 h)
	5-3							K_l_ = 1 × 10^−7^ (>20 h)
OHS1	5-3, A15, C14	6.78	3.2	No	K_p_ = 2.4 × 10^−11^	31.3	No	K_l_ = 1.1 × 10^−7^ (>3.1 h)
								K_p_ = 4.9 × 10^−10^ (≤3.1 h)
JZ3	ss, 5-3, A15, C14	7.94	14.9	No	K_l_ = 4.4 × 10^−8^	24.2	Edge cracks	K_l_ = 6 × 10^−8^
JZ3+	ss, 5-3, A15, C14	7.54	13.9	Yes	K_l_ = 6.5 × 10^−9^	14	No	K_p_ = 5.5 × 10^−10^
JZ4	ss, 5-3, A15, C14	7.28	0.82	No	K_p_ = 1 × 10^−12^	13.4	No	K_p_ = 5 × 10^−10^
JZ5	ss, 5-3, A15, C14,	6.91	0.57	No	K_p_ = 7.3 × 10^−12^	8.4	No	K_p_ = 1.9 × 10^−10^

* Poor adhesion, scale spalled off easily on handling, ^+^ scale spallation; ** nominal compositions (at.%), JG6 = 36Nb-24Ti-18Si-5Al-5Cr-5Hf-5Sn-2Mo [56], EZ8 = 38Nb-24Ti-18Si-5Al-5Cr-5Hf-5Sn [57,58], ZF9 = 38Nb-24Ti-18Si-5Al-5Cr-5Hf-5Ge [43,59], OHS1 = 38Nb-24Ti-18Si-5Al-5Cr-5Ge-5Sn [45]; ^++^ as-cast alloys unless indicated otherwise, for alloy designations see the Abbreviations; ^#^ phases in heat-treated (1400 °C/100 h, or 1500 °C/100 h) microstructures, ss = Nb solid solution, 5-3 = Nb_5_Si_3_, A15 = A15-Nb_3_X, C14 = C14-Cr_2_Nb Laves phase.

**Table 5:** Alloy parameters for the macrosegregation of Si in the cast alloys JZ5, ZF9, JG6, JG4, JG3 and KZ5.

Alloy	ΔH_m_ (kJ/mol)	T_m_ (K)		ΔH_m_/T_m_ (J/molK)		ΔH_m_^sd^/ΔH_m_^sp^		T_m_^sd^ (K)		T_m_^sp^ (K)		T_m_^sd^/T_m_^sp^		[ΔH_m_/T_m_] × [ΔH_m_^sd^/ΔH_m_^sp^]^−1^		MACSi (at.%)	
JZ5	28.4	2082	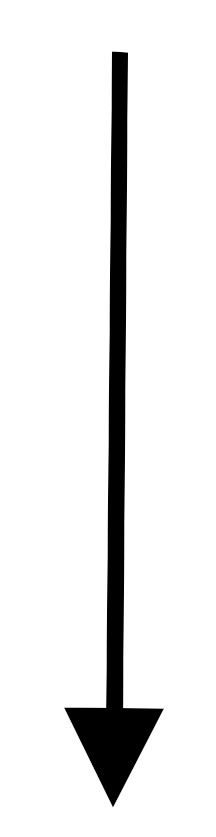	13.65	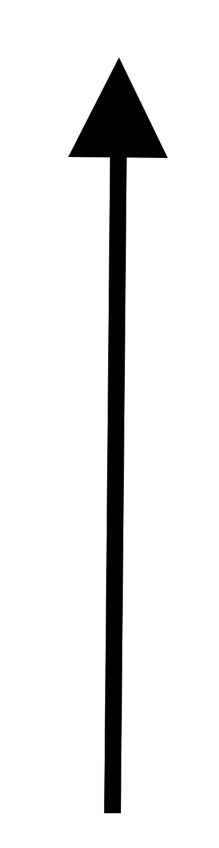	1.33	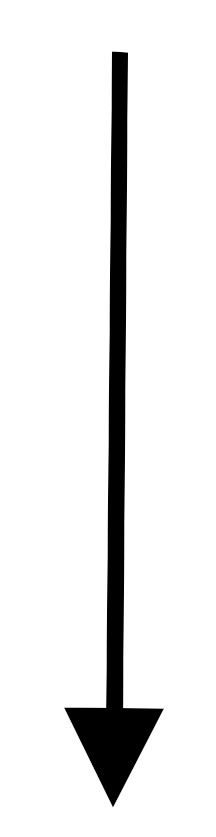	1624	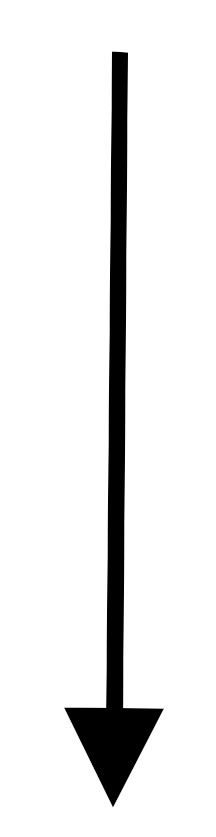	458	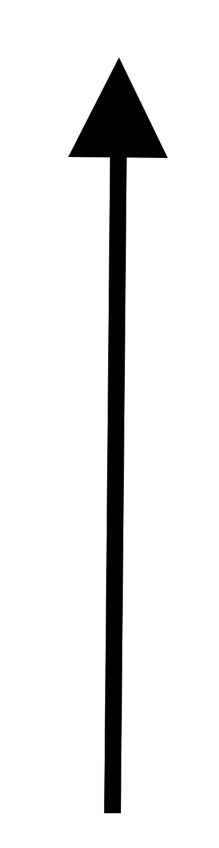	3.55	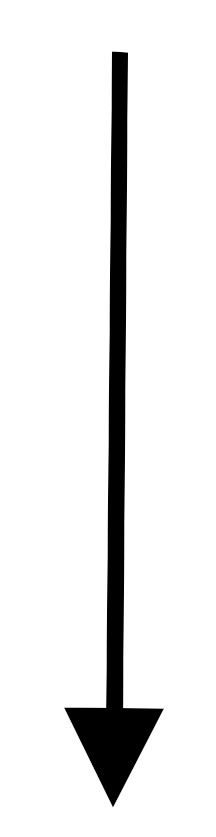	10.26	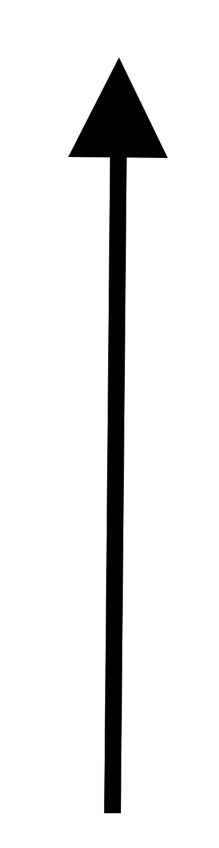	4.5	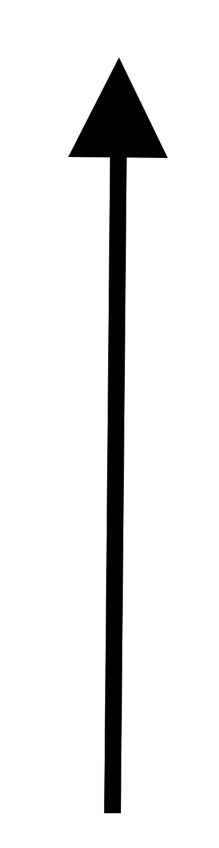
ZF9	27.71	2143		12.93		1.58		1755		388		4.52		8.18		3.1	
JG6	27.06	2154		12.8		1.66		1771		383		4.62		7.71		3	
JG4	28.33	2239		12.65		1.84		1877		362		5.19		6.88		2.7	
JG3	27.92	2245		12.44		2		1905		340		5.6		6.22		2.6	
KZ5	27.5	2239		12.28		2.05		1909		330		5.78		5.99		1.3	

KZ5 = Nb-24Ti-18Si-5Al-5Cr [61], ZF9 = Nb-24Ti-18Si-5Al-5Cr-5Ge-5Hf [59], JG3 = Nb-24Ti-18Si-5Al-5Cr-2Mo [52], JG4 = Nb-24Ti-18Si-5Al-5Cr-2Mo-5Hf [56], JG6 = Nb-24Ti-18Si-5Al-5Cr-2Mo-5Hf-5Sn [56].

**Table 6:** Phases in the diffusion zones and in the bulk of the alloys JZ3 and JZ3+ [47] and JZ4 and JZ5 after isothermal oxidation at 1200 °C. In italics are data for specific phases in heat-treated alloys.

Phase	Alloy			
**Diffusion Zone**	**JZ3**	**JZ3+**	**JZ4**	**JZ5**
(Nb,W)_ss_	W/Ta = 3.15	W/Ta = 3.64	W/Mo = 1.48	W/Mo = 1.24
	(Ta + W)/Ti = 152	(Ta + W)/Ti = 258	(Mo + W)/Ti = 42.7	(Mo + W)/Ti = 19.6
	<Si> = 5.5	<Si> = 4.2	<Si> = 4.3	<Si> = 4.8
A15-Nb_3_X	<Si> = 25.8	<Si> *	<Si> = 24.7	<Si> = 23
	Ta/W = 2.1	Ta/W *	Mo/W = 8.76	Mo/W = 6.46
Nb_5_(Si,Ge)_3_	<Si> = 37.8	<Si> = 38.5	<Si> = 37.3	<Si> = 34.5
	RM/(Ti + Hf) = 30	RM/(Ti + Hf) = 33	RM/(Ti + Hf) = 5.6	RM/(Ti + Hf) = 16.3
Nb_5_Si_3_	<Si> = 37.3	<Si> = 39.2	<Si> = 36.9	<Si> = 33.2
	RM/(Ti + Hf) = 4.8	RM/(Ti + Hf) = 2.9	RM/(Ti + Hf) = 4.4	RM/(Ti + Hf) = 2.6
Nb_5_(Si,Sn)_3_	<Si> = 38.6	<Si> = 38.8	<Si> = 37.1	<Si> = 36.8
	RM/(Ti + Hf) = 117	RM/(Ti+ Hf) = 6.5	RM/(Ti + Hf) = 5.4	RM/(Ti + Hf) = 1.8
**Bulk**	**JZ3**	**JZ3+**	**JZ4**	**JZ5**
(Nb,W)_ss_	^−^	^−^	W/Mo = 1.4	W/Mo = 1.08
			(Mo + W)/Ti = 11.3	(Mo + W)/Ti = 6
			<Si> = 3.9	<Si> = 5.8
	*W/Ta = 1.37 ^+^*	*W/Ta = 2.64 ^+^*	*W/Mo = 1.35 ^+^*	*W/Mo = 0.77 ^+^*
	*(Ta + W)/Ti = 3.2 ^+^*	*(Ta + W)/Ti = 14.1 ^+^*	*(Mo + W)/Ti = 9.48 ^+^*	*(Mo + W)/Ti = 3.66 ^+^*
	*<Si> = 7.3 ^+^*	*<Si> = 3.9 ^+^*	*<Si> = 4 ^+^*	*<Si> = 8.1 ^+^*
A15-Nb_3_X	<Si> = 19.7	<Si> = 20.3	<Si> = 20.1	<Si> = 22.3
	Ta/W = 1.29	Ta/W = 0.96	Mo/W = 1.99	Mo/W = 4.74
	*<Si> = 20.7 ^+^*	*<Si> = 20.5 ^+^*	*<Si> = 20.6 ^+^*	*<Si> = 20.7 ^+^*
	*Ta/W = 1.34 ^+^*	*Ta/W = 1.26 ^+^*	*Mo/W = 2.55 ^+^*	*Mo/W = 3.47 ^+^*
Nb_5_Si_3_	<Si> = 38	<Si> = 38.9	<Si> = 36.2	<Si> = 36.6
	RM/(Ti + Hf) = 4.6	RM/(Ti + Hf) = 4.98	RM/(Ti + Hf) = 4.5	RM/(Ti + Hf) = 2.65
	*<Si> = 38.6 ^+^*	*<Si> = 37.9 ^+^*	*<Si> = 36.6 ^+^*	*<Si> = 36.8 ^+^*
	*RM/(Ti + Hf) = 4.82 ^+^*	*RM/(Ti + Hf) = 3.23 ^+^*	*RM/(Ti + Hf) = 2.46 ^+^*	*RM/(Ti + Hf) = 1.44 ^+^*
Ti-rich Nb_5_Si_3_	<Si> = 37.9	<Si> = 36.4	<Si> = 35.9	<Si> = 36.6
	RM/(Ti + Hf) = 2.99	RM/(Ti + Hf) = 1.9	RM/(Ti + Hf) = 1.87	RM/(Ti + Hf) = 1.17
	*<Si> = 38.4 ^+^*	*<Si> = 38.4 ^+^*	*<Si> = 38.1 ^+^*	*<Si> = 37.9 ^+^*
	*RM/(Ti + Hf) = 2.48 ^+^*	*RM/(Ti + Hf) = 2.06 ^+^*	*RM/(Ti + Hf) = 1.84*	*RM/(Ti + Hf) = 1.08 ^+^*
C14-NbCr_2_	<Cr> = 61.7	^<^Cr> = 60.8	^<^Cr> = 59.6	<Cr> = 60.1
	Ta + W = 12.3	Ta + W = 14.1	Mo + W = 7	Mo + W = 7.9
	<Cr>/LFE = 1.77	<Cr>/LFE = 1.75	<Cr>/LFE = 1.78	<Cr>/LFE = 1.88
	*<Cr> = 63.3 ^+^*	*<Cr> = 62.1 ^+^*	*<Cr> = 61.3 ^+^*	-
	*Ta + W = 12.2 ^+^*	*Ta + W = 14.8 ^+^*	*Mo + W = 7.5 ^+^*	
	*<Cr>/LFE = 1.94 ^+^*	*<Cr>/LFE = 1.9 ^+^*	*<Cr>/LFE = 1.79 ^+^*	

* See [47], + data for HT alloy, <Si> = Al + Ge + Si + Sn, <Cr> = Cr + Al + Ge + Si + Sn, RM = Nb,Mo,Ta,W, LFE = C14-Laves forming element (Hf,Nb,Ta,Ti).

**Table 7:** Solutes in Nb-X binary alloys predicted to segregate to the surface.

Theory	Surface Segregating Element							
A	Al	Cr	Si	Sn	Ge	Mo	Hf	Ti
B	Al	-	-	Sn	Ge	-	-	Ti
C	-	Cr	Si	Sn	Ge	-	-	-
D	Al	Cr	Si	Sn	Ge	-	Hf	Ti

## Supplementary Materials

The following are available online at https://www.mdpi.com/1996-1944/13/20/4548/s1, Figure S1: X-ray diffractograms of the (a) as cast and (b) heat treated alloy, Figure S2: X-ray diffractograms of the (a) as cast and (b) heat treated alloy JZ5, Table S1: EDS analysis data (at.%) of the alloy JZ4, Table S2: EDS analysis data (at.%) for the alloy JZ5, Table S3: EDS analysis data (at.%) of phases in the alloy JZ4 after oxidation at 800 °C for 100 h, Table S4: EDS analysis data (at.%) of phases in the alloy JZ5 after oxidation at 800 °C for 100 h, Table S5: EDS analysis data (at.%) of phases in the alloy JZ4 after oxidation at 1200 °C for 100 h, Table S6: EDS analysis data (at.%) of phases in the alloy JZ5 after oxidation at 1200 °C for 100 h.

## Abbreviations

Alloys and their nominal compositions (at.%).

CM1	Nb-8.1Ti-21.1Si-5.4Mo-4W-0.7Hf
EZ8	Nb-24Ti-18Si-5Al-5Cr-5Hf-5Sn
JG1	Nb-18Si-5Al-5Cr-5Mo
JG2	Nb-24Ti-18Si-5Al-5Cr-5Mo
JG3	Nb-24Ti-18Si-5Al-5Cr-2Mo
JG4	Nb-24Ti-18Si-5Al-5Cr-5Hf-2Mo
JG6	Nb-24Ti-18Si-5Al-5Cr-5Hf-5Sn-2Mo
JZ1	Nb-12Ti-18Si-6Ta-2.5W-1Hf-2Sn-2Ge
JZ2	Nb-12Ti-18Si-6Ta-2.5W-1Hf-5Sn-5Ge
JZ3	Nb-12Ti-18Si-6Ta-2.5W-1Hf-5Sn-5Ge-5Al-5Cr
JZ3+	Nb-12Ti-18Si-6Ta-2.5W-1Hf-7.5Sn-5Ge-5Al-5Cr
KZ2	Nb-24Ti-18Si-8Cr-4Al
KZ5	Nb-24Ti-18Si-5Al-5Cr
KZ6	Nb-24Ti-18Si-6Ta-5Al-5Cr
KZ7	Nb-24Ti-18Si-5Al
KZ8	Nb-24Ti-18Si-8Cr-4Al
MASC	Nb-25Ti-16Si-8Hf-2Al-2Cr
OHS1	Nb-24Ti-18Si-5Al-5Cr-5Ge-5Sn
YG6	Nb-20Si-5Mo-3W
ZF1	Nb-18Si-5Ge
ZF5	Nb-24Ti-18Si-5Al-5Ge
ZF6	Nb-24Ti-18Si-5Al-5Cr-5Ge
ZF9	Nb-24Ti-18Si-5Al-5Cr-5Ge-5Hf
ZX7	Nb-24Ti-18Si-5Al-5Cr-2Sn
ZX8	Nb-24Ti-18Si-5Al-5Cr-5Sn
