# Supplementary material for: On the Microstructure and Properties of Nb-18Si-6Mo-5Al-5Cr-2.5W-1Hf Nb-Silicide Based Alloys with Ge, Sn and Ti Additions (at.%)
Source: Materials (Basel). 2020 Oct 13;13(20):4548. doi: 10.3390/ma13204548 (PMC7602085; doi:10.3390/ma13204548)
Supplement: Supplementary file 1 [file materials-13-04548-s001.pdf]

materials

MDPI

Article

On the Microstructure and Properties of Nb-18Si-6Mo-5Al-5Cr-2.5W-1Hf Nb-Silicide Based Alloys with Ge, Sn and Ti Additions (at.%)

Jiang Zhao [1,2], Claire Utton [1] and Panos Tsakiropoulos [1,*]

[1] Department of Materials Science and Engineering, Sir Robert Hadfield Building, The University of Sheffield, Mappin Street, Sheffield S1 3JD, UK; zhaojiang6325@hotmail.com (J.Z.); c.utton@sheffield.ac.uk (C.U.)

[2] College of Materials and Chemical Engineering, Key Laboratory of Inorganic Nonmetallic Crystalline and Energy Conversion Materials, Three Gorges University, Yichang 443002, China

* Correspondence: p.tsakiropoulos@sheffield.ac.uk

Received: 23 September 2020; Accepted: 6 October 2020; Published: 13 October 2020

Table S1. EDS analysis data (at.%) of the alloy JZ4.

	Nb	Ti	Si	Mo	W	Sn	Ge	Hf	Al	Cr
					As cast					
Top[a]	39.4 ± 0.3<br>39.1–39.7	12.4 ± 0.3<br>12.2–12.7	18.2 ± 0.7<br>17.5–19.1	6.0 ± 0.3<br>5.7–6.4	2.5 ± 0.1<br>2.4–2.7	5.3 ± 0.3<br>4.8–5.6	5.2 ± 0.2<br>4.9–5.5	1.1 ± 0.1<br>0.9–1.3	4.9 ± 0.3<br>4.6–5.2	5.0 ± 0.3<br>4.7–5.4
Bulk[a]	39.8 ± 0.4<br>39.3–40.3	12.1 ± 0.2<br>11.9–12.5	18.7 ± 0.3<br>18.1–19.0	6.0 ± 0.4<br>5.7–6.6	2.3 ± 0.2<br>2.1–2.6	5.3 ± 0.3<br>4.9–5.6	5.4 ± 0.1<br>5.3–5.5	1.0 ± 0.1<br>0.9–1.1	4.6 ± 0.3<br>4.2–5.0	4.8 ± 0.3<br>4.5–5.1
Bottom[a]	38.2 ± 0.3<br>37.9–38.6	12.9 ± 0.1<br>12.8–13.1	16.6 ± 0.41<br>6.1–17.2	6.5 ± 0.3<br>6.2–6.8	1.9 ± 0.2<br>1.8–2.3	6.6 ± 0.2<br>6.5–7.0	5.0 ± 0.3<br>4.5–5.4	1.1 ± 0.1<br>1.0–1.2	5.5 ± 0.2<br>5.2–5.7	5.7 ± 0.3<br>5.5–6.2
Nb_5Si_3	45.1 ± 0.2<br>44.8–45.2	9.6 ± 0.2<br>9.4–10.0	28.2 ± 0.5<br>27.5–28.5	4.5 ± 0.2<br>4.2–4.8	1.7 ± 0.1<br>1.6–1.9	1.2 ± 0.1<br>1.2–1.4	5.7 ± 0.2<br>5.5–5.9	0.7 ± 0.1<br>0.6–0.9	1.9 ± 0.2<br>1.7–2.0	1.4 ± 0.1<br>1.2–1.5
Ti-rich Nb_5Si_3	40.7 ± 1.5<br>38.3–42.3	12.8 ± 1.1<br>11.7–14.6	20.8 ± 1.1<br>18.9–21.6	4.7 ± 0.1<br>4.5–4.9	1.3 ± 0.2<br>0.9–1.5	5.0 ± 1.0<br>4.2–6.8	5.9 ± 0.3<br>5.5–6.2	1.5 ± 0.2<br>1.3–1.8	4.6 ± 0.4<br>4.2–5.3	2.7 ± 0.5<br>2.3–3.5
A15-Nb_3X	42.1 ± 0.3<br>41.7–42.4	10.7 ± 0.2<br>10.4–10.9	2.5 ± 0.3<br>2.2–3.0	14.2 ± 0.3<br>13.8–14.5	8.3 ± 0.3<br>8.0–8.3	9.1 ± 0.1<br>9.0–9.3	1.6 ± 0.2<br>1.4–1.9	–	6.6 ± 0.2<br>6.5–7.0	4.9 ± 0.1<br>4.8–5.0
Ti-rich A15	29.8 ± 2.3<br>29.0–32.6	15.1 ± 1.0<br>14.1–16.3	1.1 ± 0.6<br>0.4–1.9	14.4 ± 1.2<br>12.8–14.9	4.6 ± 0.4<br>4.3–5.4	12.5 ± 1.4<br>11.2–14.5	0.9 ± 0.1<br>0.6–1.0	0.2 ± 0.1<br>0.0–0.4	9.4 ± 0.7<br>8.7–10.4	12.0 ± 1.2<br>10.7–13.8
TM_5Sn_2X	23.9<br>22.8	27<br>27.4	1.8<br>2.6	4.6<br>4.6	0.6<br>0.5	25.7<br>23.8	1.6<br>2.1	2.1<br>2.2	8<br>8.1	5<br>5.9
C14-Cr_2Nb	22.3 ± 0.6<br>21.6–23.2	6.1 ± 0.3<br>5.6–6.5	7.7 ± 0.9<br>6.9–9.0	4.9 ± 0.1<br>4.7–5.0	3.4 ± 0.3<br>3.0–3.7	1.1 ± 0.3<br>0.7–1.4	1.3 ± 0.2<br>1.1–1.5	2.0 ± 0.3<br>1.6–2.3	9.4 ± 1.2<br>7.3–10.4	41.8 ± 1<br>40.4–43.1
					Heat treated					
Average composition[a]	39.9 ± 0.3<br>39.4–40.2	12.3 ± 0.2<br>11.9–12.5	18.2 ± 0.3<br>17.9–18.5	5.8 ± 0.3<br>5.6–6.3	2.2 ± 0.1<br>2.1–2.3	5.4 ± 0.3<br>5.0–5.4	5.4 ± 0.3<br>5.1–5.8	1.2 ± 0.1<br>1.0–1.4	4.8 ± 0.2<br>4.6–4.9	4.8 ± 0.2<br>4.6–5.0
$(Nb,W)_{ss}$	26.7 ± 0.2<br>26.5–27.0	5.4 ± 0.1<br>5.2–5.5	0	21.8 ± 0.3<br>21.3–22.2	29.4 ± 0.2<br>29.2–29.6	0.8 ± 0.1<br>0.7–0.9	0.1	0.6	3.2 ± 0.1<br>3.1–3.5	12.0 ± 0.1<br>11.8–12.2
Nb_5Si_3	39.8 ± 0.2<br>39.6–40.1	13.4 ± 0.2<br>13.2–13.5	20.8 ± 0.2<br>20.6–21.0	4.3 ± 0.3<br>4.0–4.7	0.6 ± 0.2<br>0.4–0.8	5.3 ± 0.1<br>5.2–5.5	5.7 ± 0.1<br>5.5–5.8	1.3 ± 0.1<br>1.2–1.4	4.8 ± 0.1<br>4.6–5.0	4.0 ± 0.1<br>3.9–4.1
Ti-rich Nb_5Si_3	34.7 ± 0.4<br>34.2–35.1	15.3 ± 0.3<br>14.9–15.7	24.1 ± 0.5<br>23.4–24.6	3.7 ± 0.3<br>3.3–4.0	–	1.6 ± 0.2<br>0.4–1.8	6.8 ± 0.1<br>6.6–6.9	4.3 ± 0.2<br>4.0–4.5	5.6 ± 0.1<br>5.5–5.7	3.9 ± 0.2<br>3.8–4.2
A15-Nb_3X	39.3 ± 0.1<br>39.2–39.5	10.6 ± 0.2<br>10.4–11.0	2.0 ± 0.2<br>1.7–2.2	14.3 ± 0.2<br>14.1–14.3	5.6 ± 0.1<br>5.4–5.7	10.3 ± 0.1<br>10.2–10.5	1.2 ± 0.1<br>1.0–1.4	0.1	7.1 ± 0.1<br>7.0–7.0	9.5 ± 0.3<br>9.0–9.8
C14-Cr_2Nb	25.9 ± 0.4<br>25.4–26.2	4.0 ± 0.1<br>3.8–4.1	10.2 ± 0.3<br>9.8–10.5	4.0 ± 0.3<br>3.7–4.2	3.5 ± 0.3<br>3.3–3.7	0.3	1.0 ± 0.2<br>0.9–1.2	1.3 ± 0.1<br>1.2–1.4	4.4 ± 0.2<br>4.2–4.7	45.4 ± 0.7<br>44.7–46.1

Nb_5Si_3	39.8 ± 0.2<br>39.6–40.1	13.4 ± 0.2<br>13.2–13.5	20.8 ± 0.2<br>20.6–21.0	4.3 ± 0.3<br>4.0–4.7	0.6 ± 0.2<br>0.4–0.8	5.3 ± 0.1<br>5.2–5.5	5.7 ± 0.1<br>5.5–5.8	1.3 ± 0.1<br>1.2–1.4	4.8 ± 0.1<br>4.6–5.0	4.0 ± 0.1<br>3.9–4.1
Ti-rich Nb_5Si_3	34.7 ± 0.4<br>34.2–35.1	15.3 ± 0.3<br>14.9–15.7	24.1 ± 0.5<br>23.4–24.6	3.7 ± 0.3<br>3.3–4.0	–	1.6 ± 0.2<br>0.4–1.8	6.8 ± 0.1<br>6.6–6.9	4.3 ± 0.2<br>4.0–4.5	5.6 ± 0.1<br>5.5–5.7	3.9 ± 0.2<br>3.8–4.2
A15-Nb_3X	39.3 ± 0.1<br>39.2–39.5	10.6 ± 0.2<br>10.4–11.0	2.0 ± 0.2<br>1.7–2.2	14.3 ± 0.2<br>14.1–14.3	5.6 ± 0.1<br>5.4–5.7	10.3 ± 0.1<br>10.2–10.5	1.2 ± 0.1<br>1.0–1.4	0.1	7.1 ± 0.1<br>7.0–7.0	9.5 ± 0.3<br>9.0–9.8
C14-Cr_2Nb	25.9 ± 0.4<br>25.4–26.2	4.0 ± 0.1<br>3.8–4.1	10.2 ± 0.3<br>9.8–10.5	4.0 ± 0.3<br>3.7–4.2	3.5 ± 0.3<br>3.3–3.7	0.3	1.0 ± 0.2<br>0.9–1.2	1.3 ± 0.1<br>1.2–1.4	4.4 ± 0.2<br>4.2–4.7	45.4 ± 0.7<br>44.7–46.1

a = large area analysis.

Table S2. EDS analysis data (at.%) for the alloy JZ5.

	Nb	Ti	Si	Mo	W	Sn	Ge	Hf	Al	Cr
					As cast					
Top[a]	31.7 ± 0.6<br>31.2–32.4	20.6 ± 0.6<br>20.0–21.5	19.4 ± 1.0<br>18.4–20.9	6.3 ± 0.2<br>6.0–6.6	1.3 ± 0.2<br>1.0–1.5	5.4 ± 0.3<br>5.0–5.8	5.2 ± 0.1<br>5.1–5.3	0.9 ± 0.1<br>0.8–1.0	4.5 ± 0.3<br>4.2–5.0	4.7 ± 0.3<br>4.2–4.9
Bulk[a]	32.8 ± 1.4<br>31.1–33.9	19.9 ± 0.6<br>19.3–20.7	19.8 ± 1.9<br>17.2–21.7	6.3 ± 0.5<br>6.0–7.1	1.3 ± 0.3<br>1.0–1.6	5.3 ± 1.0<br>4.5–6.4	5.3 ± 0.1<br>5.2–5.5	0.8 ± 0.1<br>0.7–0.9	4.1 ± 0.5<br>3.6–4.7	4.4 ± 0.5<br>3.9–5.1
Bottom[a]	31.5 ± 0.7<br>30.3–32.2	20.6 ± 0.5<br>20.2–21.2	18.3 ± 0.9<br>17.3–19.2	6.3 ± 0.1<br>6.1–6.4	0.8 ± 0.1<br>0.7–0.9	6.5 ± 0.4<br>6.0–6.9	5.1 ± 0.4<br>4.7–5.4	0.9 ± 0.1<br>0.8–1.0	4.8 ± 0.4<br>4.4–5.2	5.2 ± 0.7<br>4.7–6.4
Nb_5Si_3	37.9 ± 0.5<br>3.73–38.6	16.9 ± 0.5<br>16.3–17.4	28.1 ± 0.6<br>27.5–28.8	4.9 ± 0.2<br>4.5–5.1	1.0 ± 0.1<br>1.0–1.1	1.2 ± 0.2<br>0.9–1.5	6.2 ± 0.2<br>5.7–6.3	0.8 ± 0.1<br>0.7–0.9	1.6 ± 0.2<br>1.4–1.8	1.4 ± 0.1<br>1.3–1.4
Ti-rich Nb_5Si_3	31.1 ± 0.6<br>30.2–31.6	21.9 ± 0.6<br>21.2–22.6	18.3 ± 0.3<br>17.8–18.6	5.3 ± 0.2<br>5.0–5.5	1.3 ± 0.1<br>1.2–1.4	6.2 ± 0.2<br>6.1–6.5	6.6 ± 0.1<br>6.5–6.8	1.3 ± 0.2<br>1.1–1.5	5.0 ± 0.1<br>4.9–5.1	3.0 ± 0.2<br>2.8–3.2
A15-Nb_3X	30.1 ± 0.7<br>29.2–31.2	19.4 ± 0.3<br>19.1–19.9	2.1 ± 0.4<br>1.8–2.7	16.2 ± 0.4<br>15.9–16.8	4.8 ± 0.2<br>15.9–16.8	10.3 ± 0.5<br>9.5–10.7	2.1 ± 0.4<br>1.8–2.7	0.5	7.1 ± 0.2<br>6.9–7.5	7.4 ± 0.5<br>7.0–8.1
Ti-rich A15	19.1 ± 2.6<br>16.3–22.4	22.7 ± 1.4<br>21.2–24.5	0.8 ± 0.3<br>0.4–1.1	15.5 ± 0.4<br>15.0–15.9	3.9 ± 0.5<br>3.4–4.5	11.6 ± 0.8<br>10.6–12.3	1.4 ± 0.3<br>1.0–1.8	0.4	10.5 ± 1.0<br>9.3–11.7	14.1 ± 0.9<br>13.1–15.5
TM_5Sn_2X	13.7<br>11.4	34.8<br>37.5	3.6<br>3	3.4<br>3.4	–<br>–	24.8<br>24.6	2.9<br>2.7	1.2<br>1.5	8.2<br>8.4	7.4<br>7.5

C14-Cr_2Nb	18.5 ± 0.3<br>18.3–19.1	10.5 ± 0.2<br>10.3–10.7	7.5 ± 0.7<br>6.7–8.3	5.0 ± 0.4<br>4.6–5.6	2.3 ± 0.2<br>2.1–2.6	0.9 ± 0.4<br>0.5–1.6	1.4 ± 0.2<br>1.2–1.8	1.4 ± 0.1<br>1.4–1.6	9.3 ± 1.3<br>7.8–10.6	43.2 ± 0.4<br>42.8–43.8
Heat treated										
Average composition[a]	31.4 ± 0.3<br>31.1–31.8	20.5 ± 0.2<br>20.4–20.8	18.8 ± 0.4<br>18.4–19.3	6.2 ± 0.1<br>6.0–6.4	1.2 ± 0.1<br>1.0–1.3	5.7 ± 0.2<br>5.4–5.9	5.6 ± 0.2<br>5.4–5.9	1.0 ± 0.2<br>0.8–1.3	4.7 ± 0.2<br>4.6–5.0	4.9 ± 0.2<br>4.7–5.1
$(Nb,W)_{ss}$	21.2 ± 0.4<br>20.9–21.7	11.3 ± 0.3<br>10.8–11.6	0	23.4 ± 0.3<br>22.9–23.9	18.0 ± 0.3<br>17.7–18.3	1.2 ± 0.1<br>1.0–1.4	1.6 ± 0.6<br>0.8–2.3	0.3	5.3 ± 0.3<br>5.1–5.8	17.7 ± 0.3<br>17.2–18.0
Nb_5Si_3	31.4 ± 0.2<br>31.2–31.6	21.1 ± 0.4<br>20.6–21.7	20.3 ± 0.5<br>19.5–20.9	5.0 ± 0.1<br>4.9–5.2	0.5	5.7 ± 0.3<br>5.3–6.1	6.2 ± 0.3<br>5.9–6.8	1.1 ± 0.1<br>1.1–1.2	4.6 ± 0.1<br>4.5–4.8	4.1 ± 0.2<br>3.8–4.2
Ti-rich Nb_5Si_3	27.4 ± 0.3<br>26.9–27.7	23.7 ± 0.4<br>23.1–24.2	24.1 ± 0.3<br>23.6–24.4	3.8 ± 0.3<br>3.6–4.4	–	1.4 ± 0.1<br>1.3–1.5	7.2 ± 0.1<br>7.1–7.3	3.2 ± 0.1<br>3.1–3.4	5.2 ± 0.1<br>5.1–5.3	4.0 ± 0.2<br>3.7–4.2
A15-Nb_3X	29.9 ± 0.4<br>29.3–30.4	17.4 ± 0.3<br>16.9–17.5	2.4 ± 0.2<br>2.0–2.6	16.3 ± 0.3<br>15.9–16.6	4.7 ± 0.1<br>4.5–4.8	9.2 ± 0.1<br>9.2–9.3	1.8 ± 0.1<br>1.7–2.0	0.2	7.3 ± 0.1<br>7.2–7.4	10.8 ± 0.6<br>10.2–11.7

a = Large area analysis.

Table S3. EDS analysis data (at.%) of phases in the alloy JZ4 after oxidation at 800 °C for 100 h.

Phase	O	Nb	Ti	Si	Mo	W	Sn	Ge	Hf	Al	Cr
Oxide scale											
Mixed Si-rich oxide	70.8 ± 1.4<br>69.0–72.4	13.4 ± 0.8<br>12.1–14.1	3.2 ± 0.1<br>3.0–3.3	7.7 ± 0.8<br>6.6–8.8	1.3 ± 0.3<br>0.8–1.6	0.5	0.4	0.8 ± 0.5<br>0.3–1.4	0.3	0.9 ± 0.2<br>0.7–1.0	0.8 ± 0.1<br>0.7–1.0
Bulk											
Nb_5Si_3	–	45.0 ± 0.8<br>44.2–45.9	9.6 ± 0.1<br>9.5–9.8	28.1 ± 0.4<br>27.5–28.5	4.4 ± 0.3<br>3.9–4.6	1.4 ± 0.2<br>1.1–1.6	1.1 ± 0.1<br>0.9–1.2	6.4 ± 0.6<br>6.2–7.0	1.0 ± 0.1<br>0.8–1.1	1.9 ± 0.3<br>1.5–2.3	1.2 ± 0.2<br>0.9–1.4
Ti-rich Nb_5Si_3	–	39.9 ± 2.2<br>38.2–43.6	14.2 ± 1.6<br>11.5–15.4	20.1 ± 0.5<br>19.4–20.7	4.4 ± 0.1<br>4.2–4.6	1.0 ± 0.3<br>0.7–1.5	5.1 ± 0.9<br>3.7–6.1	5.9 ± 0.6<br>5.6–6.9	1.6 ± 0.2<br>1.3–1.9	4.9 ± 0.2<br>4.7–5.1	2.9 ± 0.6<br>2.0–3.6
TM_5Sn_2X	–	23.8	27.9	3.4	3.7	–	24.9	2.1	2.8	7.3	4.1
A15-Nb_3X	–	41.9 ± 1.5<br>39.7–43.0	11.6 ± 0.8<br>10.5–12.6	2.6 ± 0.4<br>2.1–3.0	14.2 ± 0.3<br>13.9–14.6	6.7 ± 0.4<br>6.2–7.3	8.6 ± 0.5<br>8.0–9.1	2.1 ± 0.2<br>1.9–2.3	0.5 ± 0.2<br>0.4–0.8	6.5 ± 0.4<br>5.9–6.8	5.4 ± 0.8<br>4.5–6.6

C14-Cr_2Nb	–	22.2 ± 0.3	7.4 ± 0.4	8.1 ± 0.6	4.8 ± 0.2	2.6 ± 0.2	0.6 ± 0.1	1.7 ± 0.2	2.9 ± 0.1	8.2 ± 1.0	41.6 ± 0.8
		21.6–22.5	7.0–8.1	7.3–8.9	4.7–5.1	2.2–2.8	0.5–0.8	1.5–2.1	2.8–3.1	7.2–9.8	40.4–42.4

Table S4. EDS analysis data (at.%) of phases in the alloy JZ5 after oxidation at 800 °C for 100 h.

Phase	O	Nb	Ti	Si	Mo	W	Sn	Ge	Hf	Al	Cr
Oxide scale											
Mixed Si-rich oxide	72.3 ± 1.0	10.5 ± 1.1	5.1 ± 0.3	7.1 ± 1.0	1.3 ± 0.2	0.3	0.5	1.5 ± 0.5	0.2	0.6	0.7
	71.4–74.0	8.7–11.4	4.8–5.7	5.2–7.7	1.0–1.4			1.1–2.3			
Bulk											
Nb_5Si_3	–	39.8 ± 0.2	15.7 ± 0.2	29.4 ± 0.5	4.8 ± 0.2	1.0 ± 0.1	1.0 ± 0.1	5.3 ± 0.2	0.7 ± 0.1	1.2 ± 0.2	1.0 ± 0.1
		39.4–40.0	15.5–15.9	29.3–29.8	4.6–5.2	0.9–1.1	0.7–1.1	5.1–5.6	0.6–0.8	1.1–1.4	0.9–1.1
Ti-rich Nb_5Si_3	–	33.7 ± 0.8	20.4 ± 0.4	19.7 ± 0.2	5.6 ± 0.3	0.9 ± 0.1	5.7 ± 0.3	5.5 ± 0.2	1.1 ± 0.1	4.8 ± 0.2	2.5 ± 0.4
		32.5–34.3	20.1–21.0	19.5–19.9	5.3–6.1	0.7–1.0	5.3–6.2	5.3–5.7	0.8–1.2	4.6–5.1	2.3–3.1
TM_5Sn_2X	–	12.5	37.1	4.2	3.6	–	25.1	2.8	1.6	8.1	5
A15-Nb_3X	–	32.2 ± 0.3	18.3 ± 0.3	2.7 ± 0.2	16.6 ± 0.2	5.4 ± 0.1	9.4 ± 0.1	2.0 ± 0.4	0.3 ± 0.1	6.6 ± 0.1	6.4 ± 0.2
		31.6–32.5	17.9–18.8	2.5–2.9	16.5–16.9	5.3–5.5	9.3–9.5	1.6–2.5	0.1–0.4	6.5–6.8	6.2–6.7
C14-Cr_2Nb	–	20.0 ± 0.3	10.9 ± 0.4	8.3 ± 0.4	5.2 ± 0.2	2.5 ± 0.2	0.7 ± 0.1	1.3 ± 0.2	1.6 ± 0.1	8.4 ± 0.7	41.2 ± 0.4
		19.4–20.2	10.4–11.4	8.0–8.9	5.0–5.5	2.3–2.7	0.6–0.9	1.1–1.6	1.5–1.8	7.7–9.4	40.5–41.5

Table S5. EDS analysis data (at.%) of phases in the alloy JZ4 after oxidation at 1200 °C for 100 h.

	Phase	O	Nb	Ti	Si	Mo	W	Sn	Ge	Hf	Al	Cr
Oxide scale	Nb-rich oxide	74.2 ± 0.4	19.7 ± 0.5	2.9 ± 0.1	0.7	0.2	1.0 ± 0.1	–	–	0.3	0.7	0.2
		73.5–74.6	19.0–20.5	2.8–3.1			1.0–1.1					
	Ti-rich oxide	71.7 ± 0.3	9.6 ± 0.5	8.5 ± 0.6	1.0 ± 0.7	1.2 ± 1.0	–	–	0.3	0.5	3.4 ± 0.1	3.7 ± 0.6
		71.0–71.9	8.9–10.2	7.6–9.1	0.3–2.1	0.4–2.8					3.3–3.6	2.8–4.2
Diffusion zone	Nb_5Si_3	–	44.0 ± 0.5	10.3 ± 0.1	24.7 ± 0.5	4.9 ± 0.2	1.3 ± 0.3	2.6 ± 0.1	6.3 ± 0.2	1.0 ± 0.2	3.3 ± 0.5	1.6 ± 0.2
			43.3–44.5	10.2–10.5	24.1–25.4	4.7–5.1	0.8–1.6	2.5–2.8	6.1–6.4	0.7–1.1	2.7–3.7	1.4–2.0
	$Nb_5(Si,Ge)_3$	–	42.5 ± 2.2	9.0 ± 1.0	13.2 ± 1.0	7.3 ± 0.7	0.3	9.7 ± 3.1	11.5 ± 2.0	0.3	2.7 ± 0.3	3.4 ± 0.2
			39.2–45.3	7.7–10.3	11.9–14.7	6.4–8.1		7.2–14.6	8.8–13.5		2.5–3.2	3.1–3.6

	$Nb_5(Si,Ge)_3$	–	42.9 ± 2.6	8.5 ± 0.9	11.8 ± 5.0	7.9 ± 1.6	1.5 ± 0.7	–	23.8 ± 4.8	0.8 ± 0.3	0.5 ± 0.1	2.4 ± 0.6
			39.4–45.0	7.7–9.7	7.2–17.4	6.2–9.7	0.5–2.0		18.4–28.0	0.4–1.1	0.4–0.6	1.5–3.0
	A15-Nb_3X	–	41.7 ± 1.1	4.1 ± 0.1	1.5 ± 0.1	21.9 ± 1.1	2.5 ± 0.2	20.0 ± 0.7	2.1 ± 0.5	–	1.1 ± 0.3	5.3 ± 0.3
			40.2–43.2	4.0–4.2	1.4–1.7	20.5–23.2	2.2–2.8	19.1–20.9	1.4–2.9		0.7–1.4	4.8–5.7
	$(Nb,W)_{ss}$	–	16.5 ± 2.3	1.7 ± 0.7	0	29.3 ± 2.7	43.3 ± 3.6	0.8 ± 0.4	2.6 ± 0.4	0.2	0.9 ± 0.3	4.8 ± 0.4
			14.0–19.8	0.8–2.8		26.7–32.6	38.9–47.8	0.4–1.3	2.1–3.1		0.6–1.4	4.5–5.4
Bulk	Nb_5Si_3	–	44.4 ± 0.2	10.3 ± 0.2	24.9 ± 0.9	4.9 ± 0.3	1.6 ± 0.1	2.1 ± 0.1	6.2 ± 0.5	1.0 ± 0.2	3.0 ± 0.2	1.6 ± 0.1
			44.2–44.7	10.0–10.7	24.6–25.0	4.3–5.0	1.5–1.8	2.0–2.3	5.4–6.4	0.8–1.2	2.7–3.2	1.4–1.7
	Ti-rich Nb_5Si_3	–	36.1 ± 2.0	19.4 ± 2.1	13.9 ± 2.3	3.6 ± 0.5	0.4	11.5 ± 2.3	5.0 ± 0.4	2.0 ± 0.3	5.5 ± 0.3	2.6 ± 0.3
			33.0–39.2	15.7–21.6	11.6–17.7	2.8–4.1		7.9–13.5	4.5–5.7	1.6–2.3	5.3–5.9	2.0–2.9
	A15-Nb_3X	–	40.8 ± 0.7	10.2 ± 0.3	2.2 ± 0.3	15.3 ± 0.6	7.7 ± 0.2	8.8 ± 0.4	1.8 ± 0.4	0.3	7.3 ± 0.3	5.7 ± 0.2
			39.8–41.4	9.9–10.4	1.8–2.6	14.3–15.8	7.5–8.0	8.4–9.5	1.2–2.2		7.0–7.7	5.5–6.0
	$(Nb,W)_{ss}$	–	23.9 ± 0.8	5.1 ± 0.3	0	24.0 ± 0.5	33.5 ± 1.3	0.6	1.6 ± 0.7	0.3	1.7 ± 0.2	9.2 ± 1.3
			23.0–24.6	4.6–5.4		23.5–24.6	31.6–35.1		0.7–2.1		1.4–1.9	7.4–10.9
	C14-Cr_2Nb	–	26.8 ± 0.7	4.7 ± 0.2	10.5 ± 0.3	3.9 ± 0.2	3.1 ± 0.5	0.5	1.4 ± 0.2	1.8 ± 0.2	5.1 ± 0.2	42.1 ± 0.6
			25.9–27.9	4.5–5.0	10.2–11.0	3.6–4.1	2.5–4.0		1.2–1.6	1.5–2.1	4.9–5.3	41.3–43.0

Table S6. EDS analysis data (at.%) of phases in the alloy JZ5 after oxidation at 1200 °C for 100 h.

	Phase	O	Nb	Ti	Si	Mo	W	Sn	Ge	Hf	Al	Cr
Oxide scale	Nb-rich oxide	74.7 ± 1.1	17.6 ± 1.9	4.8 ± 0.5	0.9 ± 0.7	–	0.4	–	–	0.3	1.0 ± 0.4	0.2
		73.4–76.1	15.4–18.9	4.0–5.2	0–1.4						0.5–1.4	
	Ti-rich oxide	72.5 ± 0.7	8.0 ± 1.1	11.9 ± 1.7	0.7	–	–	0.2	–	0.4	3.9 ± 0.5	2.3 ± 0.4
		71.5–73.6	6.7–9.6	10.4–14.6							3.3–4.5	1.9–2.8
Diffusion zone	Nb_5Si_3	–	40.8 ± 0.2	17.4 ± 0.3	29.4 ± 0.4	5.2 ± 0.2	1.1 ± 1.3	1.3 ± 0.1	1.0 ± 0.3	0.7	1.5 ± 0.2	1.4 ± 0.2
			40.4–41.0	17.1–17.9	28.9–30.0	5.0–5.5	1.1–1.3	1.2–1.5	0.7–1.4		1.3–1.8	1.2–1.7
	$Nb_5(Si,Sn)_3$	–	31.6 ± 1.4	20.3 ± 1.8	13.2 ± 1.9	5.8 ± 0.3	0.2	16.9 ± 1.5	4.4 ± 0.5	0.8 ± 0.2	2.3 ± 0.6	4.5 ± 0.2
			30.3–33.5	17.4–21.7	11.1–16.0	5.6–6.3		15.0–19.0	3.8–5.3	0.6–1.0	1.2–2.8	4.1–4.7
	$Nb_5(Si,Ge)_3$	–	46.6 ± 1.5	3.3 ± 0.9	17.5 ± 4.6	10.3 ± 1.7	1.7 ± 0.3	0.3	16.7 ± 4.1	0.3	0.9 ± 0.5	2.4 ± 0.6
			44.4–47.8	2.5–4.5	10.8–23.3	8.6–12.6	1.4–2.3		11.2–22.7		0.4–1.5	1.8–3.3
	A15-Nb_3X	–	30.9 ± 1.6	9.9 ± 0.6	1.1 ± 0.2	25.2 ± 0.8	3.9 ± 0.7	19.2 ± 0.7	1.3 ± 0.1	–	1.4 ± 0.6	7.1 ± 0.4

			28.3–32.6	9.3–10.7	0.9–1.3	24.2–26.3	3.0–4.9	18.4–20.2	1.1–1.4		0.8–2.4	6.8–7.7
	$(Nb,W)_{ss}$	–	12.7 ± 1.2	3.6 ± 0.6	–	31.5 ± 0.8	39.1 ± 1.3	1.0 ± 0.7	2.2 ± 0.4	0.3	1.6 ± 0.6	8.1 ± 0.9
			11.5–14.5	2.8–4.4		30.9–32.8	38.0–41.0	0.1–1.9	1.9–2.7		0.9–2.6	7.5–9.4
Bulk	Nb_5Si_3	–	39.0 ± 0.8	16.2 ± 0.5	28.1 ± 0.6	5.2 ± 0.2	0.8 ± 0.1	1.3 ± 0.2	5.6 ± 0.4	0.8 ± 0.1	1.6 ± 0.2	1.4 ± 0.1
			37.9–40.0	15.6–16.9	27.4–28.8	5.0–5.4	0.7–0.9	1.1–1.6	5.1–5.9	0.7–0.8	1.5–2.0	1.3–1.6
	Ti-rich Nb_5Si_3	–	28.2 ± 2.6	26.7 ± 3.1	13.9 ± 2.6	4.2 ± 0.8	0.2	11.8 ± 2.9	5.0 ± 0.5	1.2 ± 0.1	5.9 ± 0.3	2.9 ± 0.8
			25.6–32.3	25.8–29.9	10.8–17.7	3.4–5.3		7.7–14.8	4.3–5.7	1.0–1.4	5.4–6.2	2.1–3.7
	A15-Nb_3X	–	27.1 ± 0.9	18.6 ± 0.9	1.4 ± 0.3	19.9 ± 0.8	4.2 ± 0.5	10.6 ± 0.3	1.4 ± 0.3	0.2	8.9 ± 0.3	7.6 ± 1.1
			25.9–28.1	17.7–19.8	1.1–1.9	18.8–20.9	3.6–4.9	10.3–11.2	1.2–1.8		8.5–9.2	6.4–9.0
	$(Nb,W)_{ss}$	–	19.5 ± 0.8	9.7 ± 0.5	–	27.9 ± 0.7	30.0 ± 2.0	1.6 ± 0.6	2.0 ± 0.4	0.4	2.2 ± 0.4	6.8 ± 0.5
			18.4–20.6	9.2–10.2		27.1–28.8	27.7–31.6	1.1–2.3	1.5–2.6		1.8–2.8	6.3–7.3
	C14-Cr_2Nb	–	22.9 ± 0.4	7.8 ± 0.3	8.7 ± 0.3	4.6 ± 0.1	3.3 ± 0.1	0.4	1.0 ± 0.1	1.3 ± 0.1	7.0 ± 0.5	43.0 ± 0.4
			22.5–23.4	7.2–8.1	8.5–9.2	4.5–4.6	3.1–3.5		0.9–1.1	1.2–1.5	6.1–7.3	42.4–43.2

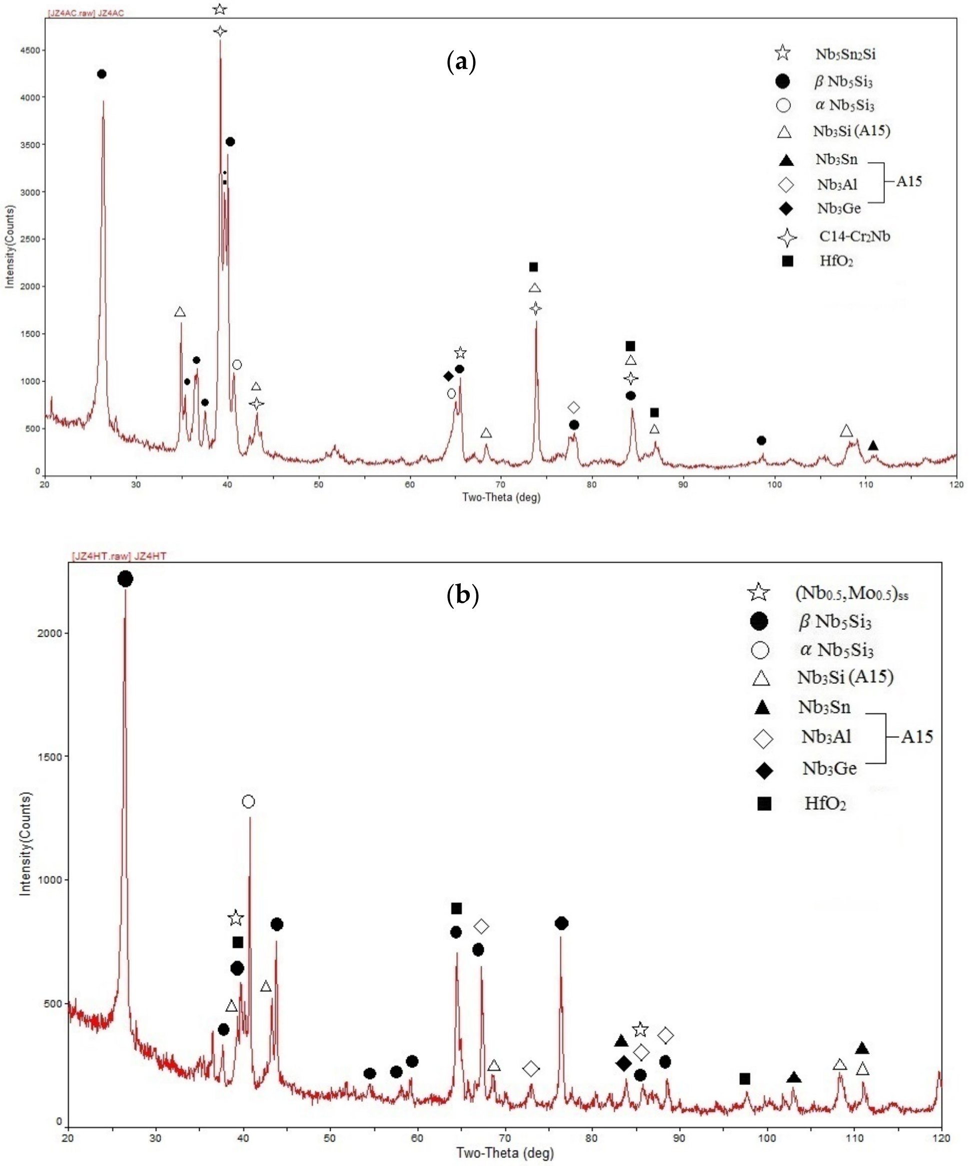

Figure S1. X–ray diffractograms of the (**a**) as cast and (**b**) heat treated alloy JZ4.

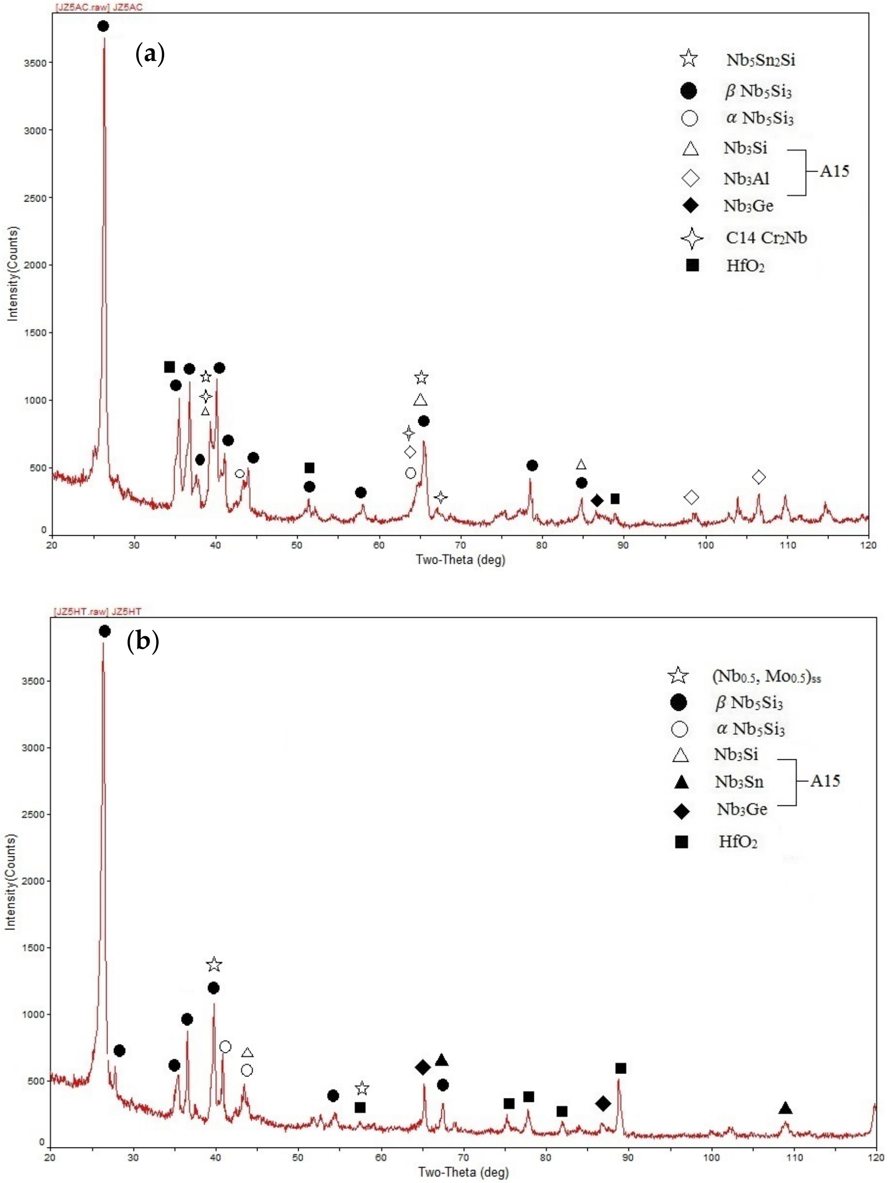

Figure S2. X–ray diffractograms of the (**a**) as cast and (**b**) heat treated alloy JZ5.
